# Sparse X-ray spectro-tomography for high-sensitivity three-dimensional chemical imaging at the nanoscale

**DOI:** 10.1038/s41467-026-77821-5

**Published:** 2026-09-16

**Authors:** Hanfei Yan, Ajith Pattammattel, Aaron Michelson, Enyuan Hu, Kangxuan Xia

**Affiliations:** 1 National Synchrotron Light Source II, Brookhaven National Laboratory, Upton, NY USA; 2 Center for Functional Nanomaterials, Brookhaven National Laboratory, Upton, NY USA; 3 Chemistry Department, Brookhaven National Laboratory, Upton, NY USA

**Keywords:** Applied mathematics, Imaging techniques, Batteries

## Abstract

Achieving three-dimensional (3D) chemical imaging with scanning x-ray microscopy is often hindered by the prohibitively long acquisition time. In this work, we develop a method that drastically reduces the data requirement by two orders of magnitude, thereby significantly decreasing acquisition time and radiation dose. This is accomplished through sparse and stochastic energy sampling combined with a joint spectral-tomographic reconstruction algorithm that performs the spectrum fitting with prior knowledge of the known reference spectra and 3D reconstruction in a single step. Using this approach, we reveal the reduced oxidation state of Co on the surface of a cycled lithium-ion battery particle and its correlation with the depletion of Mn in 3D. This technique enables comprehensive 3D material characterization with high sensitivity and multimodality, opening opportunities in various scientific and technological fields where volumetric chemical information at the nanoscale is essential.

## Abstract (web summary)

The study introduces a sparse spectro‑tomography method that cuts data needs in scanning mode by about two orders of magnitude, enabling high-sensitivity, low‑dose 3D chemical imaging. It uncovers surface Co reduction and Mn loss in battery particles, revealing key degradation pathways.

## Introduction

X-ray absorption spectroscopy (XAS) is a powerful technique that provides information on oxidation state, coordination chemistry, and coordination number of an element^1^. In such a measurement, the spectrum is obtained by measuring either the variation of the absorption coefficient or fluorescence intensity as the incident x-ray energy is changed across the absorption edge of the interested element. When combined with X-ray microscopy, it creates an even more powerful tool that is capable of revealing the local variations at the nanoscale, which is crucial for studies in many different scientific fields such as energy storage system^2–4^, additive manufacturing^5^, catalysis^6,7^, and environmental science^8,9^.

X-ray microscopy can be achieved in two modes: the full-field and scanning modes. The full-field mode provides a fast snapshot but has limited modalities and sensitivity^10^. In contrast, the scanning mode offers a suite of modalities with high sensitivity but is slow due to the raster-scanning nature^11^. Both offer tomography capabilities, where a series of projection images is taken at different angles to form a sinogram for reconstruction. However, for three-dimensional (3D) chemical imaging using spectro-tomography, combining energy scans with angular projections results in an excessive number of tomographic measurements. Conventionally, spectro-tomography requires a full tomography dataset at each energy point, i.e., one sinogram per energy. Considering 50–100 energy points for a spectrum, a tomographic measurement must be finished within a few minutes to limit the total acquisition time in a day for practical applications. As a result, spectro-tomography experiments are mostly conducted in the full-field mode with absorption-contrast^12,13^ because the scanning mode with fluorescence-contrast would take an impractically long measurement time of weeks^2^. There have been efforts made to reduce the measurement time by using sparsity in projections^14^, or sparsity in scanned points of individual images^15^. Using a limited number of energy points based on feature selection is another strategy^16^. These methods significantly reduce the amount of required data but still require an order of magnitude more data than that of a single sinogram.

Here, we propose a sparse spectro-tomography method that does not require more measurements than a single sinogram, thereby reducing the amount of data, measurement time, and radiation dose by two orders of magnitude. We demonstrated that an experiment that would normally take 3 weeks can be completed in 10 hours. This is achieved through sparse and stochastic energy sampling in the energy-angle space and the development of a joint spectro-tomography algorithm that directly reconstructs the chemical states in 3D. With the prior knowledge of chemical states presented in the sample, we reduce the number of unknown spectral variations with energy down to the number of states, thereby requiring fewer data points. A full measurement will still be needed if this dimension reduction cannot be done. We discussed the impact of different energy sampling schemes using simulation data and demonstrated that stochasticity in energy sampling was the key for the method to perform well. A cylindrical sample composed of a mixture of stainless steel and hematite nanoparticles was measured to validate the method’s accuracy and effectiveness.

Lastly, we applied our method to study a cycled lithium-ion battery cathode particle and discovered a surface phase that was known to cause capacity fading. From one measurement with acquisition time no greater than that of a single sinogram, we obtained chemical states of Co and elemental distribution of Co and Mn in 3D. A strong correlation of the reduced oxidation state of Co with the depletion of Mn was observed, which could shed light on the underlying degradation mechanisms. Such comprehensive 3D characterization was previously unattainable, showcasing the technique’s significant power in addressing fundamental material science questions. In addition, the significant reduction of radiation dose is critical for measurements on radiation-sensitive materials.

## Results

### Joint spectro-tomography reconstruction

**Fig. 1:**
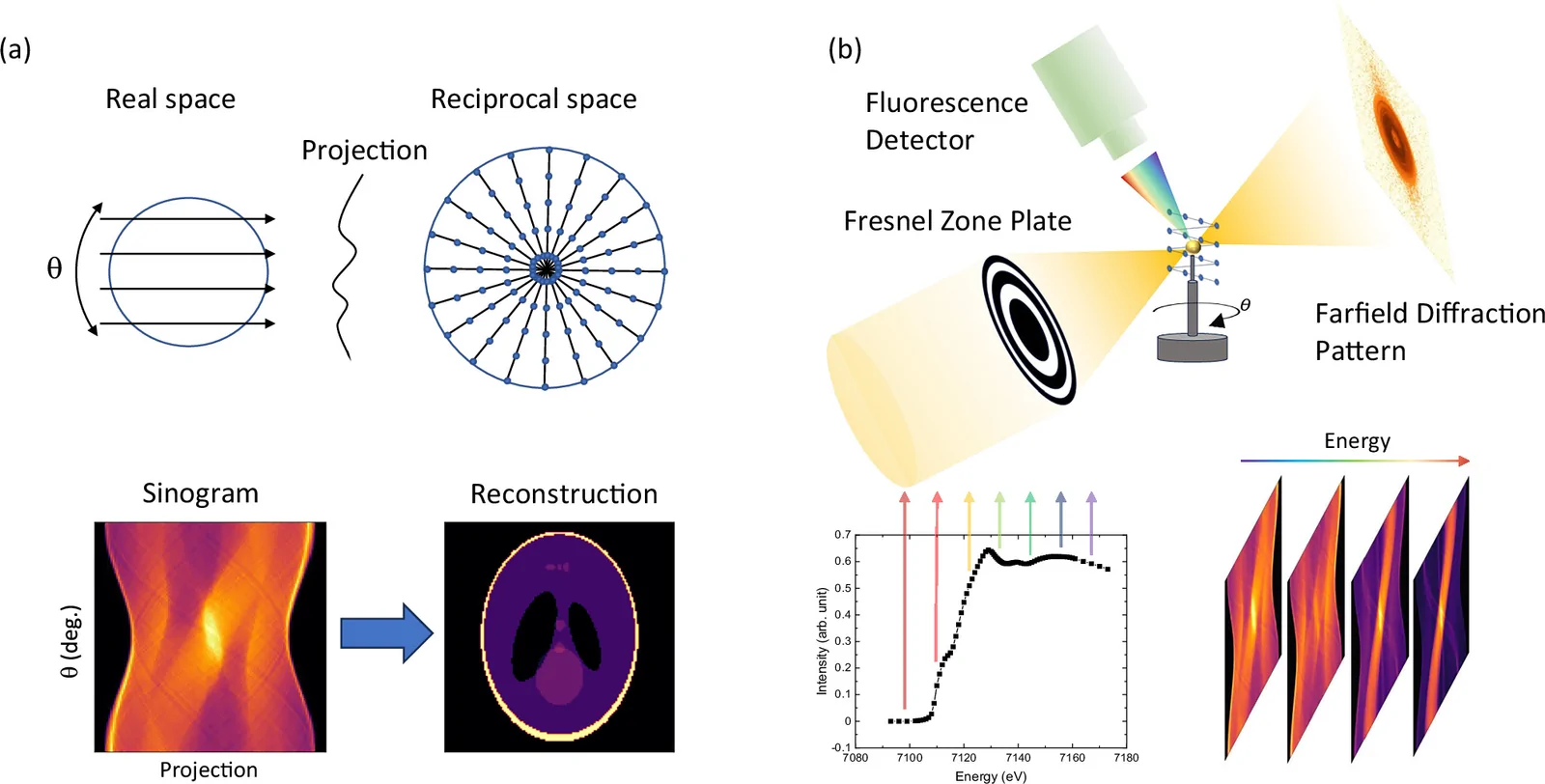
Schematics of parallel-beam tomography and scanning-based spectro-tomography. **a** In conventional parallel-beam tomography, a series of projections acquired as the 2D object rotates forms a sinogram (bottom left). The Fourier transform of each projection corresponds to a radial line in the object’s reciprocal space (top right). When a sufficient number of projections covering 180° are collected, the internal structure can be mathematically reconstructed (bottom right). For 3D objects, reconstruction is typically performed slice by slice. **b** In scanning X-ray microscopy, an X-ray nanobeam—focused by a Fresnel zone plate in this case—raster-scans the sample while it is rotated to acquire projection images. Both fluorescence and transmission signals can be recorded simultaneously. To obtain 3D chemical-state information, a series of sinograms is acquired as the incident beam energy is scanned across the absorption edge of the target element.

Tomography requires acquiring a series of projection images as the sample rotates. These projections form a sinogram, which is processed through a mathematical algorithm to reconstruct the internal structure. To achieve high-quality reconstruction, the Crowther criterion^17^, which defines the required number of projections, needs to be fulfilled so that high frequencies in the reciprocal space are sufficiently sampled (see Fig. 1a). For an object with a size of *D*, to resolve a feature size of *d*, N=πD/d projections evenly sampling the angular space are needed. The Crowther criterion sets the requirement for a sharp and aliasing-free reconstruction. When this condition is not met, iterative algorithms can help alleviate artifacts and distortion by imposing additional constraints to the solution at a cost of increased computation^18^. When a single voxel resolution is needed, the number of projections required is N=Mπ/2, where M is the number of column pixels of the projection image.

Resolving the chemical state requires sampling along the fourth dimension, energy. By tuning the incident x-ray energy across the absorption edge of a target element, an x-ray absorption spectrum is obtained, from which its chemical state can be determined. In this work, we focus on X-ray absorption near-edge structure (XANES), which scans the energy in a range of 100 eV with 50–100 data points. To obtain the XANES spectrum at each reconstructed voxel, the same number of tomographic measurements is required. As shown in Fig. 1b, instead of one sinogram, 50–100 of them are needed for conventional spectro-tomography. If we take 180 projections for a tomography dataset, spectro-tomography would require 9000 projections in total for 50 energy points, taking nearly one week to finish if each projection image takes about one minute. This prohibitively long acquisition time makes spectro-tomography in scanning mode very challenging, if not impractical. Therefore, most spectro-tomography applications are performed in the full-field mode^19^, where a fast snapshot with a fraction of a second can be done. Although recent developments in detector technology have enabled a fast scanning speed of 110 kHz and demonstrated ptychographic imaging with 7 µs exposure time^20^, the scanning speed for fluorescence imaging may still remain limited to the millisecond level due to the photon flux limitation.

Conventionally, spectro-tomography requires volume reconstructions at each energy point, which are then combined to produce a four-dimensional (4D) dataset. The fourth dimension corresponds to an XANES spectrum, which is fitted with reference spectra to produce chemical state information. The resulting linear coefficients are the weights of individual states of interest. Instead of performing reconstruction and fitting separately, we combine them to directly reconstruct the volume distribution of individual chemical states. In this manner, we significantly reduce the number of unknowns in the fourth dimension, from the number of energy points to the number of chemical states. From a mathematical point of view, this means much less data is needed to find a solution.

A tomography reconstruction process can be expressed as seeking a solution to an optimization problem,

1 $$\mathop{{\rm{argmin}}}\limits_{{\boldsymbol{x}}}\frac{1}{2}{\Vert {\bf{A}}{\bf{x}}-{\bf{b}}\Vert }_{2}^{2}+\mu g({\bf{x}})$$

In Eq. (1), **A** is the radon transform matrix, **b** is a vector corresponding to the measured projection data, **x** is the object vector, and μ is a constant controlling the weight of the regularization function, *g*(**x**). The first term quantifies the square error of the forward model to the experimental data, ensuring the fidelity, while the second term enforces certain desired properties of the solution based on prior knowledge. Similarly, we formulate a spectro-tomography problem as follows:

2 $$\mathop{{\rm{argmin}}}\limits_{{{\bf{x}}}_{j}}\frac{1}{2}{\Vert \left(\sum _{j}{{\bf{W}}}_{j}{\bf{A}}{{\bf{x}}}_{j}\right)-{\bf{b}}\Vert }_{2}^{2}+\mu {\sum }_{j}{\Vert {{\bf{x}}}_{j}\Vert }_{{\rm{TV}}},$$

where **W***j* is a diagonal matrix reflecting the change of the *j*th state with energy, and **x**_*j*_ is the coefficient of the *j*th state to be reconstructed. Total variation (TV) regularization is applied to suppress the noise and background fluctuation by imposing sparsity on the derivatives of the solution. As one can see, there is no fundamental difference between Eqs. (1) and (2), suggesting that we can reconstruct an individual state’s spatial distributions in 3D directly from the acquired projection data. Equation (2) can be solved using many standard techniques. Here, we adopt the alternating direction method of multipliers (ADMM) for its robustness and simplicity of implementation^21^. See the method section for the detailed derivation of the equation and the implementation of the algorithm.

### Sampling schemes and simulations

**Fig. 2:**
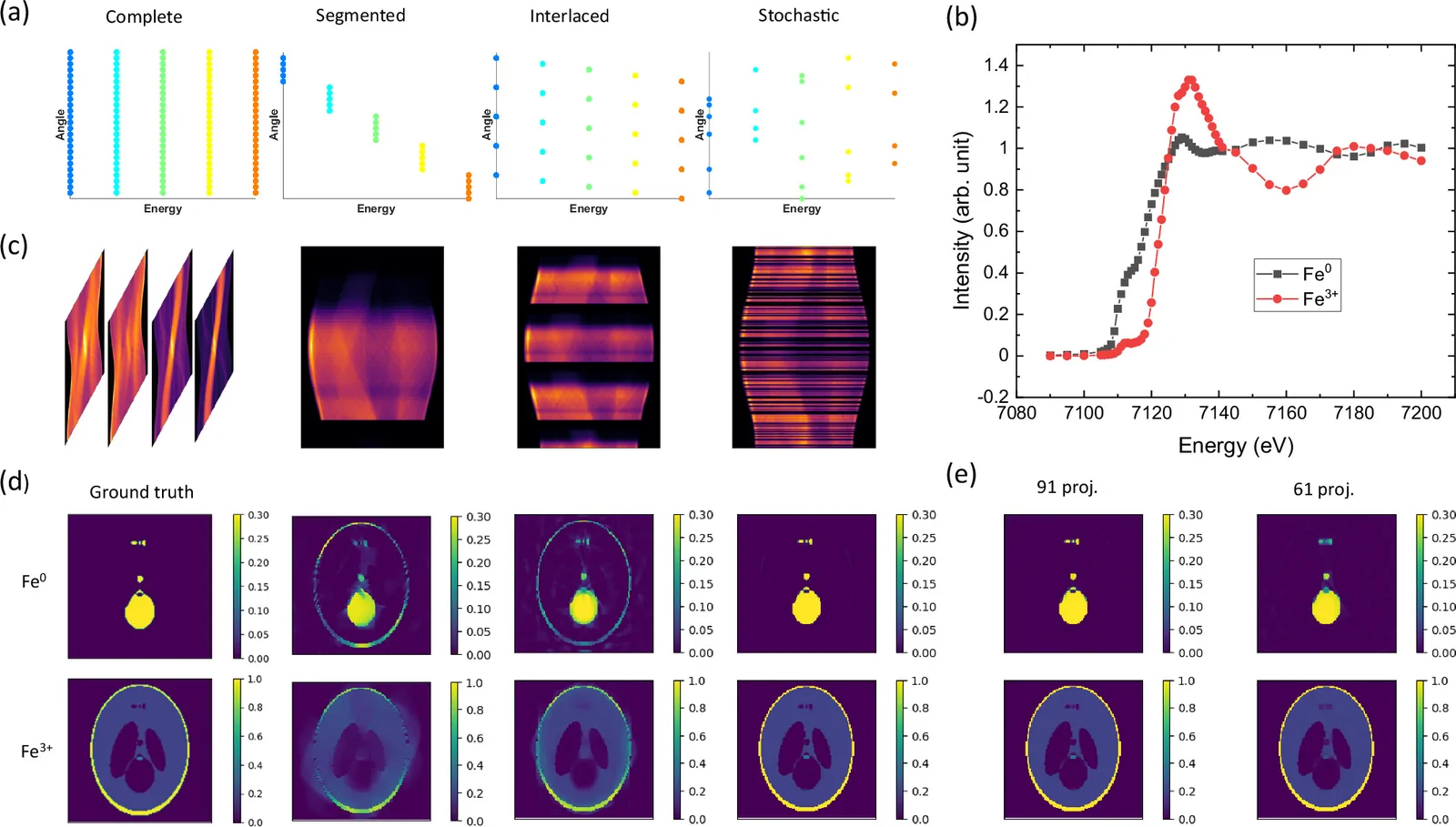
Testing with simulation data. **a** Different sampling schemes in the angle-energy space for spectro-tomography. Each data point represents a projection image acquired at a specific energy. A column corresponds to a conventional tomography dataset (sinogram), while a row represents a 2D nano-XANES dataset collected at a fixed projection angle. **b** XANES spectra of two iron oxidation states, Fe⁰ and Fe³⁺, were used to simulate sinograms of a phantom at various energy points. **c** Data requirements for different sampling schemes. In the conventional approach, the angle-energy space is fully sampled, requiring a separate sinogram for each energy point. In contrast, sparse sampling methods produce a single sinogram where energy varies across projection angles. **d** Spectro-tomography reconstruction of the phantom using each sampling scheme. The phantom size is 128 × 128 pixels. A total of 181 projections were simulated over 180°, with 1° increments. **e** Reconstruction results using the stochastic sampling scheme with numbers of projections decreased to 91 and 61, respectively.

Spectro-tomography requires projections taken at different angles and energy points. Therefore, each projection is associated with two parameters: angle and energy. Fast measurement necessitates a very sparse sampling in the angle-energy parameter space. There are many strategies one can take, as shown in Fig. 2a. When this space is evenly sampled along both axes with grid points, that corresponds to a complete dataset for the conventional method. We can divide the angles into many sub-sections, within each, the same energy applies. This refers to a segmented sampling strategy. Alternatively, we can change the energy as the projection progresses with the angle. In this case, projections with the same energy evenly sample the angular space in an interlaced manner, given that the number of angles is greater than that of energy points. This strategy was adopted by Z. Gao et al. for ptychography-based 3D chemical imaging and demonstrated an order-of-magnitude data reduction^14^. In the last scenario, the angle-energy combination is stochastic—pre-selected energy points are sampled randomly with equal probabilities, while the projection angles are evenly distributed.

We used simulated data to assess the impact of different sparse sampling schemes. A 128 × 128 pixel phantom was employed as the test object, which consists of two states. To mimic a real experiment, we used Fe^0^ and Fe^3+^ XANES spectra to model the intensity variations at different energy points (Fig. 2b). The synthetic sinogram was generated using the ASTRA toolbox^22^ and then processed by the reconstruction algorithm. We tested cases with 181 projections following three sampling schemes, which is about the number of projections required by the Crowther criterion. For stochastic sampling, we also tested reduced numbers of projections down to 91 and 61 projections. Angular projections were evenly distributed in the range of 0°–180°, and 52 energy points were selected. Figure 2c shows sinograms for each case.

As evident from the results shown in Fig. 2d, even with 181 projections for the first two sampling schemes, significant artefacts and crosstalk between the two states persisted. In comparison, stochastic sampling demonstrated superior performance, achieving a reconstruction with no artifacts and crosstalk.

To ensure a fair comparison, the randomized spectrum was also applied to other sampling strategies. Table 1 reports the mean squared error (MSE) for the different cases, and improvements are observed across all of them. Notably, interlaced sampling, when combined with a randomized spectrum, achieves an average MSE that is slightly lower than that of the stochastic angle–energy combination. This indicates that stochastic energy sampling plays a key role in improving reconstruction quality. In this case, the latter two sampling strategies still perform noticeably better than the segmented, although the performance gap is reduced.

**Table 1:** Comparison of the mean square error (MSE) for different angle–energy combination strategies using both regular and randomized spectra

	Segmented	Interlaced	Stochastic
Regular spectra	1.65 × 10^−2^	7.23 × 10^−3^	2.89 × 10^−5^ (1.31 × 10^−5^)
Randomized spectra	3.61 × 10^−3^ (2.35 × 10^−4^)	1.80 × 10^−5^(8.34 × 10^−6^)	

For randomized spectra, 20 trials were conducted to compute the mean and standard deviation, capturing the uncertainty inherent in the random process.

In the following discussion, we use the stochastic angle–energy combination for all simulations and experimental measurements. Note that the interlaced–randomized spectrum scheme could yield results that are equally good, if not slightly better.

Because an iterative reconstruction engine was used, with stochastic sampling, very minor reconstruction artifacts arose until the number of projections was reduced to 61. While reconstruction quality slightly decreased as the number of projections was reduced, the fine features of the two states were still well-resolved with 61 projections. These results highlight the importance of stochastic sampling in the energy parameter space for optimal algorithm performance.

The randomness introduced in the matrix **W***j* helps obtain a solution with less artefacts and crosstalk, and the TV regularization further improves the result with iterations. The combination of the two leads to the convergence to the true solution for an underdetermined problem, not being easily trapped in local minima. This behavior can be understood in the reciprocal space of the reconstructed object. The Fourier transform of each projection corresponds to a radial sampling line in reciprocal space that contains contributions from the individual states. When consecutive energy points are assigned to adjacent projections, the relative contributions of the states remain similar, making the corresponding sampling lines nearly linearly dependent. As a result, an average reciprocal-space map over this region can still produce a small error with respect to the measured data, leading to cross-contamination between states in the reconstructed solution. In the limiting case where the same energy is used for all projections, the states cannot be decoupled at all.

Stochastic energy sampling provides a simple yet effective way to mitigate this problem. By randomly selecting energy points from the spectrum, the probability that adjacent projections have similar state contributions becomes smaller, thereby reducing cross-contamination. However, this strategy does not necessarily minimize the linear dependence between projections; its effectiveness in decoupling chemical states could be either better or worse than that of other sampling directions. Developing an optimal energy-sampling strategy that minimizes inter-projection correlation, including the selection of energy points, remains a subject for future investigation.

Impacts of hyperparameters, noise, randomness, and initial guess and overfitting are discussed in Figs. S1–S6. As current limitations, proper tuning and correct selection of reference spectra known to be present in the sample under investigation are essential for achieving optimal results, especially when the data are noisy.

### Experimental verification on a test sample

To evaluate the robustness and effectiveness of the proposed methodology for real-world applications, we prepared a test sample by mixing stainless steel and hematite nanoparticles. The sample contained two chemical states of iron: Fe^0^ in stainless steel and Fe^3+^ in hematite. Cr, coexisting with Fe^0^ in stainless steel, served as an internal reference for the Fe^0^ state. The experiment was conducted at the hard x-ray nanoprobe (HXN) beamline of the National Synchrotron Light Source II, Brookhaven National Laboratory. A monochromatic beam was focused by a Fresnel zone plate to a nano-spot approximately 40 nm in size. The sample was raster-scanned using the nanobeam to create an image. Fluorescence and transmitted x-ray signals were recorded using an energy-dispersive detector (Vortex, Hitachi) and a pixel-array detector (Eiger2 1 M, Dectris), respectively. Figure 1b illustrates the experimental setup.

We selected 52 energy points from the reference spectra of Fe^0^ and Fe^3+^, which were not evenly spaced (Fig. S4). Pre-edge energy points with low intensity have less impact on the 3D reconstruction, and an optimization of energy-point selection can further improve the performance. We chose the same energy points with that of a nano-XANES measurement at the beamline for experimental convenience and a subsequent comparison of the reconstructed spectra. The experiment involved 181 projections taken from −90° to 90° at 1° increment, with energy values randomly chosen from the 52 points. A weight list was created to record the relative intensity variation of each state based on the reference spectra and was used to construct the **W***j* matrix in Eq. (2).

**Fig. 3:**
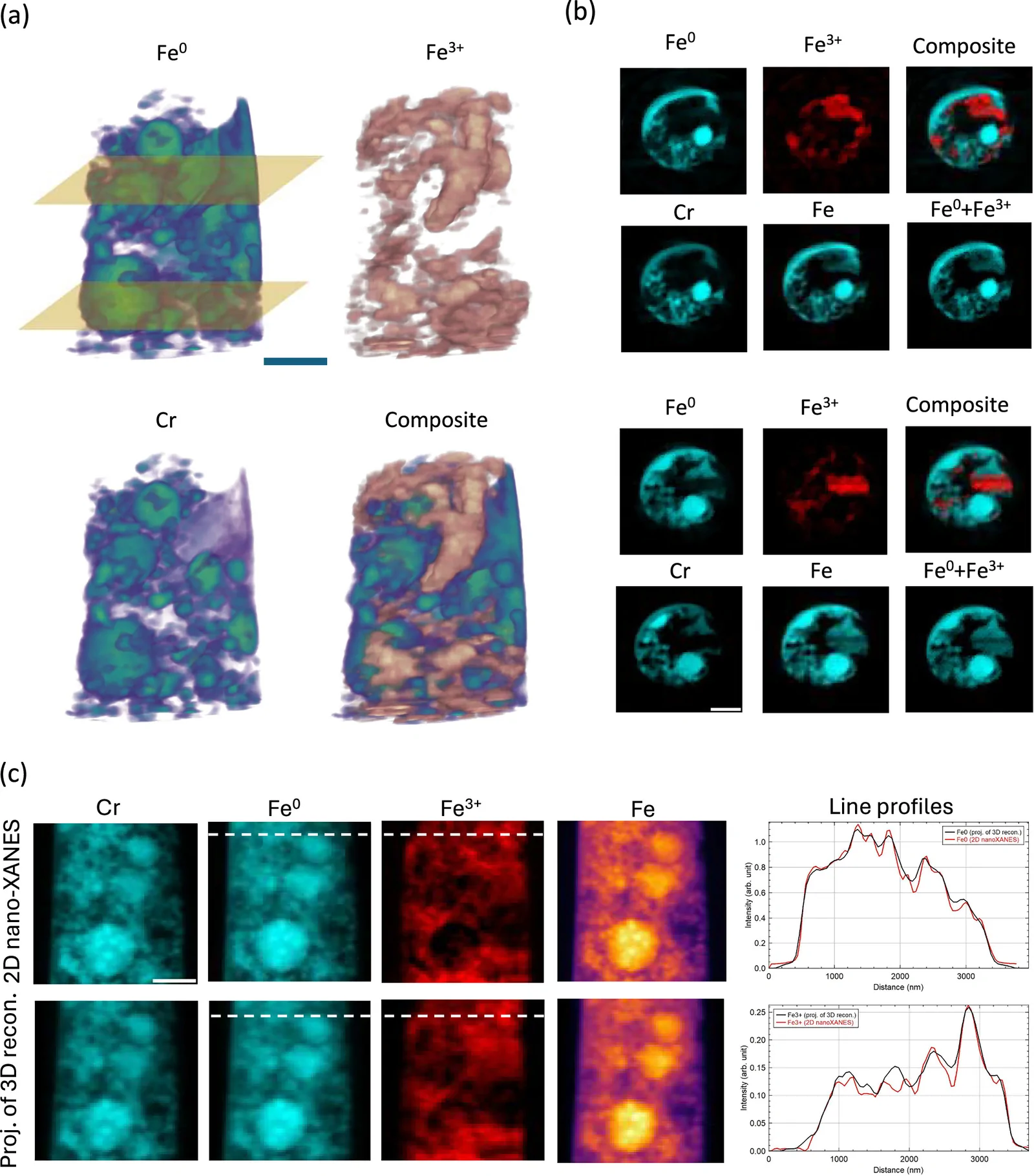
Spectro-tomography reconstruction of a test sample composed of stainless and hematite nanoparticles. **a** 3D rendering showing Fe^0^, Fe^3+^, Cr, and a composite map of the two Fe oxidation states. **b** Two cross-sectional views displaying Fe^0^, Fe^3+^, their composite, Cr, Fe, and the sum of Fe^0^ and Fe^3+^. These results demonstrate a strong spatial correlation between Cr and Fe^0^. As expected, the sum of Fe⁰ and Fe³⁺ closely matches the total Fe distribution. **c** A comparison of the projected 3D reconstruction with an independent 2D nano-XANES measurement at the same angle. The good agreement confirms the accuracy of the 3D reconstruction. Line profiles along the white dashed line are included for quantitative comparison. Minor discrepancies may arise from uncertainties in the spectral fitting process because of noise, particularly for Fe^3+^, whose concentration is about 10% of that of Fe^0^. Scale bar in all images: 1 µm. Source data are provided as a Source Data file.

Figure 3a presents 3D renderings of the volume reconstructions. Since the nanoparticles were mechanically mixed, the two states were expected to be spatially separated, and this was accurately reflected in the results. The distributions of Cr and Fe^0^ were highly correlated, as expected, since Cr is only present in stainless steel nanoparticles. However, a slightly higher concentration of Fe^0^ was observed at the outer boundary of the cylindrical sample. Cross-sectional views at two different heights, shown in Fig. 3b, facilitate comparison and illustrate that Fe^3+^ fills voids in Fe^0^. The distribution of Cr closely matches Fe^0^ except at the outer boundary.

We also compared the total Fe distribution with the sum of Fe^0^ and Fe^3+^ and found them to be identical. This suggests that the excess Fe^0^ at the boundary is real but does not originate from the stainless steel nanoparticles. To verify this observation, an independent 2D nano-XANES measurement was conducted and compared with the projection of the reconstructed state volumes (Fig. 3c). In nano-XANES, the XANES spectrum at each pixel was measured at all selected energy points, providing differentiation of chemical species in 2D. The measurements agreed well across the sample, including at the boundaries. The excess Fe^0^ at the boundary may have been introduced during sample preparation, which involved focused ion beam carving (see the “Methods” section and Figs. S7 and S8). This process can induce localized heating. It is known that Fe^3+^ in hematite can be reduced to Fe^0^ at elevated temperatures^23^. Another measurement on the second sample also exhibited a similar correlation, further confirming the observation (see Fig. S12).

Because spectro‑tomography involves solving a larger number of unknowns, the state map of Fe⁰ exhibits a lower spatial resolution of 80 nm (half‑period), compared with the 50 nm resolution achieved in the Fe reconstruction (also see Fig. S12). Although the iterative method with TV regularization can relax the sampling requirements imposed by the Crowther criterion (Fig. 1e), an increasing ratio of unknowns to knowns adversely affects spatial resolution in the real experiment due to various factors. For example, noise has a greater impact on the resolution of multi-state reconstructions than on that of single-state reconstructions (Fig. S4). More advanced regularization approaches, such as learned deep neural networks or the integration of complementary high-resolution imaging modalities, will be needed to mitigate these limitations.

### Spectro-tomographic reconstruction of an NMC811 particle

With the confidence that the proposed method can reliably discern different chemical states in 3D, we applied it to study the chemical phase changes of a lithium nickel manganese cobalt oxide (NMC811, LiNi₀.₈Mn₀.₁Co₀.₁O₂.) cathode particle. NMC811 is a promising cathode material for electric vehicles due to its high-energy density. However, its high Ni and low Co content can lead to rapid capacity fading and limited cycle life, driven by surface reactions and lattice instability^24,25^. Understanding these degradation mechanisms requires particle-level insight, as Ni, Mn, and Co respond differently to electrochemical cycling. Spectro-tomography, with its ability to resolve nanoscale elemental and chemical distributions in 3D, is an ideal tool to reveal redox heterogeneity and guide performance improvements.

**Fig. 4:**
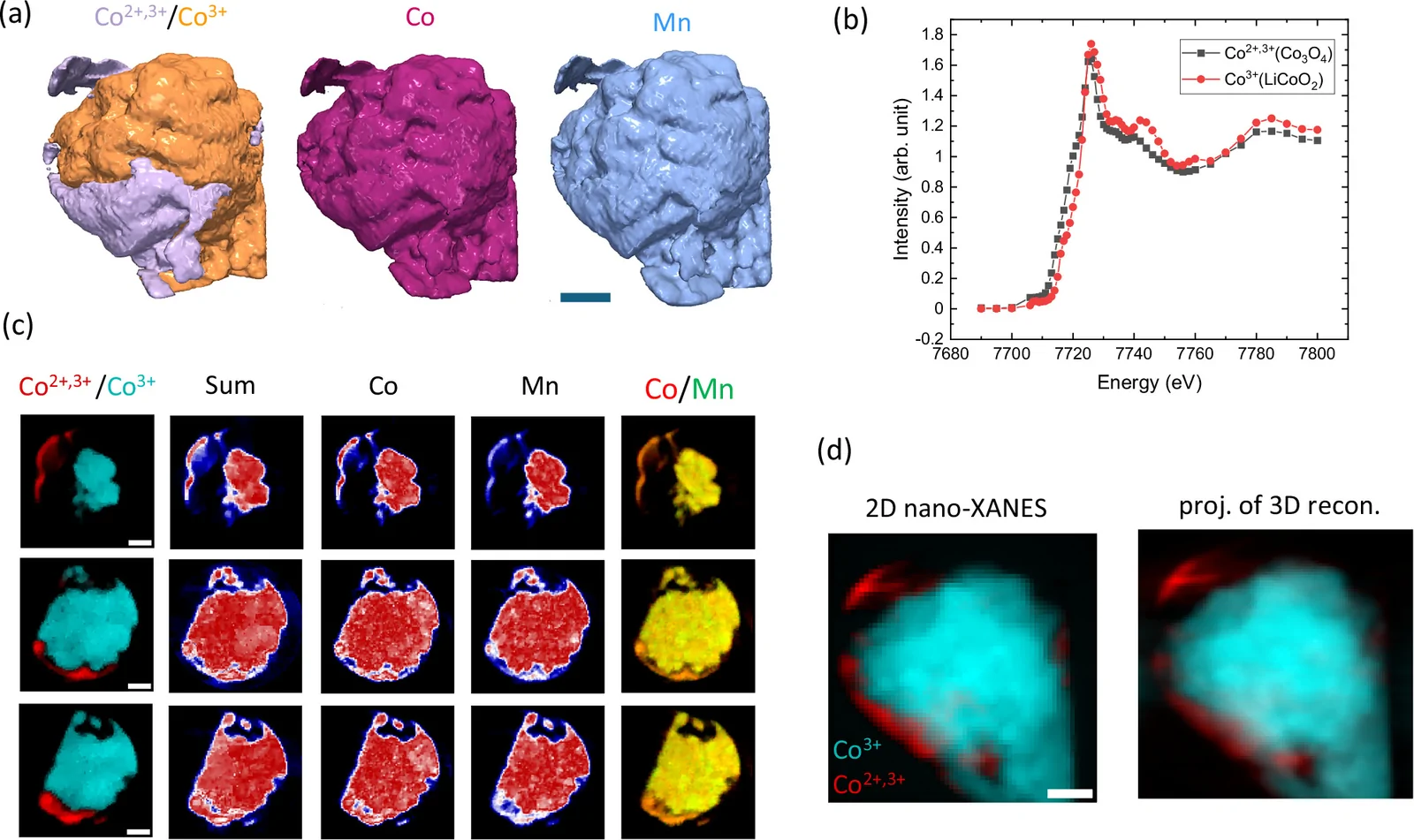
Spectro-tomography reconstruction of a cycled NMC811 particle. **a** 3D rendering showing the spatial distributions of two cobalt oxidation states—Co²⁺^,^³⁺ and Co³⁺—as well as elemental concentrations of Co and Mn, all derived from the same spectro-tomography dataset. **b** Reference XANES spectra for Co²⁺^,^ ³⁺ (Co₃O₄) and Co³⁺ (LiCoO₂). Dots and squares mark the energy points sampled during the measurement. **c** Cross-sectional views at three different locations. From left to right: distributions of Co oxidation states, their sum, elemental maps of Co and Mn, and a composite image highlighting chemical composition variation. **d** Projection of the 3D reconstruction of Co oxidation states compared with an independent 2D nano-XANES measurement acquired at the same angle. Scale bar in all images: 1 µm. Source data are provided as a Source Data file.

We performed the spectro-tomography across the Co’s K edge and obtained 3D reconstructions of the two Co oxidation states (Co^2+,3+^ and Co^3+^), along with Co and Mn distributions of a cycled NMC811 cathode particle. The results are depicted in Fig. 4a. In this experiment, 181 projection images over 180° with a 1° increment were collected. These images were acquired at 56 different energy points, randomly combined with the angle. The two reference spectra, along with the energy points sampled, are shown in Fig. 4b. Details about cycling conditions and experimental parameters are provided in the “Methods” section.

From the reconstruction, a new phase on the surface with reduced Co oxidation was clearly observed. This surface phase was known to form after cycling and adversely impact the performance of NMC battery^26,27^. The pristine particle did not exhibit this phase (see Fig. S13), confirming that it was introduced by cycling. Three cross-sectional views of the reconstructed volumes are presented in Fig. 4c, comparing the two oxidation states of Co and the concentrations of Co and Mn side-by-side. Some granular structures were seen in the composite map, suggesting the formation of boundaries of primary particles. Within the surface phase, a lower concentration of Mn and Co was observed. Their losses indicate that the particle suffered from transition metal dissolution, disrupting the original stoichiometry of NMC811 and driving the surface reconstruction and formation of the reduced oxidation state. The micro-cracking between the surface phase and the main body suggests that it may eventually be detached from the particle. To verify the fidelity of the 3D reconstruction, an independent 2D nano-XANES measurement was also conducted and compared with the projected image of the 3D reconstruction at the same angle, as shown in Fig. 4d. An agreement is evident.

**Fig. 5:**
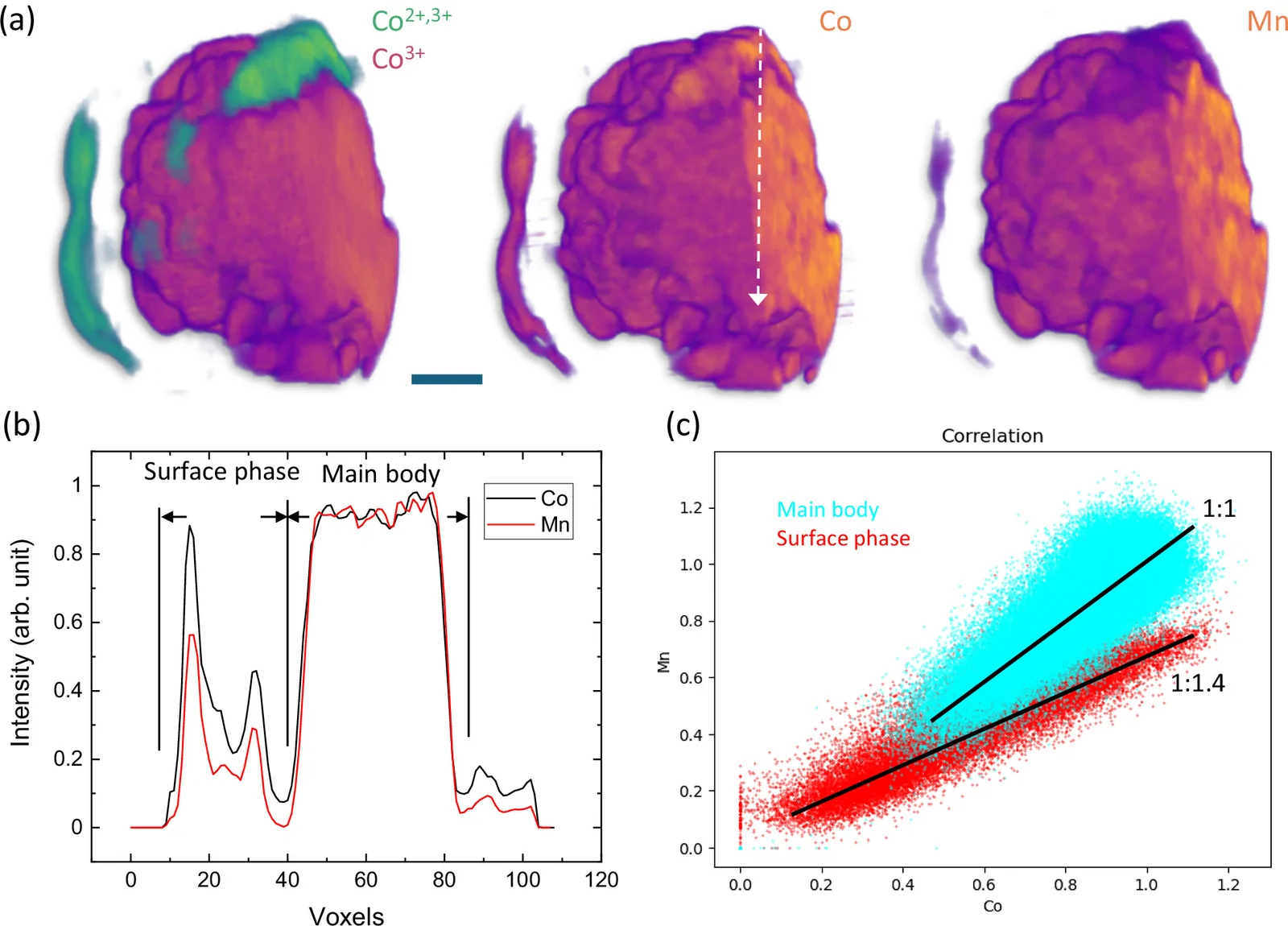
Variation in the Co:Mn ratio between two phases. **a** 3D rendering of Co oxidation states, as well as Co and Mn concentrations, clipped to reveal internal variations. **b** Line profiles of CO and Mn concentration along the dashed white line in (**a**). Both elements show reduced concentrations in the surface phase, with Mn exhibiting more significant depletion. **c** Correlation map between Mn and Co concentrations at each voxel. The two colors represent regions with different Co species. Linear correlations are observed in both regions, with Co:Mn ratio increased to 1.4:1 in the surface phase. Scale bar is 1 µm. Source data are provided as a Source Data file.

A quantitative analysis was performed to provide more insights. The high sensitivity of X-ray detection to transition metals, as compared with that of electrons, makes the quantitative analysis more accurate. Figure 5a presents clipped views of the 3D reconstructions, highlighting the internal variations of each measured quantity. In Fig. 5b, line profiles of Co and Mn concentrations across voxels within the particle are compared. Figure 5c displays a correlation map illustrating their concentrations at each voxel, where distinct linear correlations are evident in each phase. Inside the particle’s main body, the Co:Mn ratio remained 1:1 on average, consistent with that of the pristine particle (Fig. S10). In the surface phase, the Co concentration decreased to ~72% of that in the main body, while Mn’s concentration decreased even more significantly, to 50%. This resulted in a new phase with a Co:Mn ratio of 1.4:1. These observations align with previous studies suggesting the dissolution of both Co and Mn in the electrolyte, with Mn loss being severe^28^. The inactive surface phase accounted for ~10% of the total volume and was known to be a contributor to capacity fading. An in-depth discussion of the degradation mechanisms of NMC811 is beyond the scope of this work.

One shall note that because the beam energy was below Ni’s K-edge and its L-line fluorescence was too low to be detected by our detector, Ni concentration was not obtained simultaneously from the same measurement. A separate measurement was conducted at 10 keV, where Ni concentration was studied as well (Fig. S14). There was no obvious decrease in Ni concentration in the surface phase.

## Discussion

The high sensitivity and multi-modality capabilities of this technique make it a unique quantitative tool for 3D characterization for battery research, providing insights into the physicochemical changes in cathodes due to electrochemical cycling and aiding in developing design principles for improved cycling stability. Compared to other methods, full-field X-ray imaging lacks the sensitivity to detect the small surface phase with the absorption contrast. Transmission electron microscopy (TEM), while capable of providing atomic sensitivity, is restricted to surface area; it is not capable of unveiling variations of the entire volume of a micron-sized particle.

In cases where reference spectra are unavailable, ab initio reconstruction remains possible. The approach shifts from an analysis mode, which directly retrieves coefficients of known states, to a synthesis mode, which reconstructs the spectrum at each voxel. This mode requires a preliminary 2D nano-XANES measurement. Principal component analysis (PCA) or similar methods are then employed to decompose the collected spectra into a set of base components. After using the same methodology to calculate the coefficients of these components in 3D, the unknown spectrum at each voxel is reconstructed through superposition. In Fig. S15, we demonstrated how the synthesis mode works. At the current development stage, an independent 2D nano-XANES measurement is required as prior knowledge to determine the number of states. If neither the reference spectra nor the base components are available, the proposed method will not work, because the number of unknown spectral points cannot be reduced to the number of states.

Future development could follow several pathways. Because of the iterative nature of the method, the computation time can be up to a few minutes for a slice with a few hundred pixels. The main bottleneck is the linear solver used in ADMM. A significant speedup on GPUs can be achieved using gradient-based methods such as accelerated proximal gradient^29^ or automatic differentiation^30^. For large samples, one remaining challenge is the issue of self-absorption. While fluorescence modality offers high sensitivity, emitted fluorescence is absorbed within the sample before reaching the detector, which can be ignored only in small samples, like the ones in this study. Addressing this requires incorporating an additional matrix to account for self-absorption^31^, though this increases computational complexity. Another challenge is determining the presented states directly from the data without prior knowledge. Optimizing the selection of sampled energy points could also help reduce uncertainties^32^, and the same holds for refining the randomization process. Furthermore, acquisition time could be decreased by sparse sampling in angular space as well, employing machine-learning-enabled smart regularizer^33^. With these further developments, this high-sensitivity spectro-tomography technique could enable in situ and operando investigations of complex physicochemical processes at the nanoscale, which are critical in a wide range of scientific and technological fields, including energy storage materials, corrosion, and catalysis.

## Methods

### Physical model

Here, we consider a two-dimensional (2D) case for simplicity. For a 2D object with M pixels, we use an M×1 column vector, **x**, to represent it. Each projection results in a line on a detector. If we concatenate all measured data into a single column vector, **b**, it has a dimension of N×1, where N is equal to the number of detector pixels multiplied by the number of projections. An N×M radon transform matrix, **A**, maps the object from the image domains into the detector domain, based on the known projection angles. For regular tomography, we seek a solution of **x** that satisfies, ${{\bf{Ax}}}={{\bf{b}}}$.

When there is more than one chemical species present in the sample, their energy dependence across the absorption edge is required to distinguish them. The collected signal, either through fluorescence or absorption channel, is a linear sum of contributions from all k species. By noting that the signal variation with energy of a species is deterministic, that is, they cannot vary freely from point to point, we can encode this prior knowledge into the physical model by introducing another diagonal N×N matrix, **W**. For the *j*th species with concentration **x**_*j*_, its signal dependence on energy is described by **W**_*j*_, with its diagonal elements taken from the corresponding reference spectrum. In addition to the angle, we also need to specify the energy for each projection. As a result, we can model a spectro-tomography problem as, ${\sum }_{j}{{{\bf{W}}}}_{j}{{\bf{A}}}{{{\bf{x}}}}_{j}={{\bf{b}}}$.

### Solver based on the alternating direction method of multipliers

Turning a linear equation into an optimization problem is a standard technique to make the solution-seeking more trackable. We define a cost function,

$$L=\frac{1}{2}{\Vert \left({\sum }_{j}{{{\bf{W}}}}_{j}{{\bf{A}}}{{{\bf{x}}}}_{j}\right)-{{\bf{b}}}\Vert }_{2}^{2}+\mu {{\sum }_{j}\Vert {{{\bf{x}}}}_{j}\Vert }_{{{\rm{TV}}}},$$

The first term corresponds to a Gaussian likelihood function, which assumes white noise in the data. A total variation term, which is defined as the L_1_-norm of the derivatives, is added to regularize the solution. We adopt the Alternating Direction Method of Multipliers to find a solution that minimizes the cost function due to the method’s robustness and implementation simplicity^21^. First, we turn it into a constrained optimization problem by splitting the variable

$$\mathop{{\rm{argmin}}}\limits_{{{\bf{x}}}_{j},{{\bf{z}}}_{j}}\frac{1}{2}{\Vert ({\sum }_{j}{{\bf{W}}}_{j}{\bf{A}}{{\bf{x}}}_{j})-{\bf{b}}\Vert }_{2}^{2}+\mu {{\sum }_{j}\Vert {{\bf{z}}}_{j}\Vert }_{1},$$

Subject to: $${{\bf{D}}}{{{\bf{x}}}}_{j}={{{\bf{z}}}}_{j}$$

Here, **D** is a derivative matrix and has two components corresponding to two gradient directions. In ADMM, an argumented Lagrangian is used for enhanced robustness

$${L}_{{\rm {A}}}=\frac{1}{2}{\Vert ({\sum }_{j}{{\bf{W}}}_{j}{\bf{A}}{{\bf{x}}}_{j})-{\bf{b}}\Vert }_{2}^{2}+\sum _{j}{{\bf{u}}}_{j}^{{\rm{T}}}({\bf{D}}{{\bf{x}}}_{j}-{{\bf{z}}}_{j})+{\mu }_{1}{{\sum }_{j}\Vert {{\bf{z}}}_{j}\Vert }_{1}+\frac{{\mu }_{2}}{2}{{\sum }_{j}\Vert {\bf{D}}{{\bf{x}}}_{j}-{{\bf{z}}}_{j}\Vert }_{2}^{2}.$$

The superscript T corresponds to the transpose operator. This function can be minimized by solving sub-optimization problems over **x**_*j*_ and **z**_*j*_ alternatingly, followed by the dual update of the dual variable, ${{{\bf{u}}}}_{j}$. At the *i*th iteration, minimization over **x**_*j*_ leads to a linear equation

$$\left(\begin{array}{ccc}{{{\bf{A}}}}^{{{\rm{T}}}}{{{\bf{W}}}}_{1}^{{{\rm{T}}}}{{{\bf{W}}}}_{1}{{\bf{A}}}+{\mu }_{2}{{{\bf{D}}}}^{{{\rm{T}}}}{{\bf{D}}} & \cdots & {{{\bf{A}}}}^{{{\rm{T}}}}{{{\bf{W}}}}_{1}^{{{\rm{T}}}}{{{\bf{W}}}}_{k}{{\bf{A}}}\\ \vdots & \ddots & \vdots \\ {{{\bf{A}}}}^{{{\rm{T}}}}{{{\bf{W}}}}_{k}^{{{\rm{T}}}}{{{\bf{W}}}}_{1}{{\bf{A}}} & \cdots & {{{\bf{A}}}}^{{{\rm{T}}}}{{{\bf{W}}}}_{k}^{{{\rm{T}}}}{{{\bf{W}}}}_{k}{{\bf{A}}}+{\mu }_{2}{{{\bf{D}}}}^{{{\rm{T}}}}{{\bf{D}}}\end{array}\right)\times \left(\begin{array}{c}{{{\bf{x}}}}_{1}^{i}\\ \vdots \\ {{{\bf{x}}}}_{k}^{i}\end{array}\right)=\left(\begin{array}{c}{{{\bf{A}}}}^{{{\rm{T}}}}{{{\bf{W}}}}_{1}^{{{\rm{T}}}}{{\bf{b}}}-{{{\bf{D}}}}^{{{\rm{T}}}}{{{\bf{u}}}}_{1}^{i-1}+{\mu }_{2}{{{\bf{D}}}}^{{{\rm{T}}}}{{{\bf{z}}}}_{1}^{i-1}\\ \vdots \\ {{{\bf{A}}}}^{{{\rm{T}}}}{{{\bf{W}}}}_{k}^{{{\rm{T}}}}{{\bf{b}}}-{{{\bf{D}}}}^{{{\rm{T}}}}{{{\bf{u}}}}_{k}^{i-1}+{\mu }_{2}{{{\bf{D}}}}^{{{\rm{T}}}}{{{\bf{z}}}}_{k}^{i-1}\end{array}\right)$$

We can rewrite it in a more compact form

$${{{\bf{A}}}}^{{\prime} }{{{\bf{x}}}}^{{\prime} }={{\bf{b}}}^{{\prime} }.$$

This linear equation can be solved by many standard solvers. We used the GMRES function in scipy^34^ to update ${{{\bf{x}}}}_{j}$. Similarly, minimization over ${{{\bf{z}}}}_{j}$ results in a soft thresholding function

$${{{\bf{z}}}}_{j}^{i}=\,{\max }(|{{{\bf{v}}}}_{j}^{i}|-\frac{{\mu }_{1}}{{\mu }_{2}},0)\times {{\rm{sign}}}({{{\bf{v}}}}_{j}^{i}),{{{\bf{v}}}}_{j}^{i}={{\bf{D}}}{{{\bf{x}}}}_{j}^{i}+\frac{1}{{\mu }_{2}}{{{\bf{u}}}}_{j}^{i-1}.$$

Note here that an anisotropic form of TV is employed. For isotropic TV, the solution will be slightly different^35^. The last step is the dual-update

$${{{\bf{u}}}}_{j}^{i}={{{\bf{u}}}}_{j}^{i-1}+{\mu }_{2}({{\bf{D}}}{{{\bf{x}}}}_{j}^{i}-{{{\bf{z}}}}_{j}^{i})$$

These update steps are repeated until either a maximum number of iterations or convergence tolerance is reached. The complete implementation of the algorithm can be found in ref. ^36^.

### Test sample preparation

An aqueous solution of Hematite and Steel nanoparticles (purchased from US Research Nanomaterials Inc.) was made at ~1:1 ratio by weight in water. 10 ml of the solution was aliquoted in a PCR tube, to which was added 10 ml of APTES and 10 µl of TEOS to form a sol-gel solution with the iron nanoparticles. The sample was placed on a shaker at 700 RPM for 2 h, followed by drop-casting onto silicon chips and dried in a vacuum oven at 100 °C for 3 days. The sample was inspected via SEM-EDS and mounted to a Tungsten needle using a dual-beam FIB. See supplementary materials for more details.

### NMC811 sample preparation

LiNi_0.8_Co_0.1_Mn_0.1_O_2_ (NMC811) material was cycled in Li-metal batteries with Li metal anode and 1M Li bis(fluorosulfonyl)imide (LiFSI) in 1,2-dimethoxyethane with 3 wt% CsNO_3_ additive as the electrolyte^37^. The NMC811 loading was 5 mAh/cm^2^ (NMC811:polyvinylidene difluoride:carbon black ratio of 96:2:2), and the Li metal anode thickness was 50 µm. Cycling was performed at C/3 rate for 100 cycles within the voltage window of 2.8–4.3 V. The cell after cycling was disassembled for harvesting the NMC811 cathode inside an argon-filled glovebox. The cycled cathode was washed with DME to remove residual electrolyte and left in the glovebox to dry for an hour. The dried cathode particle was mounted to a sharp pin using FIB and then delivered to the beamline for analysis. See supplementary materials for more details.

### X-ray measurement

Spectro-tomography measurement was conducted at the hard X-ray nanoprobe beamline (HXN) of National Synchrotron Light Source II (NSLS-II), Brookhaven National Laboratory. A monochromatic beam was selected by a Si (111) double-crystal monochromator and then focused to a size of around 40 nm by a Fresnel Zone Plate with a diameter of 250 µm and an outermost zone width of 30 nm (Applied Nanotools). The nanobeam raster-scanned the sample under investigation, while an energy-dispersive detector (Vortex ME-3, Hitachi) collected all fluorescence photons. The obtained fluorescence spectra were fitted using PyXRF^38^ to remove background and separate overlap peaks to yield quantitative maps of individual elements. Two-dimensional fluorescence images were acquired at projection angles spanning 180° with a 1° incremental step in all cases. At each projection angle, the monochromatic beam energy was tuned based on the codebook, which randomly selected energy sampling points across the absorption edge.

For the test object, a grid scan of 100 × 100, a step size of 50 nm, a dwell time of 20 ms, and 52 energy points were used to acquire the spectro-tomography dataset. For the NMC811 particle, a grid scan of 100 × 100, a step size of 60 nm, and a dwell time of 20 ms was used to obtain the projection image. A total of 181 projections with 1° increments, conducted at 56 energy points randomly combined with the angle, were acquired to generate the spectro-tomography dataset. This led to a total acquisition time of about 10 hours for a single sinogram. In comparison, it would exceed 500 hours to acquire every sinogram at each energy for the conventional spectro-tomography.

Projection images were aligned using StagReg^39^ and normalized to a fluorescence signal that remained constant across the energy range (Cr for the test sample and Mn for the NMC811 sample, respectively). Reference spectra were obtained from bulk measurements of the same chemical species.

Two-dimensional nano-XANES measurement was conducted at HXN as well, with the difference that the imaging was performed at all 56 energy points without changing the projection angle. See ref. ^2^ for more details of this technique.

## Supplementary information

Supplementary Information

Description of Additional Supplementary Files

Supplementary Movie 1

Supplementary Movie 2

Transparent Peer Review file

## Source data

Source Data

## Data Availability

Source data are provided with this paper. All experimental data presented in this study have been deposited in the Zenodo database to download https://doi.org/10.5281/zenodo.21792166 Source data are provided with this paper.

## Code availability

Reconstruction code can be downloaded at https://github.com/hanfeiyan/Spectro_tomography
