# Supplementary material for: Sparse X-ray spectro-tomography for high-sensitivity three-dimensional chemical imaging at the nanoscale
Source: Nat Commun. 2026 Sep 16;17:10867. doi: 10.1038/s41467-026-77821-5 (PMC13672795; doi:10.1038/s41467-026-77821-5)
Supplement: Supplementary file 1 [file 41467_2026_77821_MOESM1_ESM.pdf]

Sparse X-ray Spectro-Tomography for High-Sensitivity Three-Dimensional Chemical Imaging at the Nanoscale

Hanfei Yan[1*], Ajith Pattammattel[1], Aaron Michelson[2], Enyuan Hu[3] and Kangxuan Xia[3]

[1]National Synchrotron Light Source II, Brookhaven National Laboratory, Upton, NY 11973, USA

[2]Center for Functional Nanomaterials, Brookhaven National Laboratory, Upton, NY 11973, USA

[3]Chemistry Department, Brookhaven National Laboratory, Upton, NY 11973, USA

*Corresponding author. Email: hyan@bnl.gov

Theoretical investigation

In this section, we present reconstruction results under various conditions using simulated data.

Impact of total variation regularization

Due to the increased number of unknown variables, minimizing only the fidelity term can result in significant reconstruction artifacts. This is similar to conventional tomography cases where the dataset is incomplete due to sparse views or limited angular coverage. In Fig. S1, we present results obtained from different linear solvers with and without total variation (TV) regularization. As shown, although the stochastic sampling scheme produces the best outcome with the fewest artifacts, the result remains unsatisfactory without TV. This highlights the important role of TV in suppressing background fluctuations, eliminating crosstalk, and enhancing contrast. Also, the selection of linear solvers is important, since they impose different constraints implicitly on the problem. In Fig. S2, we illustrate the progressive improvement of the reconstruction over iterations. The algorithm converges rapidly, with most of the improvement occurring within the first 10 iterations. Therefore, stochastic energy sampling, joint reconstruction, and TV regularization constitute the three fundamental pillars of the proposed method.

Impact of hyperparameters and number of states

The ADMM algorithm involves two tuning hyperparameters, μ_1 and μ_2. The first, μ_1, controls the weight of the regularization term. For datasets with a high signal-to-noise ratio, a smaller value is recommended, while noisier datasets require a larger one. The second hyperparameter, μ_2, influences the convergence speed of the algorithm. Similarly, a smaller value is preferable for high-quality data. Number of states used for the fitting is also important. When incorrect number of states is used, it leads to a bigger error. In principle, we can develop a strategy that tests all possible combinations of states and selects the one that yields the smallest error—a data-driven selection process. However, due to imperfections in the data, multiple combinations can yield

similar mse and an incorrect overfitting may occur. Therefore, additional constraints—such as prior electrochemical knowledge to narrow the selection—are needed to determine the reference spectra. In Fig. S3, we present several examples illustrating the impact of these parameters. At current stage, proper tuning and the exact correct selection of reference spectra known to be present in the sample under investigation is essential for achieving optimal results, especially when the data is noisy.

Impact of noise

Experimental data inherently contains noise, making it essential to evaluate its impact on reconstruction quality. In Fig. S4, we simulate two cases with Poisson noise. The corresponding photon statistics for the sinogram used in the reconstruction are shown on the right. As expected, reconstruction quality degrades noticeably with increasing noise. Spectroscopic analysis generally requires a high signal-to-noise ratio for accurate spectral fitting, and the same holds true for spectro-tomography. In fact, the photon count requirements for spectro-tomography are higher than those for conventional tomography. To achieve reliable reconstruction, we recommend collecting at least a few hundred photons per pixel.

Impact of spectral randomness

We randomize the matrix $\boldsymbol{W}$ by performing stochastic sampling over the energy domain. For theoretical investigation, we generate a simulated dataset using two hypothetical spectra composed of random values in the range of (0,1), ensuring minimal correlation between them. A reconstruction comparison is shown in Fig. S5, where both hypothetical random spectra and real Fe spectra were used to generate the sinogram. The results clearly show that the random spectra yield better reconstruction quality. This also points to a potential direction for further improving the technique.

Impact of initial guess

The previous test case involved two spatially separated species. Here, we evaluate the algorithm under a more challenging condition, where two complex patterns exhibit significant spatial overlap. The "Flower" and "Lotus" images (128×128 pixels) were used to represent two Fe species (Fe^0 and Fe^{3+}), and 121 projections over 180° were generated for tomographic reconstruction. In Fig. S6, we present the reconstructed distributions of the two species. Three different initial guesses were tested—constant, random, and near ground truth. For stochastic sampling, all initializations led to similar results, successfully separating the two species. However, for interlaced and segmented sampling, only the initialization near the ground truth produced good reconstructions, free from strong artifacts and crosstalk. This suggests that interlaced and segmented sampling schemes are more susceptible to local minima; they converge to the correct solution only when the initial guess is reasonably close to the ground truth.

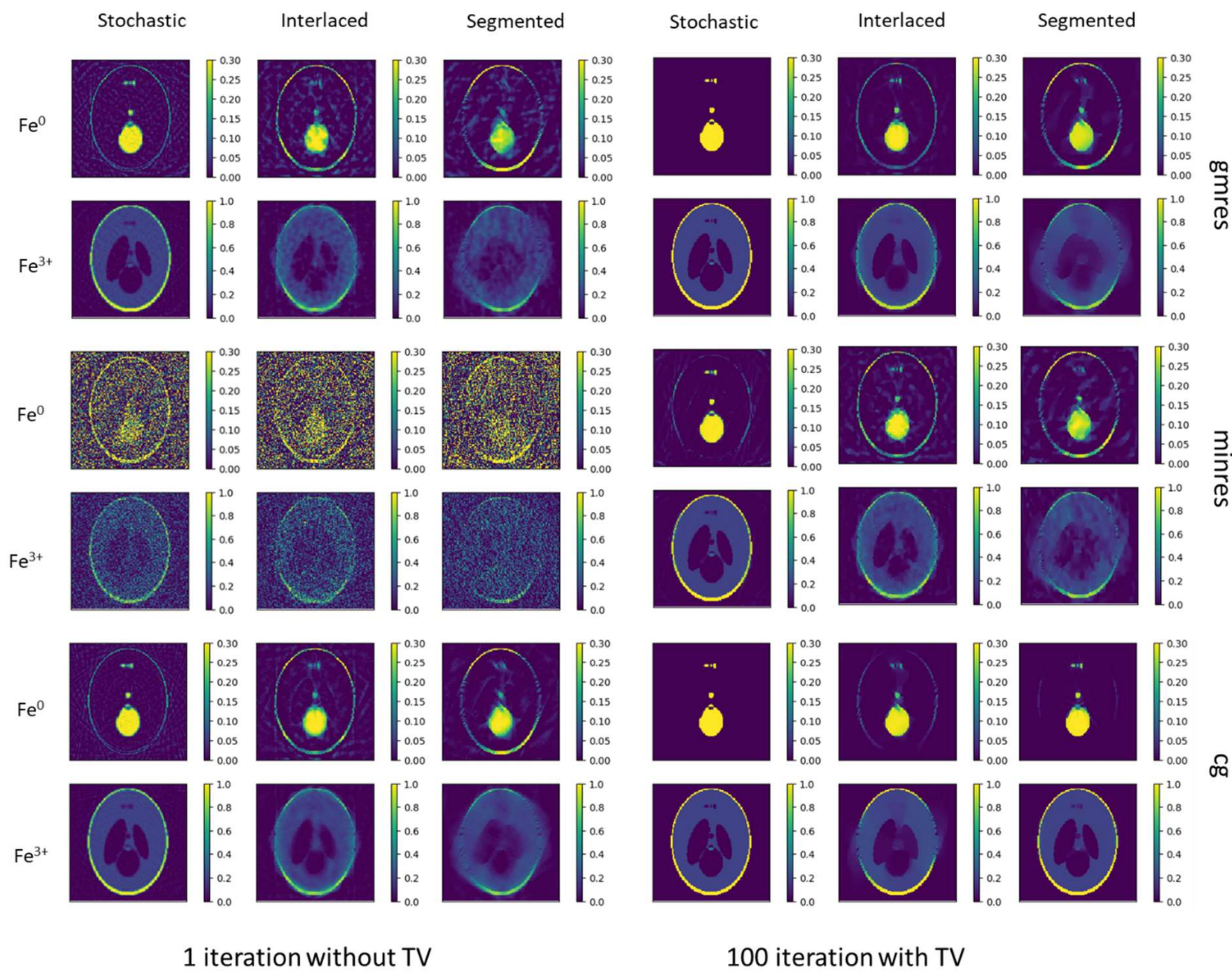

Fig. S1 **Results of solving Eq. (2) using different sampling schemes and linear solvers.** Without TV as the regularizer, all showed cross -talk and artefacts. With TV, all results are improved, but stochastic energy sampling yields the best result. The simulation conditions are identical to those in Fig. 2a.

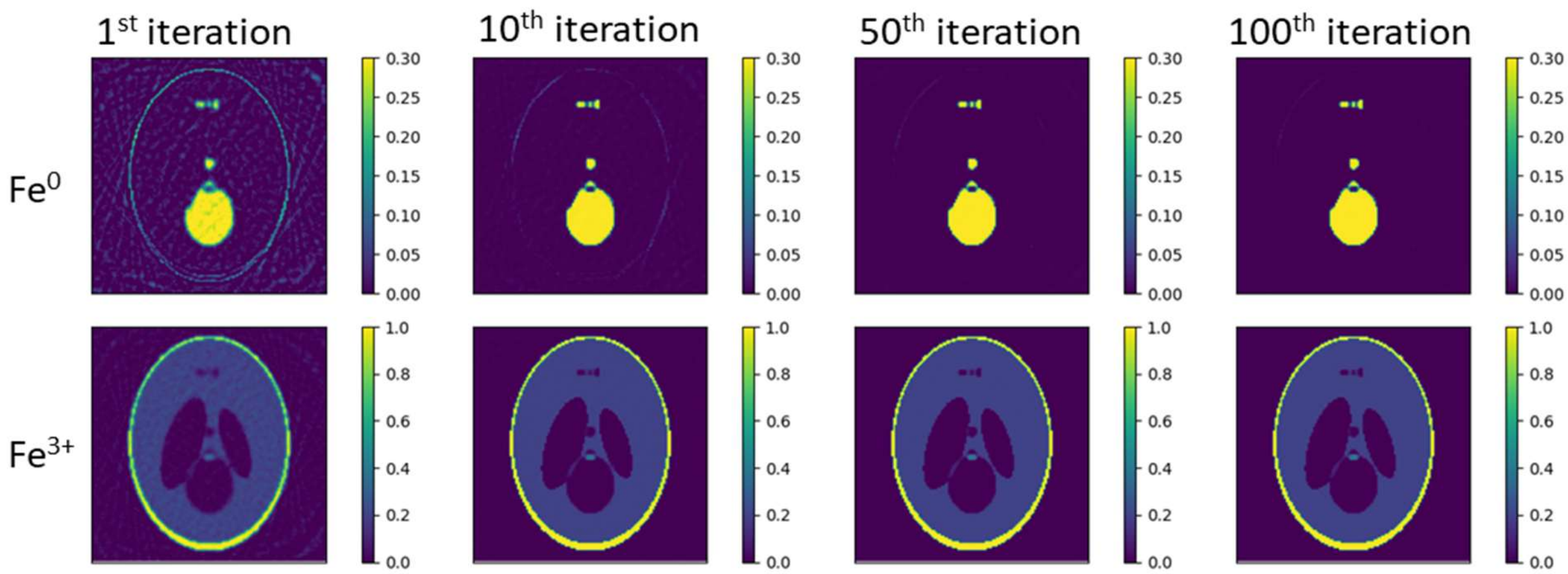

Fig. S2. **Progressive improvement over iterations with TV regularization.** Background fluctuations are significantly suppressed, and blurring is effectively eliminated.

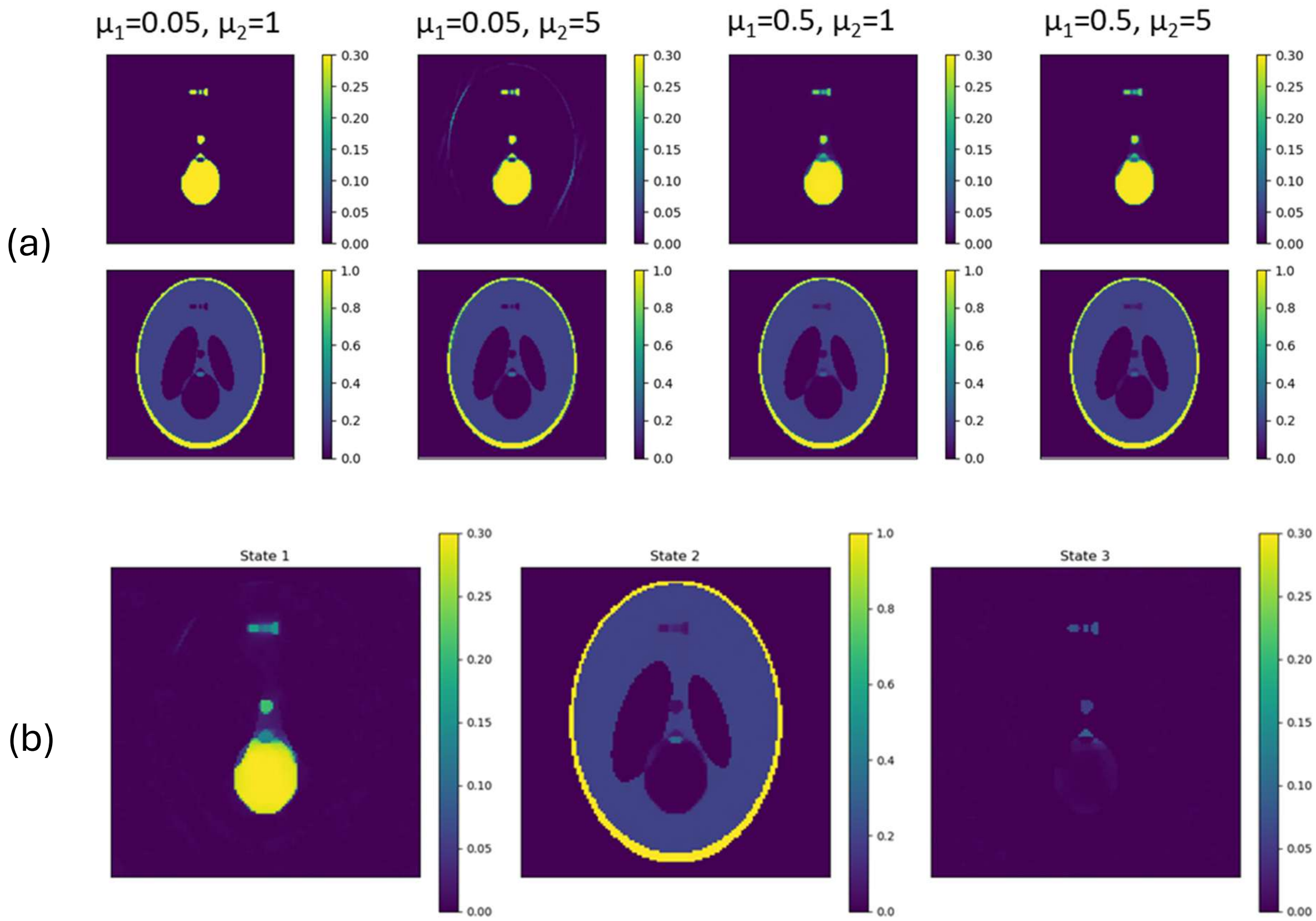

Fig. S3. **Impact of hyperparameters and numer of states on reconstruction.** (a) results with different μ_1 and μ_2 values. The conditions are the same with those in Fig. 2a. Subtle difference in reconstruction quality can be observed. (b) Spectro-tomography reconstruction with three states, while only two states exist. It leads to a bigger error as compared with the correct two-state reconstruction. Therefore, in principle we can use minimized mse to guide the selection of states – a data driven process. However, imperfection in the data may lead to an overfitting problem if no prior knowledge is employed to constrain the selection.

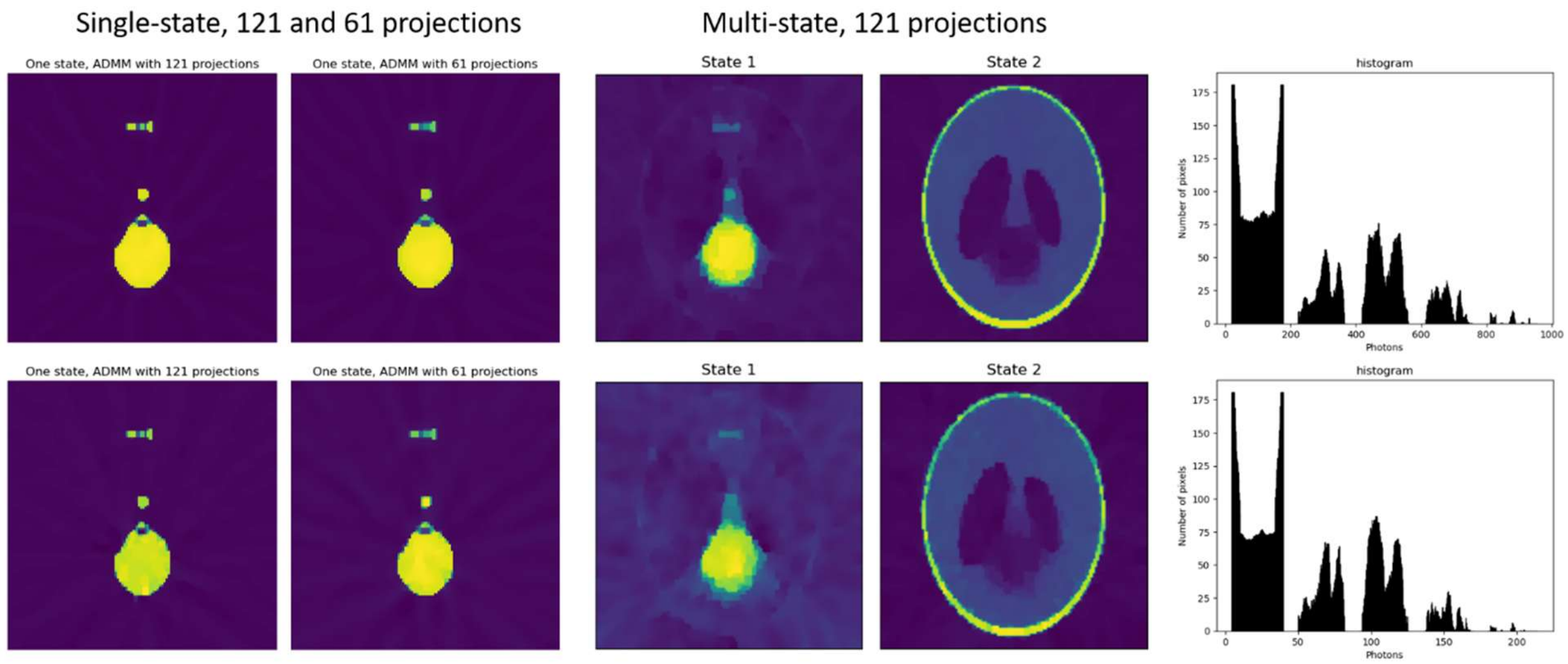

Fig. S4. **Impact of noise.** We reconstruct the two oxidation states of the phantom with simulated data that contains Poisson noise. The right-most figure shows the intensity histogram of the used sinogram. In comparison, we also did reconstruction with the same level of noise when there is only a single state. The same number of projections (121) and half of it (61) were tested. Reconstruction quality in single state (projections acquired at one energy) degrades slower than the multi-state.

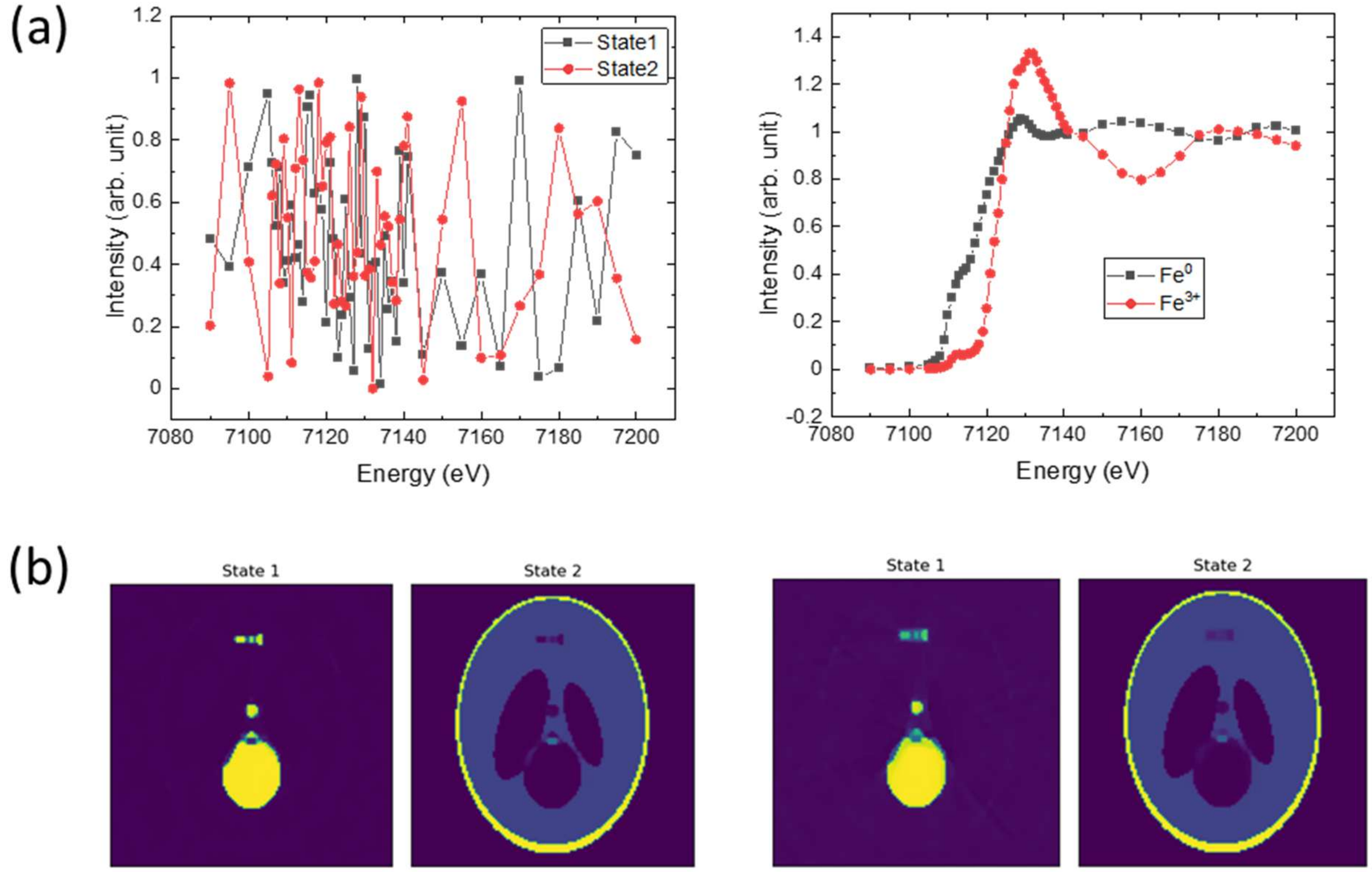

Fig. S5. **Impact of spectral randomness.** (a) Spectra used to generate the simulated data. (b) Spectro-tomographic reconstruction of the two states. The reconstruction using hypothetical random spectra yields better results.

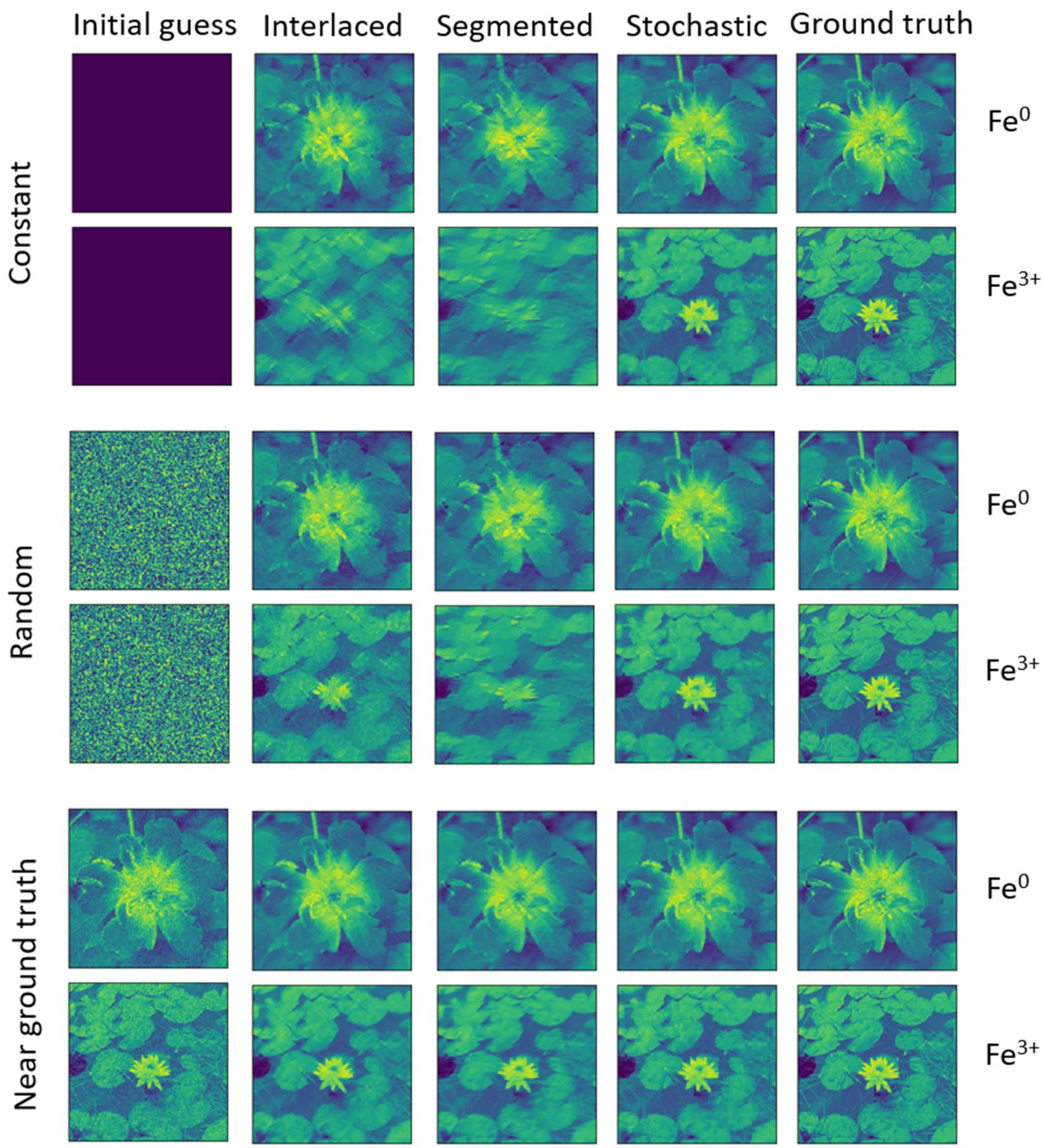

Fig. S6. **Simulated experiment with two Fe species (Fe^{0} and Fe^{3+}) strongly overlapping with each other.** Results from top to bottom correspond to three different initial guesses of the solution - constant, random, and near ground truth.

Sample preparation and SEM characterization

In this section we present more details about sample preparation and SEM characterizations as well.

FIB and EDS measurements

Both the stainless steel/hematite nanoparticle mixture and NMC811 particles were prepared using FIB to mount them to a sharp pin for spectro-tomography measurement. In Fig. S7-S11 we show their morphologies in SEM and compositions in EDS analysis.

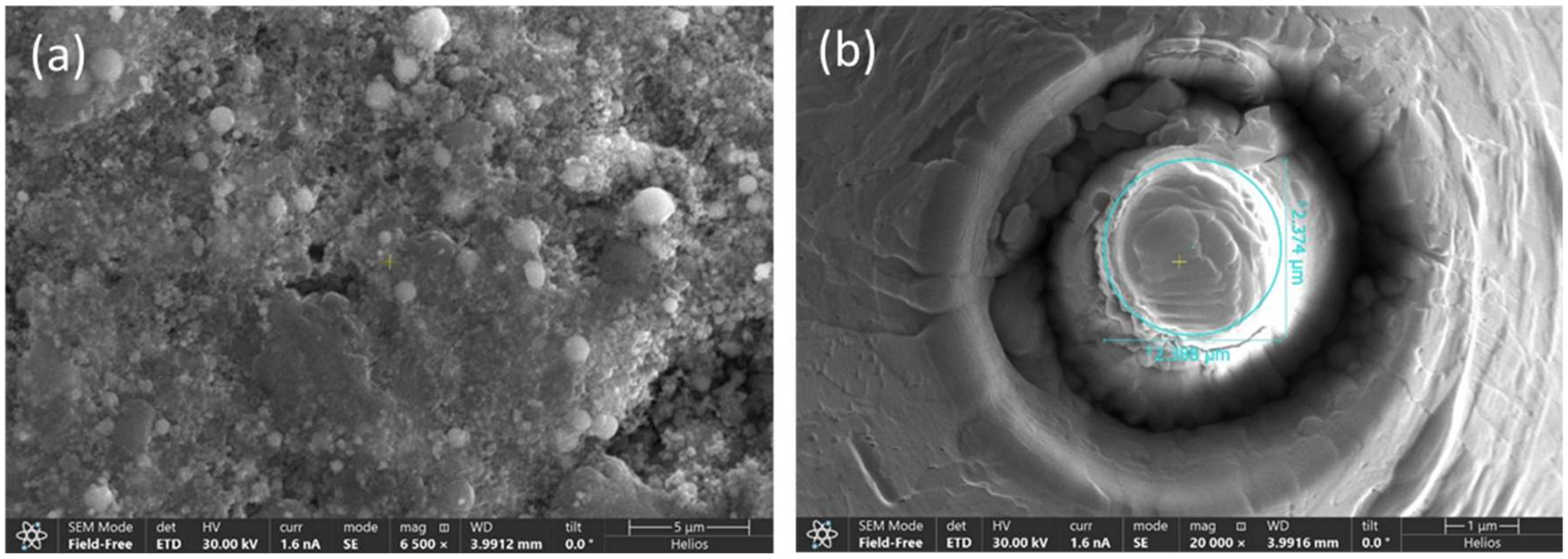

Fig. S7. **SEM of stainless steel-Hematite Silicate.** a) SEM micrograph of the silica encased iron nanoparticle matrix, at 30KeV steel particles stand in stark relief to the silica and hematite composite. b) Top-down view of cylindrical section of Steel/Hematite silicate with annotation showing 2.4 µm diameter.

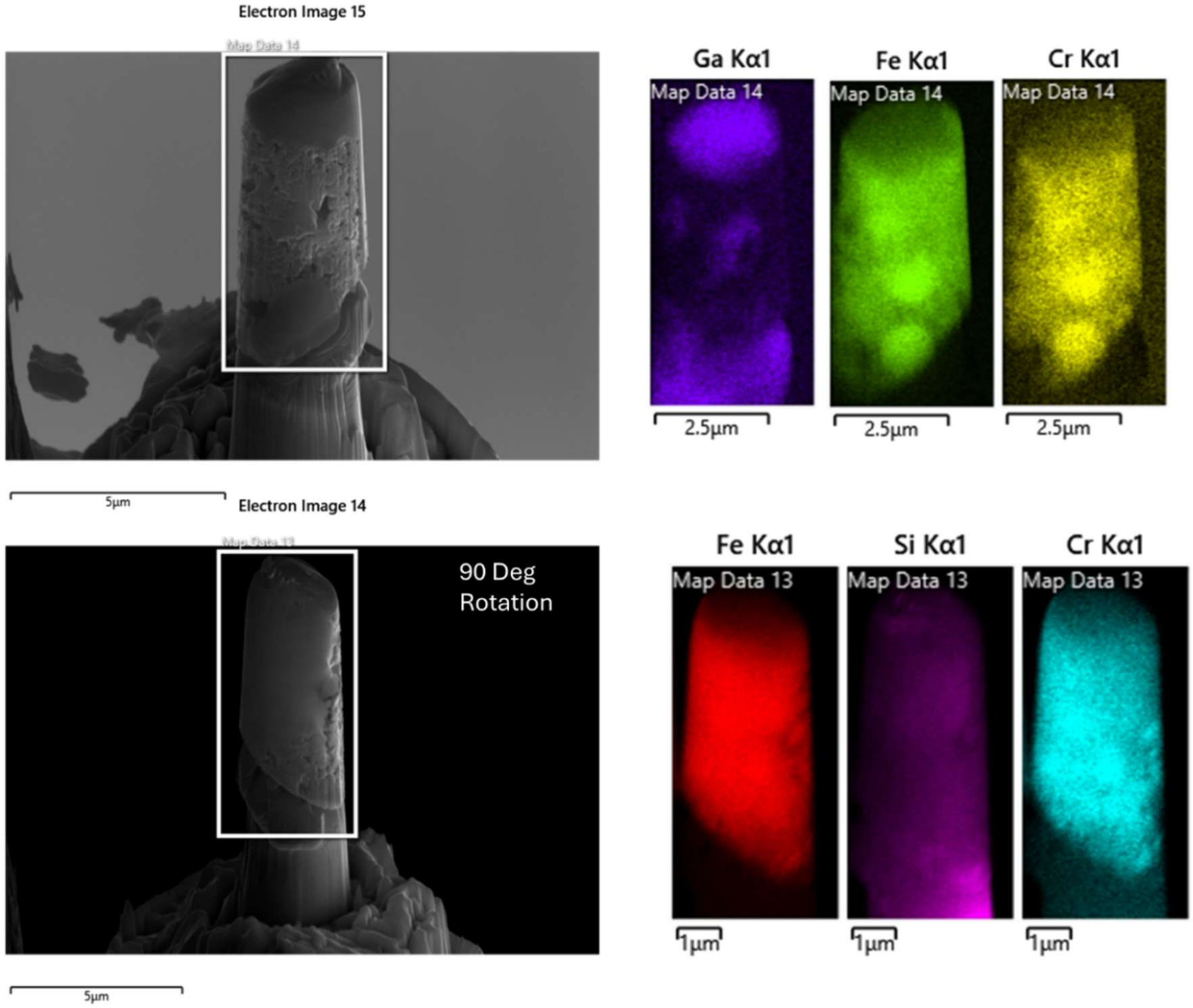

Fig. S8. **FIB/SEM Prepared Cylindrical Sample of stainless steel/hematite nanoparticle mixture.** A sample of 2.4 µm is shown at two angles with respect to the electron beam (0 and 90degrees relative). Zero degrees angle (Top) with EDS map which shows the carbon cap represented by Gallium, Iron, Chromium and silicon for the 90° setting (Bottom) shown.

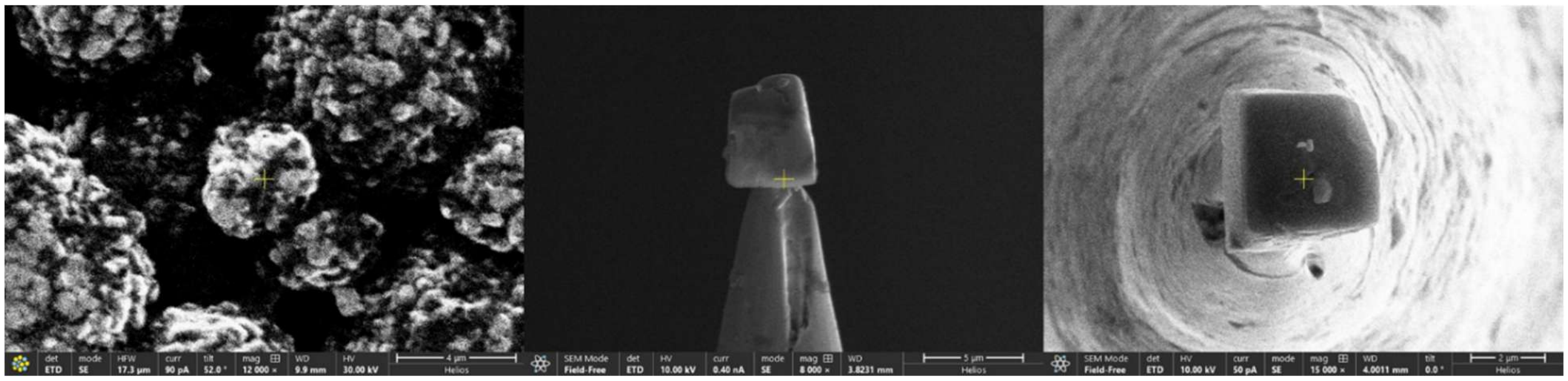

Fig. S9. **Cycled NMC Sample.** Left) Cycled NMC particle selected for size (<5um). The Sample is capped with carbon to protect the sample during mounting, the sample is then shown mounted to the tungsten needle from the side (Center) and top view (Right)

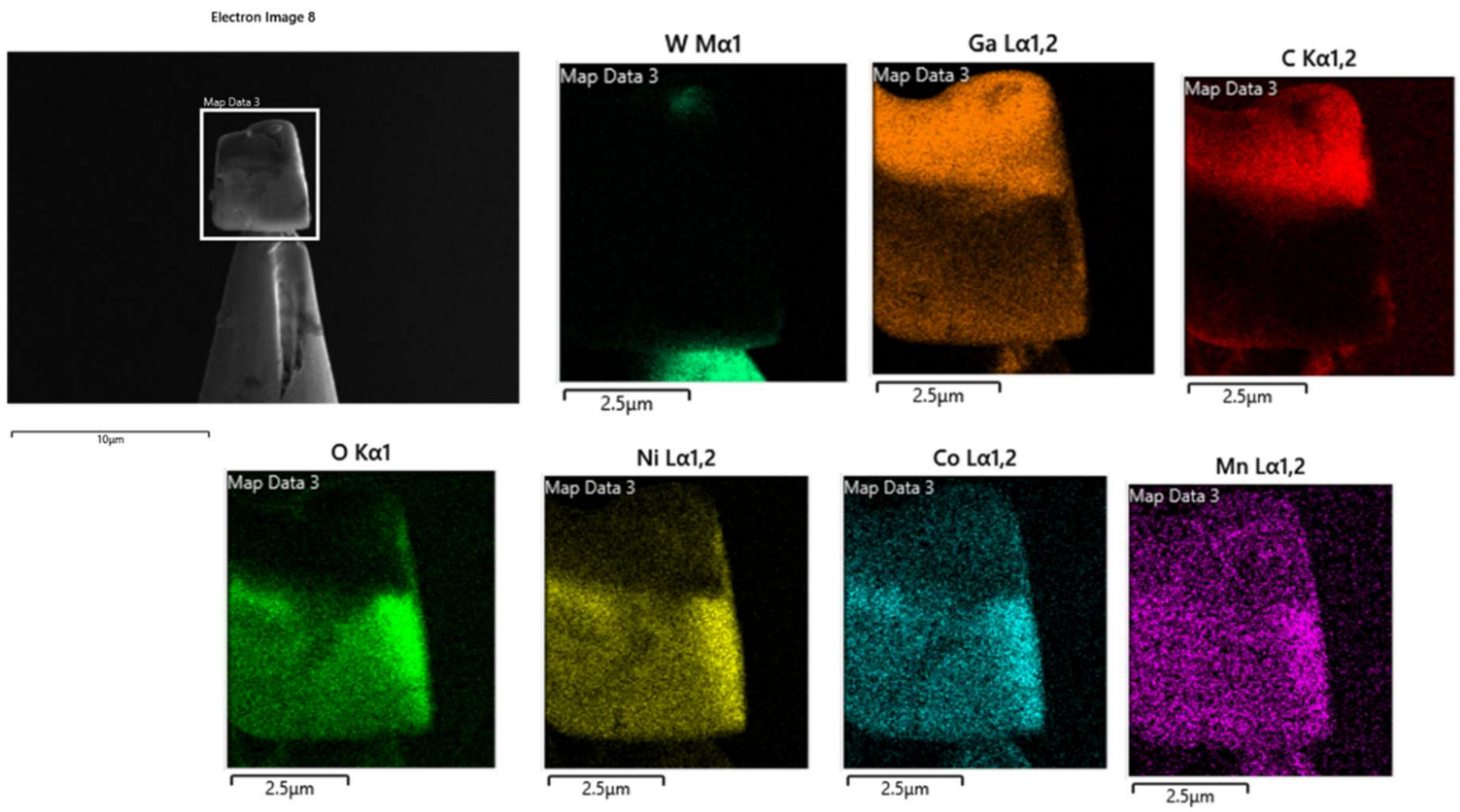

Fig. S10. **Cycled NMC SEM-EDS.** SEM EDS of the Cycled NMC sample from the side view, showing the carbon cap protecting the sample along with the dominant species, Ni, Manganese and Cobalt oxides.

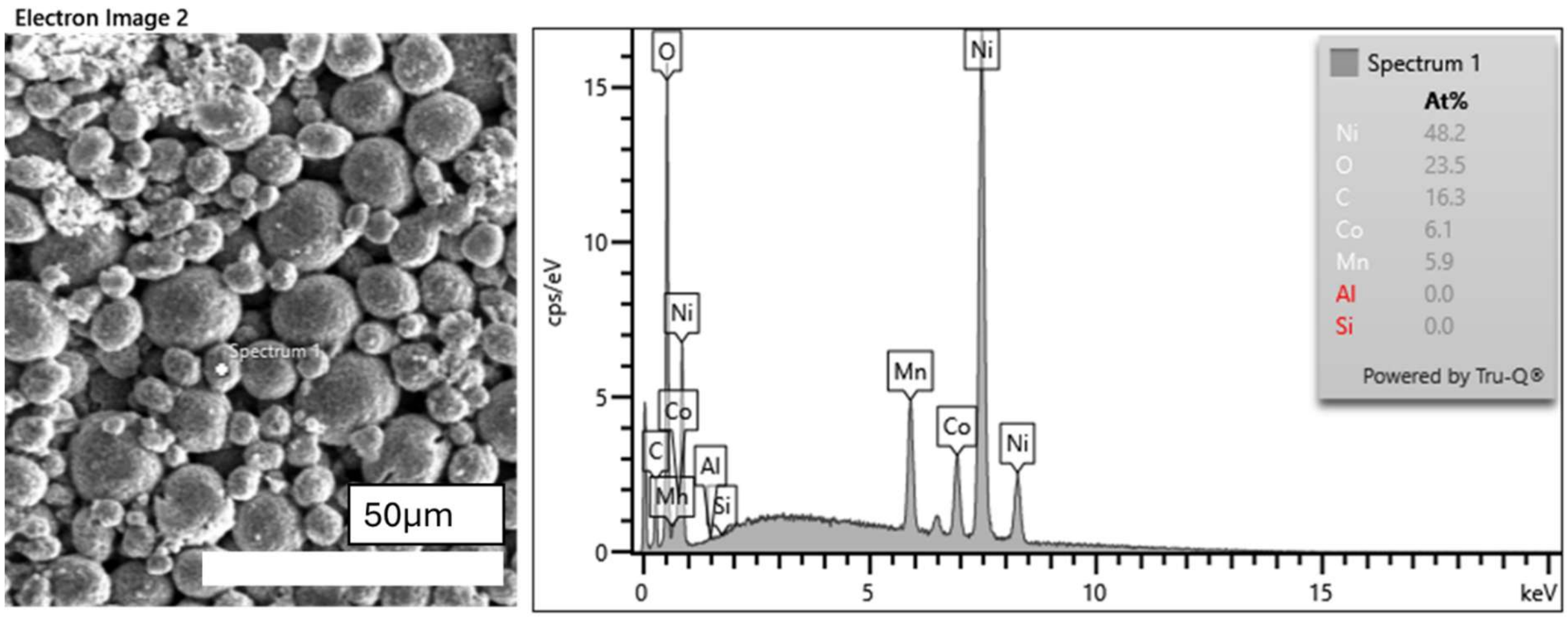

Fig. S11. **Pristine NMC SEM-EDS.** Left) SEM Image and spectrum spot marker Right) EDS spectrum with At% table with dominant species nickel, manganese, and cobalt identified. Ni:Mn:Co ratio agrees well with 8:1:1.

Additional experimental data

In this section, we present additional experimental data that supports the claims in the main text.

Spectro-tomography measurement on the second stainless steel/hematite nanoparticle mixture

A measurement on another test sample was performed to verify the observation of excessive Fe^0 on the boundary. Results are shown in Fig. S12. Like the case presented in Fig. 3, distributions of Fe^0 and Cr are highly correlated, but there is an excessive amount of Fe^0 on the boundary. This provides another verification of the observation. We also assessed resolution of reconstructions in Fig. 3C by comparing power density spectrum. Chemical state reconstruction showed a lower half-period resolution of 80 nm as compared to that of the fluorescence (50 nm).

2D nano-XANES of the pristine NMC811 particle

A 2D nano-XANES measurement on the pristine NMC811 sample was conducted to determine the chemical state of Co before cycling. From Fig. S13, it is evident that only Co^{3+} exists in the pristine particle.

Ni distribution of the cycled NMC811 particle

Since the Co K-edge lies below that of Ni, Ni's distribution could not be captured during the spectro-tomography measurement. Therefore, a separate tomography scan was performed at 10 keV to reveal the Ni distribution. In Fig. S14, we present 3D renderings of the reconstructions, with cutaway views to highlight internal variations. Corresponding cross-sectional slices and a composite map are shown at the bottom. The Ni distribution appears largely uniform, with no noticeable change in concentration at the surface phase.

Synthesis mode

When the reference spectra are unknown, the basis components can be extracted using PCA or non-negative decomposition from 2D nano-XANES data, though this requires additional measurement time. After performing spectro-tomography reconstruction with the learned basis components, the full spectrum can be recovered by combining these components. Figure S15 illustrates this procedure and compares the results with those obtained from direct spectro-tomography reconstruction.

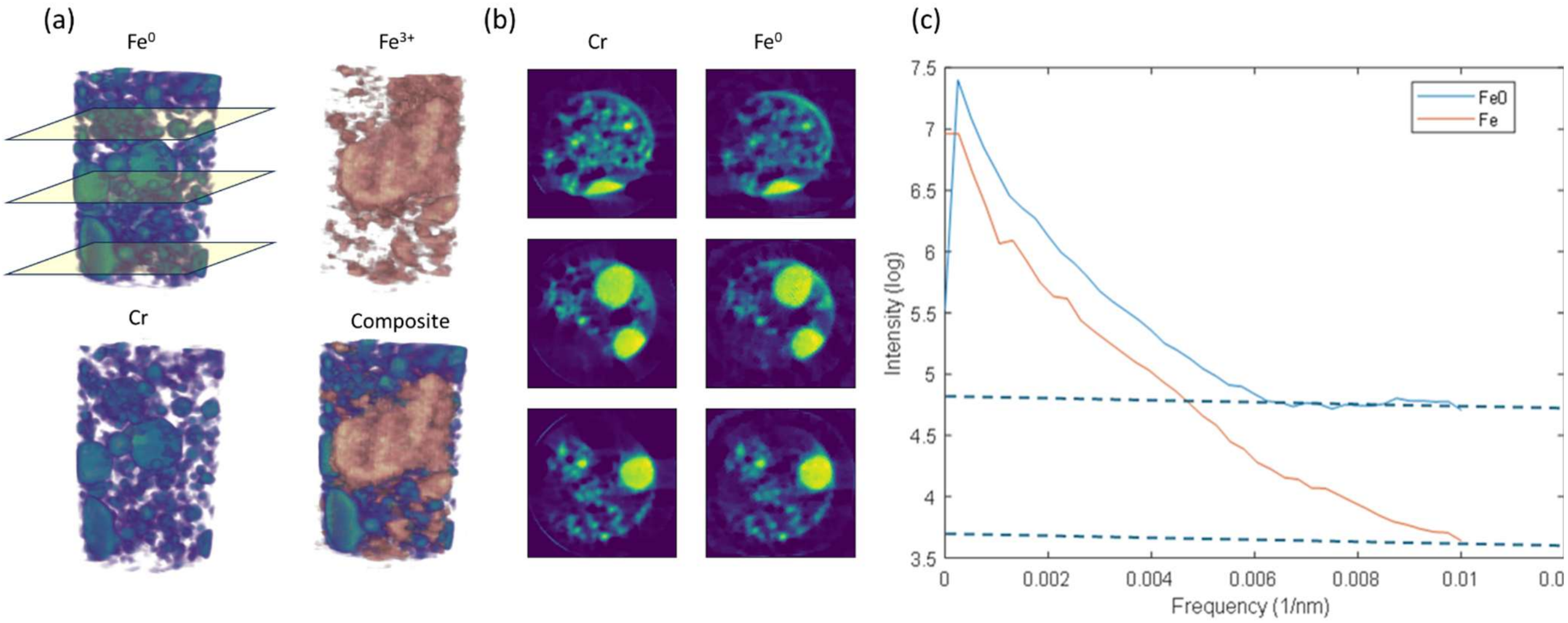

Fig. S12. **Spectro-tomography reconstruction of the second test sample.** (a) 3D renderings showing distributions of Fe^0, Fe^{3+}, Cr, and the composite of two iron oxidation states. (b) cross-sectional views of Cr and Fe^0 distributions at three heights shown in (a). (c) comparison of power spectrum density plot of the reconstructions of Fe^0 and Fe.

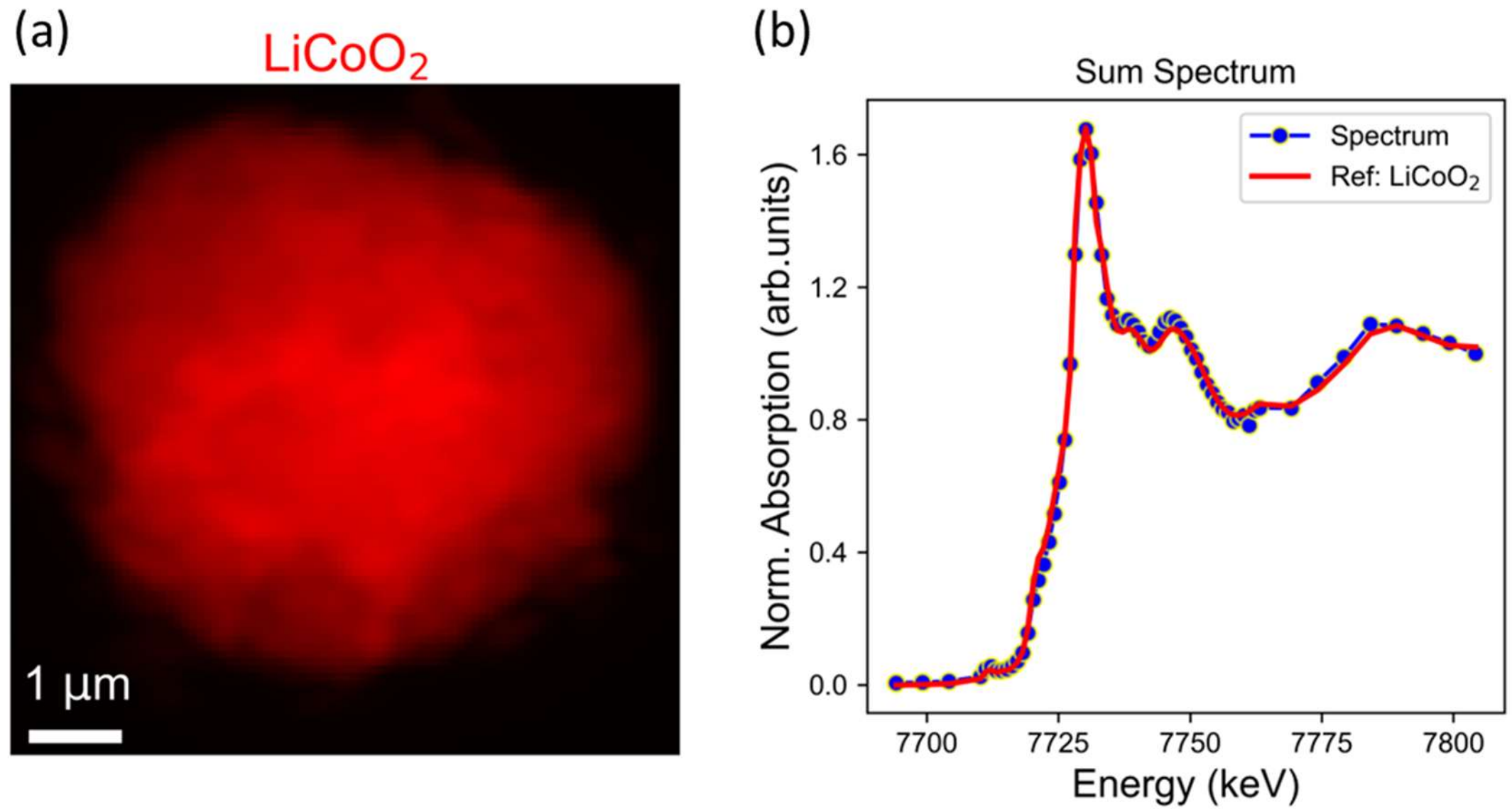

Fig. S13. **nano-XANES measurement of the pristine NMC811 cathode particle.** (a) Chemical state map of Co based on single-pixel spectrum fitting. Only Co^{3+} was detected throughout the entire particle. (b) summed spectrum from all pixels and the fitting. The experimental data can be well-fitted with the reference spectrum of $LiCoO_2$.

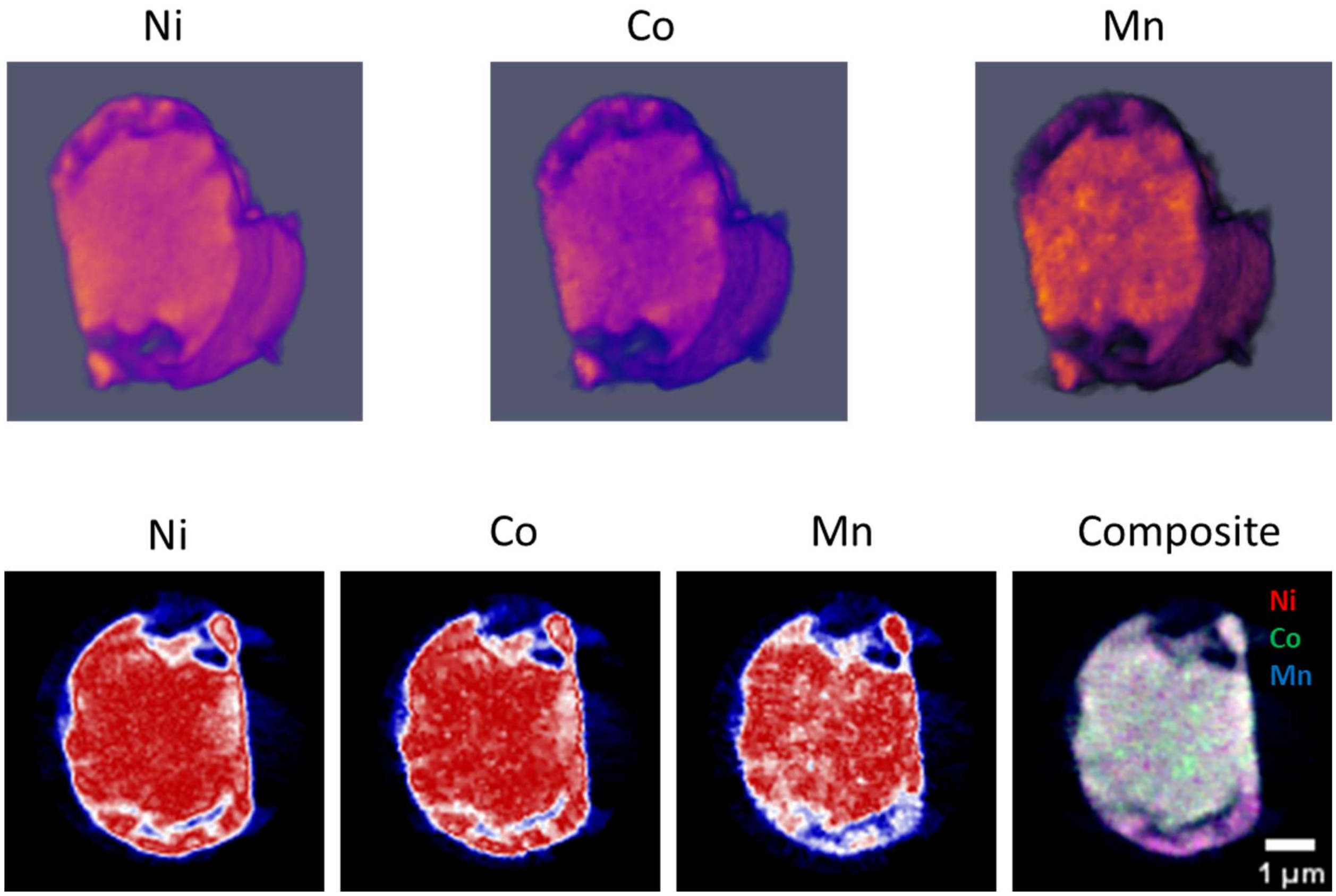

Fig. S14 **Tomography performed at 10 keV.** 3D renderings showing Ni, Co and Mn concentrations based on fluorescence data taken at 10 keV are presented. The particle is clipped to show the internal variations. Cross-sectional views correspond to the clipped plane are shown at the bottom.

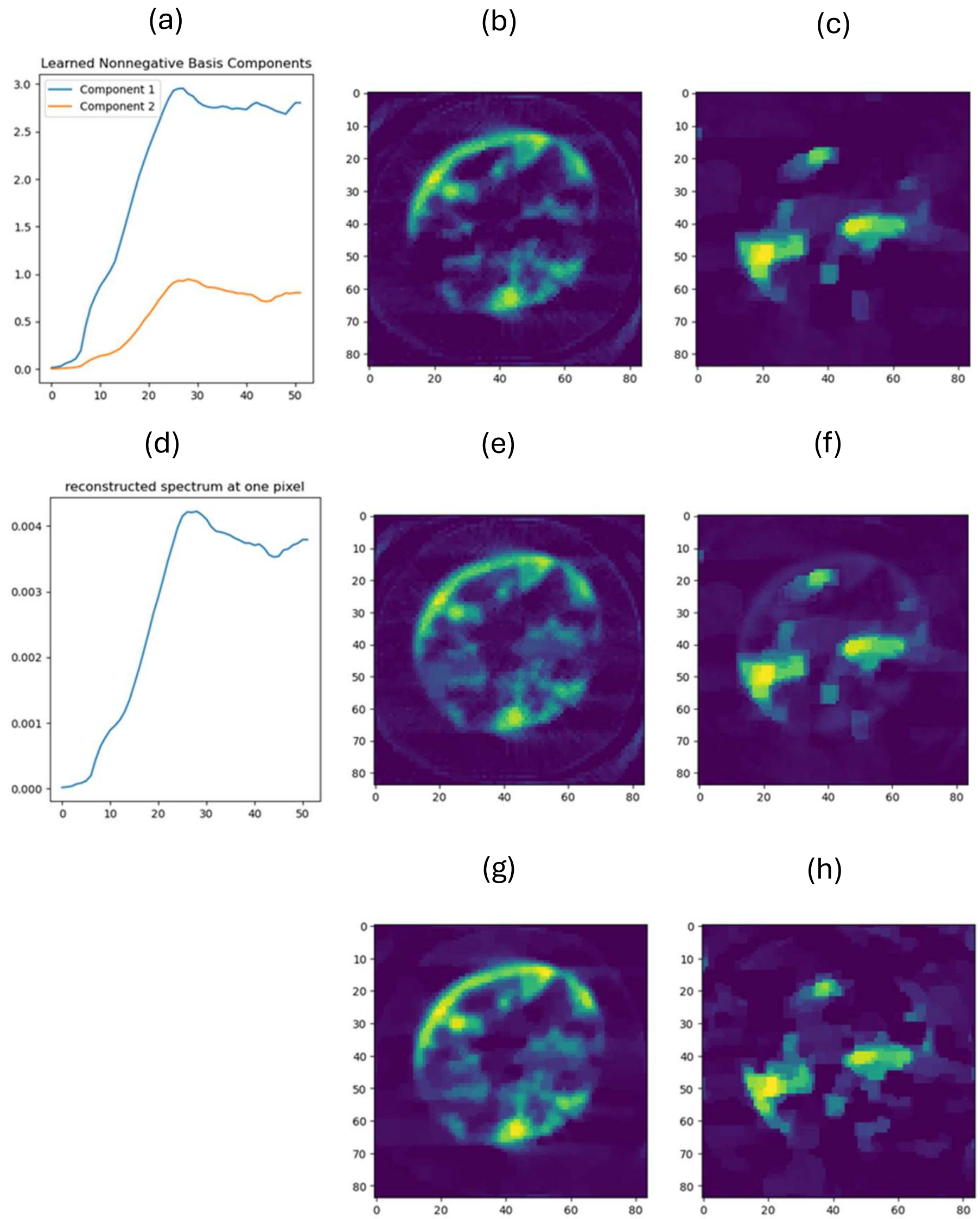

Fig. S15. Synthesis mode. (a) two learned basis components from 2D nano-XANES data using non-negative decomposition. (b) and (c) are the spectro-tomography reconstruction of a slice based on the two learned basis components. (d) is the synthesized spectrum at one pixel of the slice. For comparison, we fit the synthesized spectrum at every pixel with two reference spectra for Fe^0 and Fe^{3+}, and obtained their distributions in (e) and (f), respectively. (g) and (h) are the direct spectro-tomography reconstruction with two reference spectra for Fe^0 and Fe^{3+}.
