# Supplementary material for: Sparse X-ray spectro-tomography for high-sensitivity three-dimensional chemical imaging at the nanoscale
Source: Nat Commun. 2026 Sep 16;17:10867. doi: 10.1038/s41467-026-77821-5 (PMC13672795; doi:10.1038/s41467-026-77821-5)
Supplement: Supplementary file 2 [file 41467_2026_77821_MOESM2_ESM.pdf]

Description of Additional Supplementary Files

File Name: Supplementary Movie 1
Description: 3D rendering showing volume destructions of Fe, Cr, and two Fe oxidation states of the test sample.

File Name: Supplementary Movie 2
Description: 3D rendering showing the volume distribution of the Co, Mn and two Co oxidation states of a cycled NMC811 battery particle.
