# Supplementary material for: Sparse X-ray spectro-tomography for high-sensitivity three-dimensional chemical imaging at the nanoscale
Source: Nat Commun. 2026 Sep 16;17:10867. doi: 10.1038/s41467-026-77821-5 (PMC13672795; doi:10.1038/s41467-026-77821-5)
Supplement: Supplementary file 3 [file 41467_2026_77821_MOESM5_ESM.pdf]

nature portfolio

Peer Review File

Sparse X-ray Spectro-Tomography for High-Sensitivity Three-Dimensional Chemical Imaging at the Nanoscale

Corresponding Author: Dr Hanfei Yan

Parts of this Peer Review File have been redacted as indicated to remove third-party material.

This file contains all reviewer reports in order by version, followed by all author rebuttals in order by version.

Attachments originally included by the reviewers as part of their assessment can be found at the end of this file.

Version 0:

Reviewer comments:

Reviewer #1

(Remarks to the Author)
Review of "Sparse X-ray Spectro-Tomography for High-Sensitivity Three-Dimensional Chemical Imaging at the Nanoscale"
June 4, 2025

Nanoscale X-ray spectrotomography measurements using a scanning probe remain challenging and time consuming, due the relatively slow nature of scanning relative to full field imaging, and the need for many projections at different angles as well as energies. Put simply, the authors devised a data acquisition strategy and tomographic reconstruction algorithm that reduces the acquisition time necessary to collect hard X-ray spectrotomograms. They did this while maintaining the overall data quality in terms of high spatial and energy resolution. The authors demonstrated their data acquisition strategy and tomographic reconstruction algorithm with a computational simulation, a known test sample, and a battery material sample.

The work seems cutting edge and they are tackling a problem worthy of a high impact journal. Speeding up acquisition times will allow higher throughput. This will allow more researchers to study more systems, enabling more discoveries. The figures are clear and, in my view, they contain the right amount data to prove the point that the method works while being concise. The references include appropriate and recent works that I am familiar with. The paper is well written and just a few typos that would be caught in the next revision.

The spectrotomography results are impressive. The effect of stochastic energy sampling is fascinating (Fig 2a, c and d), and may stimulate new ideas in other fields of imaging. I am not an expert on the details of tomography reconstruction algorithms, so I am unable to comment on validity and novelty of that aspect of the manuscript, if that methodology is sound or how reproducible that is. Overall in my view the method seems to work with nice examples.

Being a bit more critical, X-ray spectro-tomography is not a new technique. Its an existing technique with several published examples (the authors include several examples in the references). Since it exists, I disagree with the claim in the abstract that their innovation is "making such [spectrotomography] measurement practically feasible" (line 15). I also disagree with similar statements elsewhere in the manuscript, that "the scanning mode would take impractically long time" (line 41), or "This prohibitively long acquisition time makes spectro-tomography impractical in the scanning mode" (Line 81). The authors should reword claims that the method presented here enabled a jump from something prohibited to something now possible, especially when the examples presented don't capture dynamic processes. The manuscript still reports quite a long acquisition time… more on that point later.

What the manuscript is reporting is a neat original method that makes an existing spectro-imaging technique faster. But there are other reported methods that make this technique faster, like references 14-16. There appear to be no claims of higher spatial or higher energy resolution, or a measurement that is more accurate relative to collecting all angular projections to meet the Crowther criterion, at all energies across an absorption edge. The work presented seeks to achieve an end result of equivalent quality, but faster, by requiring less data and therefore less acquisition time.

That to me is where the impact of the work lies: The reduction in acquisition time. The authors state that their method "reduces the data requirement by two orders of magnitude" (line 14). They also state that they are "reducing the amount of data and measurement time by two orders of magnitude" (line 47). Then, way way at the back of the manuscript (in fact it is the 3rd last sentence of the manuscript) we finally learn that the data acquisition took "about 10 hours" (line 346). In the way it is currently written, its not clear if about 10 hours was for the test object, or the battery material sample, or both combined (I assume it was for one of the two sample measurements). Why is this important detail buried at the very end of the manuscript?

Now what about a direct comparison of the new results, with other methods mentioned in the second paragraph of the introduction? I found no hard numbers to determine if this method was faster than other methods for the examples presented. The authors should clearly say how long would the measurements have taken by other methods. All I can do is assume it was 2 orders of magnitude larger, so 1000 hours without stoichastic energy sampling? What about the other methods that also speed up the acquisition time? How does this new method compare to other methods needs to be addressed, and the authors must make that comparison easy for the reader. There should be a paragraph clarifying details of the acquisition times, the benefit here, and the benefit by other sparse methods. Without that comparison, I found it difficult to assess if the work belongs in a high impact journal. 10 hours is still quite long in my view. Reducing it down to 1 or 2 hours, that would be something amazing with a big impact. I also suggest stating the acquisition times within the captions of Figs 3 and 4. Also, Fig 3a and Fig 4a could use spatial scale bars.

There is this statement, line 55 - "From one measurement with an acquisition time no greater than that of conventional tomography, we obtained chemical states of Co and elemental distribution of Co and Mn in 3D." I find this statement too vague. What is meant by conventional tomography? What comes to my mind is the single energy full field projection version, where a full tomogram can be collected at a synchrotron beamline in well under one minute. Furthermore, I am skeptical that a single energy tomogram in scanning mode at this resolution level and this field of view would really take 10 hours?

Lastly, I am very surprised the authors didn't mention even one sentence about the other benefit of reducing the acquisition times: Reducing radiation damage artefact by reducing the X-ray dose.

(Remarks on code availability)

Reviewer #2

(Remarks to the Author)
The authors present a study performing spectral CT which samples energy and angle simultaneously, which can be used to develop a spectral CT image with similar sampling time to conventional CT, lowering sampling requirements than current approaches.
The approach relies on,
• Stochastic energy-angle sampling to sample the sinogram
• Iterative tomography and TV regularisation to produce a spectral CT result
• Fitting the energy variation to a set of prior reference spectra as part of the reconstruction

The text is accessible and authors have demonstrated a significant speed up in spectral tomography. The result is novel and will be of interest to the wider imaging and materials science community but there are some concerns on the presentation of approach, particularly it's specific limitations.

Concerns –
Use of prior information
The use of prior information should be explicit. The abstract makes no mention of prior information being intrinsic to the approach. The conclusions suggest general applicability such as for in-situ experiments, but given all states need to be known it's not clear how this would be achieved.
The author references other spectral sparse approaches, and while they are not as efficient, they are general, and this distinction isn't clear.

Overfitting and Underfitting Risks
The approach specifically uses prior information, or reference spectra to guide the reconstruction towards the result, with the result having to fit to a mixture of k reference spectra or k species in the sample.
The practical challenge is in knowing the k species present exactly.
If I underestimate – e.g. if there are 3 species and I use 2 references.
If I overestimate – e.g. there might be 5 species and in fact there are only 2.
If a dictionary of references was used?
The weights will still fit and produce an outcome and depending on backgrounds variations and other factors you will get overfitting and spurious results.
The impact of noise study in supplementary information points to a higher signal to noise requirements, the samples studied are all concentrated, and Figure 3 briefly comments on discrepancies in fitting due to noise.
The authors should present a systematic study of over and underfitting or be explicit on the limitation that this has only been shown to work for the exact case where the species in the sample are exactly known and above a signal to noise threshold. i.e. concentrated samples.

Figure 3- The Fe^{3+} image in particularly has quite different spatial features in the bottom half of the image likely due to overfitting which could be discussed in this context as incorrect conclusions could be drawn depending on the questions being asked of the data.

2D XANES as a Precursor
If k isn't known the authors state that a 2D XANES could be used to work this out, but this approach isn't tested in the work. If the 2D XANES only results in using PCA components and doesn't spectrally unmix the results, the tomography fit to a mixture of PCA components, rather than reference spectra is unlikely to produce clear results, particularly with noise.
The use of 2D XANES should either be validated for the examples where it is acquired or as it's a speculative comment, should be removed.

The conclusions mention the expansion to in-situ studies. If the states present haven't been measured previously the outcome would be questionable so it would require a mixture of 2D XANES and known references. This also highlights the need to examine the use of PCA components or to revise the text to be clearer on the limitations.

Specific Comments
(Page2 Line 49) claims to directly reconstruct the chemical states where it's a weighted fit from reference data, it's not a direct reconstruction.

X-ray measurement
Please state how the reference spectra were obtained and processed. Alignment issues are very common in nano-tomography but no mention of pre-processing is made or any impact on this method – please comment

Figure 3
"These results reveal a strong spatial correlation between Cr and Fe0." - should be "demonstrate" or similar, as it's a stainless-steel sample, it's an expected result.
(c) The authors state excellent agreement but the spatial resolution is clearly different. The authors comment in the text on the Crowther criteria, but the use of TV regularization will smooth out results. The resolution is never estimated using FRC or a similar technique and some comment on the resolution or smoothing would be beneficial to understanding the approach.

(Remarks on code availability)
Code is available on github,
It covers the examples presented and examples can be reproduced.
The code has no docstrings or comments which would significantly help re-use or understanding.
The notebooks also have no comments.

Reviewer #3

(Remarks to the Author)
The following review is for the manuscript titled 'Sparse X-ray Spectro-Tomography for High-Sensitivity Three-Dimensional Chemical Imaging at the Nanoscale' by Hanfei Yan and collaborators.

In this manuscript the authors tackle the need to record large datasets for spectro-tomography. In the most general approach specto-tomography requires a fully sampled tomogram for each energy point of a spectrum of interest to reliably recover the samples energy specific response in each resolved volume element. The need to record projections in 2D, a sufficient number of projections NC within a tomogram (Crowther limit) and a full tomogram for each energy position is what imposes long scan times and a wide application of the method.

In previous works by other authors (cited within the manuscript) the need for a fully Crowther sampled tomogram at each energy point was alleviated by the assumption that the sample under investigation most likely only was made up of a small number NS (<< NE of energy points) of distinct compounds with distinct spectral responses, without assuming what these compounds and their spectral responses are. The gain of this approach comes from the argument that each voxels spectral response in the final volume, whatever its mixture of compounds might be, can be expressed though of a base of NS (or more) base vectors. The authors of that study proceeded to take the equivalent of (~ NS+1) fully Crowther sampled tomograms in terms of number of recorded projections. As the spectral response of the compounds / the base vectors was not fixed / unknown, the ~ (NS+1)* NC projections were spread over all NE (>> NC) energy points, effectively lowering the angular sampling at each energy point below the Crowther limit.

The authors of the present manuscript extend on this previous result. They added one further assumption: That the number of components and their spectral responses is exactly known.

Where the previous approach had to iterate between reconstructing NE angularly under-sampled tomograms, running a principle component analysis of the voxels spectral responses, limiting the number of components, enforcing the limited set of base vectors and refining the tomographic reconstruction, the presented method can take a more direct approach: directly

minimizing the set of linear equations describing both the tomographic projection (Radon transform) and the energy specific response of the sample.

The authors report the main results to be: their joint spectral-tomographic reconstruction algorithm, their supposedly better performing stochastic energy sampling approach and the demonstration of both of real data.

Within the manuscript the authors provided links to both the data and the code used to obtain the presented results. As a reviewer I very highly appreciated this, as it allowed me to see what really happens within the code and to base my review responses not just on assumptions, but rather try it myself with the authors code and data.

I believe that the presented work is unique, sound in the development of the joint reconstruction algorithm and worthy of being published. I do however argue that the results are over-sold and that the manuscript requires major revisions in regards to the potential gains of the presented approach to spectral tomography before it can be published.

In the following I will list my concerns, the authors need to address in their revision of the manuscript.
I will start with the major ones:
(M1) For me the biggest grievance with the manuscript in its current state is the notion that information can be created out of nowhere. To reconstruct volumes of M independent coefficients for M linear independent spectral response vectors (material components) for each vowel with a volume, at least M * NC projections will have to be recorded. The claim that any smart measurement, or algorithm that does not introduce further knowledge and assumptions can recover M independent coefficients per voxel from only NC projection without loosing resolution or inventing something within each pixels composition is just wrong. The number of measurements (knowns) needs to be equal or larger to the number of reconstructed unknowns.

The authors claim “achieving perfect reconstruction” from an under-determined problem.
This just can not be.

(M2) When comparing their stochastic (“random-uniform”) energy sampling approach to the previously published “interlaced” approach, they, in my eyes, make multiple mistakes that happen to give their stochastic approach the edge over the others methods.

The “interlaced” approach was neither meant for not claiming to reconstruct M components from only NC projections. The authors of that study rightfully recorded more than M * NC unique projection angles in total. Here the authors hand the previously published energy sampling approach a problem it was not designed for.

(M2.1) The problem is not just under-determined, but the problem size and chosen spectral responses are chosen such that (i) the “interlaced” energy sampling is disadvantaged, and (ii) the assumption needed for and thus the strength of the presented joint spectral-tomographic reconstruction is totally disregarded.
(M2.2) With that I mean that the presented algorithm hinges on knowing the spectral responses for all components within the sample, which are all known prior to the reconstruction of the data. The authors explain that these spectral responses could be obtained from a fully energy sampled set of projections, taken prior to the tomogram, using PCA (This only holds true if in the projection in question the components appear in different projected pixels with linear independent coefficients. Thus it could fail simply by an unlucky sample structure and choice of projection angle).
(M2.3) The authors did not at all take into account that, knowing the spectral response of all material components within the sample, they could have selected a set of energy point where the multiple spectra show the largest differences to each other. Instead of measuring only energies that allow to separate the various components, the spectra contain many energy points were the spectral response from the materials is very similar or shows a smaller difference than at other energy points.
(M2.4) One could have picked a small set of energies with the most orthogonal spectral response vectors. Instead many adjacent energy points have very similar spectral responses as their neighboring energy points. This becomes very clear when plotting the difference of the two spectra used in the simulation:

All energy points where the difference of the spectra is close to zero will show little to no contrast between the two materials. There is no reason to measure those in the first place.

Looking at the remaining energy points it becomes clear that there are stretches of adjacent energy points where the contrast between the, in this case, two materials is similar.
Measuring those groups adjacent energy points with similar responses at adjacent projection angles means there is no way to differentiate well between the two material components for those angles. That is why the interlaced energy sampling strategy is at a disadvantage here to the “uniform-random” strategy, where there is a higher chance to jump between points energy that are to each other linear independent in their spectral response, meaning it is more likely that the two material components can be separated for those adjacent projection angles (this arc of angles).
(M2.5) I argue (and I tested) that just randomizing the order of the used 52 energy points, but keeping that same order within each interlaced arc does have the same positive effect. Additionally it does still come with a homogeneous angular sampling for each energy, avoiding that by chance nearby projection angles are measured by the same energy, resulting in an

unavoidable under-sampling of those energies from different projection angles.

The best results I achieved by combining a sampling strategy that gives constant angular sampling within one energy and only measuring energy points from the known spectra that allow the best possible separation of the two material components.

Below my results using the authors simulation and reconstruction code:
From left to right I varied the number of energy points of the available spectra I ended up using. On the far left, the most extreme case of just selecting two energy points to differentiate the two material compoents.

In the bottom row, I plotted the mean absolute difference in pixel value between ground truth and the reconstructed objects.
The color of the dots indicated the energy sampling method used:
blue – the authors stochastic method
orange – interlaced
green – interlaced, but at a once randomized order

(M2.6) In all cases I used the (to few) 181 projection angles (as the authors) and only used values within the circular disk (not the corners of the reconstructed array) to evaluate the quality of the construction.
No matter how many energies were used, mixing the initial order of the energy points and going through them again and again (green) does result in equal or lower errors than just rolling the dice for each angle (blue). Measuring fewer and fewer unnecessary energy points does reduce the error achieved by simple "interlaced" energy sampling (orange).

The results become even better (for all three strategies) if a sufficient number of projection angles (> 2*Nc in this case) is recorded.

While I do not believe that the authors chose the simulation parameters (128x128 pixel objects, 181 angles, 52 energy points) on purpose to have their method come out on top, this choice is the reason their algorithm worked better in this case. I want to stress that it was a sub-optimal choice to "run the experiment".

I want to follow by some less sever, but still important concerns that require addressing:

(m.1) All figures in the manuscript are from rather low number of pixel data. That is the nature of the simulated and recorded data. And that is fine. But having not having the default sub-pixel interpolation of matplotlib.pyploy switched off does introduce a smoothness in the data that is just not there and might trick some readers. The authors should use the optional argument interpolation='None' in all their figures to show square pixels of homogeneous value.

(m.2) In and around Figure 2 the authors present a simulation of a two-compound test sample. In line 139 they claim achieving "perfect" reconstruction. While the word "perfect" in itself can not supported (see above), this could not possibly be seen from the figure as it is.
I suggest that the authors plot the difference between the ground truth and their reconstructions in a diverging colormap.
Additionally they should find some error metric to report the non-zero difference between GT and reconstructions with some quantitative number.
Running the authors unaltered code and plotting the difference between GT and reconstructions as suggested, it becomes obvious that there is a non-zero difference (examples shown within the next comment).

(m.3) When presenting a new method based around a random number generator it is good practice to study if the seed of for the random number generator can have any impact on the quality or even consistency of the obtained result when everything else remains unchanged.
Having access to the code of the shown simulations I just ran the simulation multiple times with different seeds for the random number generator and found the quality of the reconstruction to vary. See below a selection of very few of the different results I obtained for different seeds.

Having tried many seeds, I can say that overall many of them seem to obtain results of similar quality for the simulation at hand. But there is also a decent chance that a randomly picked seed fixes a energy to angle selection that results in multiple times larger errors.
As the selection of energies to each angle happens before the data taking, there is no way of reducing the impact of a potential bad selection purely by means of reconstructing the data differently once it is taken.
This behavior needs to be honestly discussed in detail.

(m.4) The addition of TV into their minimizer to be able to deal with noisy data was well thought out and important. But TV is “just” enforcing a certain smoothness within the obtained reconstruction / penalizing strong gradients. I would like to have seen a discussion of other options, including some that take into account the nature of the noise in the data (Poisson noise).

(m.5) The push towards joint reconstruction algorithms within the community is something I see as a very positive thing. Explaining problems as a whole and solving them as a whole as well, allows to use every bit of information wherever it might be. The authors should discuss the computational need / requirements and most importantly the scaling of their algorithm with the problem size. While playing with the authors code myself, I noticed that the minimizer does require a considerable amount of time to iterate through problems of very small size. How does the reconstruction time scale when the problem size doubles, quadruples, ect?
I want to thank the authors for the work they put into this manuscript.
I very much appreciated the way their data and their code was made available for this review.
Even if this is part of the reason my review has ended up being what it is.

I am looking forward to reading the reworked version of the manuscript to this work.

(Remarks on code availability)

Version 1:

Reviewer comments:

Reviewer #1

(Remarks to the Author)
I feel the authors addressed my questions and concerns in the rebuttal and made appropriate changes to the manuscript. I recommend publication.

(Remarks on code availability)

Reviewer #2

(Remarks to the Author)
My original comments focused on the need for the manuscript to be explicit and unambiguous about the role of prior information in the proposed approach, and the resulting limitations—in particular the risks of overfitting and underfitting. Specifically, the method relies on a priori knowledge of the chemical states present, and its validity is therefore restricted to well-defined chemical systems. Clarifying this scope does not detract from the novelty of the work, but is essential to ensure appropriate interpretation and application.
While the authors’ responses to the reviews are generally reasonable and well argued, the corresponding modifications to the manuscript are comparatively brief and do not fully reflect the depth or clarity of those responses. As a result, key limitations and assumptions still feel understated and underexplained in the revised text.

My intent is not to unnecessarily extend the manuscript, but rather to ensure that the core assumptions and limitations are clearly visible to the reader, not only explained in the rebuttal.
Abstract (Page 1)
Revised text currently states:
“This is accomplished through sparse and stochastic energy sampling combined with a joint spectral-tomographic

reconstruction algorithm that performs the spectrum fitting and 3D reconstruction in a single step."
Reviewer request:
The abstract should be more descriptive and explicit about the reliance on prior chemical knowledge. For example: "…performs the fitting to known chemical signatures present in the sample and 3D reconstruction in a single step."
This small change would immediately clarify the scope of applicability at the highest level of the paper, otherwise explicit statements on exact states are only in the supplementary information.
Overfitting and Underfitting
I thank the authors for adding the new figures in Supplementary Figure S3 however, the caption and accompanying text remain misleading.
Supplementary Figure S3 Caption
Current caption states:
"(b) Spectro-tomography reconstruction with three states, while only two states exist. It leads to a bigger error as compared with the correct two-state reconstruction."
Reviewer comment:
The issue is not simply a larger error. The more important consequence is the potential misidentification of a non-existent chemical state and distortion of the spatial distribution of the true states. This is a qualitatively different and more serious limitation than a generic increase in reconstruction error.
I recommend revising the caption and/or supplementary text to explicitly reflect this, for example by noting that overfitting can introduce spurious chemical states that do not exist in the sample.
Supplementary description - Impact of hyperparameters and number of states
The revised manuscript currently states:
"Proper tuning and the correct selection of reference spectra is essential for achieving optimal results, especially when the data is noisy."
Reviewer request:
Please make this more explicit. Suggested revision:
"Proper tuning and the exact selection of reference spectra that are known to be present in the sample under investigation is essential for achieving optimal results, especially when the data is noisy."
This clarification is important, as the risk is not merely suboptimal performance, but incorrect chemical interpretation.
On the topic of overfitting the authors responded -
Just like any spectrum fitting in XAS, human-in-the-loop with domain knowledge is needed to determine what are the possible states that should be used for fitting. This can be either determined beforehand knowing the reference spectra or by an additional nano-XANES measurement. The domain scientist needs to make the judgement (with combination of other knowledge) whether there is an overfitting or underfitting issue, just like any fitting method. At this development stage, we do not recommend determining number of states from the very sparse spectro-tomography dataset itself (see below discussions). We emphasized this point (see above response). Also, a study of overfitting is added (see below response).
And later comment

"In general, the algorithm still works, but there are more errors in each state, leading to a higher mse. Even for perfect data, state 3, which doesn't exist, is not completely zero. In theory, it is possible to determine number of states by minimizing the mse error (from 2e-4 with incorrect three states to 4e-5 with correct two states) from spectro-tomography reconstruction process. However, due to imperfections in real data (misalignment, noise, …), this is not so reliable at the current development stage."
But only the 1 line statement above was made and the potential pit falls still feel unclear in the text. Adding some of the rebuttal text would improve overall clarity.

(Remarks on code availability)
Code presentation is poor and would benefit from following a standard such as JOSS on presentation.

There are no comments in the code or notebooks and no doc strings.

There is no README, requirements.txt or environment.yml

This is very much a demonstrator code, no a re-usable resource.

Reviewer #3

(Remarks to the Author)
I thank the authors for adjusting their manuscript with regard to the other reviewers and my comments.
However, it feels that in regards to most concerns raised by the reviewers the authors looked for the smallest possible change that might appease us or simply not adapt anything.

I want to be very clear that I believe the presented work is worth publishing. But major changes to the manuscript will have to happen before that. From my side, further changes do not require any additional experiments, data taking or simulations. The already obtained results just need to be discussed more honestly.
My suggestion to the editor remains "major revisions".

I will further elaborate on my thoughts in detail, focusing on my previous comments and how they were addressed by the authors.

(M1) My concern remains: The authors do not discuss that their method creates information out of nothing. Yes, they solve a minimizing problem. But the whole manuscripts reads as if the same number of spatial resolution elements and spectral components per spatial resolution element can be recovered from way fewer measurements. In fact, sometimes not even enough measurements to properly reconstruct a single spectral component in the tomographic plane.
There is only one place place in the manuscript where this shimmers through: In the added text around line 197, showing that solving for multiple from spectral components within a tomogram reduces the resolution in the obtained tomographic reconstruction → lowering the number of spatial resolution elements for the sake of recovering multiple spectral components. Effectively this shows that the number of unknowns to be solved for from a fixed number of knowns can not magically increase; just shifted around across dimensions.

(M2) The authors did not address that they misrepresent someone else work that they cite, by doing something that is similar but inherently wrong and specifically avoided by the original publication. The authors should highlight that they use and adapted version of the “interlaced” energy sampling approach (see citation) but with too few knowns / measurements contrary to the original work.
Again this feels like the authors try to dance around the apparent problem that the presented approach tries to work with a way under-determent problem.

(M2.1) If the number of spectral points and sub-Crowther-limit angles is a commonly used approach at the beamline, then this should be pointed out to the reader. It should be discussed in detail that these numbers are not sufficient for the tomographic problem and not optimal for the decoupling of spectral components (of that many spatial resolution elements).

Any comments on speed by the authors are misplaced here.

(M2.2) An unlucky choice of sample and projection angle(s) for the 2D XANES scans could, due projection, end up with the respective spectral components to contribute with the same relative fractions in each (projected) pixel. While it is unlikely, it is not impossible. In fact there are an infinite, be it artificial, samples that, when projected along the right angle, show the same relative mixing of spectral components in all pixels. One example would be a chess board like pattern projected along either of the sides of the board. A XANES scan of such an, in the real world unlikely, projection could not decouple the multiple spectral components, as the projected response can be explained with one single spectral base vector.

(M2.3 and M2.4) The word “orthogonal”, or derivatives (like linear “independent”), does not appear anywhere in the article or the supplementary. Again it seems that the authors try to avoid the shortcomings of the presented approach.
If a method relies on some sort of prior knowledge (the spectral response base vectors of all components) it is well worth discussing that some photon energies do not give any (additional) information and thus should not be part of the energies used to measure. Choosing a subset of energies that the resulting spectra are as “linear independent as possible” is not something that can be brushed off for a future paper. It is known already know and might as well be reported as a best practice within this manuscript.

(M2.6) I can grasp that it is not the “goal” of this work to take sufficient data. But then it must be clearly stated and discussed. This concern of mine is a repeat of the previous points. The whole manuscript seems to explicitly try to hide that not enough information is recorded to have both the full number of spatial resolution elements and spectral components per spatial resolution element.
The authors have to make this very clear throughout their manuscript. As it stands right now, it is not mentioned or carefully hidden in a very few selected words hidden through the manuscript. To a reader not deep into the problem, pretty much the whole manuscript seem to report that the same kind of result can be achieved 100 times faster. Which is not the case.

What the authors phrase as “Of course, adding more data always helps to improve the result, and the difference between methods would diminish, but that’s not the goal of this work.”, I would phrase as: adding more data always to improve the result, and the difference between any of the methods and the GT diminishes as finally sufficient data is recorded.

In summary: unless the authors are verbose in the discussions of the key differences and shortcomings of the presented method throughout the whole manuscript, I can not advise publication of the manuscript.
As long as the reader needs to be proficient enough in the field and the math to read between the lines to know that …

- compared to previous methods, this one requires prior knowledge of the spectra components to solve for
- that real pure randomness in the angle-to-energy selection is worse than even interlaced with a randomized, but repeating sequence.
- that making a smart sub-selection of energies is possible (because the spectra are known), not hard (orthogonality of the resulting sub-spectra), and always advisable.
- the problem is way under-determined (effectively just one sinogram and in many examples not even enough angles according to Crowther limits for a single spectral component
- that taking more / closer to / exactly N_spectral times N_crowther measurements improves the quality in all cases and (thus) also makes differences between the methods disappear

• That doing it this way is maybe the only option in case of limited time. But that it is sub-optimal and the obtained results CAN NOT be equivalent in quality to “doing it right”.

… I can not advise publication of the manuscript.

Once these things are very openly and very very clearly discussed in the article, I could advise a publishing of the results.

(Remarks on code availability)

Version 2:

Reviewer comments:

Reviewer #2

(Remarks to the Author)
Thank you for the requested changes. I'm not sure an MSE search will in fact work, but this is only outlined as an in principle approach and the article is sufficiently clear that prior information is needed.
No further comments.

(Remarks on code availability)
Code has been significantly improved.
The authors have now added a readme, installation instructions and usage guide as well as adding descriptions and comments in the code and notebooks to aid understanding and reuse.

Reviewer #3

(Remarks to the Author)
The following review is for the revision of manuscript titled ‘Sparse X-ray Spectro-Tomography for High-Sensitivity Three-Dimensional Chemical Imaging at the Nanoscale’ by Hanfei Yan and collaborators.
I believe that the presented work is still unique, that the presented joint-spectral-tomograhic reconstruction via a ADMM minimizer and TV is a very important tool for any spectral tomographic measurements in the future, that the presented experimental results are well made and presented and that the manuscript is worth publishing.
However, my main criticisms since the very first review still stand and have not been addressed sufficiently in the current version of the manuscript. Hence, I still believe that the manuscript should receive major revisions before it can be published.
I will briefly summarize my major concerns again before a point by point reply to the authors answers to my remarks made in the previous review round.
(1) The authors still do not clearly state that solving for two (“full resolution”) tomograms of two respective chemical states within the sample will require at least two times the Crowther limit worth of projections to be recorded. Likewise solving for 10 different chemical states would require at least 10 times N_Crowther projections to be recorded. The only point in the manuscript in which this slightly shines through is in line 232:
“Because spectro-tomography involves solving a larger number of unknowns, the state map of Fe0 exhibits a lower spatial resolution of 80 nm (half-period), compared with the 50 nm resolution achieved in the Fe reconstruction (also see Fig. S12).”
Otherwise this is never explained or discussed. It does not show up in the introduction of the method. It does not show up in the discussion. Rather the whole manuscript gives the impression as if just a “single tomogram”, meaning one set of N_Crowther projections at randomly chosen energies, is sufficient to solve for the two, or in extension any number of chemical components, within the sample without sacrificing spatial resolution.
I agree with the authors that week long measurements of (in their example) 180 projection angles at each of 50 energy points are close to not feasible. But instead of claiming that 180 projections are sufficient to solve for 2 or any other number N of chemical components within the sample without loosing spatial resolution, one would only have to say that solving for N chemical components (at “full resolution”) does not require 50*180 projection angles in that example, but N*180 projection angles. Measuring only 180 projection angles is not enough for the case N>=2. While the presented joint reconstruction minimizer will find a minimum and the TV will make sure it is somewhat smooth, this smoothness is not the same as actual spatial information.
As mentioned in the first review, this became already clear in the way the authors implemented the work by Z. Gao et al. with the evenly sampling in angular space. Z. Gao et al. did account for having to solve for (at least) N chemical components by measuring at least N*N_Crowther angles. The authors of the current manuscript did ignore this need for extra information both in their own implementation, as well in their adaptation of Z. Gao et al.’s method.
Yes, having to record at least as many “full” tomograms as there are chemical components does take longer than just taking N_Crowther projections for N>1. But it is generally still much faster than taking

(in the authors example) 50 full tomograms, just because the reference spectra for the 2 spectral components had 50 energy points. The gain in speed might not (always) be "two orders of magnitude", but it is still a gain. And it just is so obvious that it does need to depend on the number of chemical components that need to be differentiated.
Not measuring enough angles per chemical component is possible. It does save more time. The presented algorithm can still find a smooth looking minium solution. But it will ultimately have to trade in some spatial resolution (equivalent for recording N_Crowther projections at a single energy) for the sake of solving for multiple spectral components.
This is a hill I am willing to die on. If this does not make it clearly into the introduction of the method AND the discussion, I can not advise for the publication of the manuscript. Information is not just created out of nowhere.
(2) My second major issue with the presented manuscript is that the authors claim that randomness is they key for their reconstructions to work well. Only randomly sampled energies would allow the minimizer to arrive at the lowest errors and thus the hightest resolutions.
This is not true. For a method that is based on prior knowledge of the spectra of all chemical components within the sample, just picking energies at random CAN NOT BE the optimal solution.
While the authors briefly mention that there could be improvements (line 313):
"Optimizing the selection of sampled energy points could also help reduce uncertainties, and the same holds for refining the randomization process."
I strongly disagree with the word "could". I believe this wrong conclusion to be a results of the wrong implementation of Z. Gao et al.'s method and my argumentation in point (1) above.
A thought experiment would be the following. Imagine a sample with 2 chemical components. The spectra are conveniently such that they consists of only two energy points. At the first energy the first chemical component has a 1 and the second spectral component has a 0. At the second energy it is the other way around.
Measuring 2* N_Crowther projections with alternating/ interlaced energies results in a dataset that can easily be separated in a tomogram for the first energy which only has contributions from chemical component 1 and an interlaced tomogram for the second energy which only has contributions from the second chemical compound. Both tomograms are according to Crowther limit. Both tomograms have equal angular sampling. Neither tomogram would profit from further angles (except in the presence of noise). Both tomograms can be "perfectly" reconstructed, which would perfectly separate the two chemical components with "full" resolution.
Measuring the same 2* N_Crowther angles, but flip a coin at each angle with of the two energies COULD results in the very energy ↔ angle combinations, which would give the same results. But in most cases of this random coin tossing experiment, the energy to angle assignments would end up with at least a few occurrences of the same energy being assigned a few consecutive angles in a row, even if overall the same number of N_Crowther angles is measured for each of the two energies.
The resulting tomograms could again be split into tomograms for energy 1 and 2 which only hold information of chemical component 1 and 2 respectively. Both tomograms would have N_Crowther angles. But expect for two of 2**(N_Crowther) cases, the two tomograms would not be samples equally in angular space. That means each tomogram has angular regions of (too) dense and (too) sparse sampling. While too dense sampling is not harmful for the reconstruction, it is a bit wasteful. Too sparse angular sampling leaves (some) "cake pieces" of not measured regions in Fourier space. This missing information can not be compensated for. Not even by a ADMM minimizer. Not by machine learning. It is simply not measured. This means that in the reconstructions of both tomograms there will be directions corresponding to the respective missing information that would be "not perfectly" reconstructed. Resolution would be lost.
Just assigning energies from a pool of energies at random to projection angles can NOT be the optimal solution.
While the thought experiment is artificial and would not appear like that "in the real world", it does show that "fully random" can not be optimal.
The thought experiment can be extended to include many more energy points in the spectrum. If all the additional energy points are identical to the first two (0 in one and 1 in the other component) and they alternate in assignment, then the two described methods would approximate each other closer and closer.
If however, like most common in reality, the spectra would be arranged / sorted by which of the chemical component they contribute to (1) and which one they do not contribute to (0), and still just 2* Crowther projections would be recorded, then the first method would create tomograms where large consecutive angular sections are only measured for one component, then for the other and so on, while the random method, overall would roughly perform as before.
In this world, where one chooses to ignore the knowledge that many of adjacent data points in the spectra do not deliver independent information, the fully random approach outperforms the idea of equal angular spacing.
But educing the spectra to a low number of meaningfully diverse energy points would result in the perfectly sampled tomogram from the initial argument.
I argue that by needlessly taking too many data points in the spectra, where many adjacent energies offer little to no new information compared to their neighbors, the "fully random" approach is advantaged. This is further obscured, by not even recording N* Crowther angles for each of the chemical components within the sample.

I want to say that is is perfectly fine to record not enough angles and to fully randomly assign the energies of the known spectra to whatever numbers of angles is recorded.
The former results in an overall loss of spatial resolution, while the later is easy to implement, but nearly always a sub-optimal way of doing it, which will result in random directions in the respective tomographic slices per chemical component to be worse than they would have to be.
Taking many energy points far from the absorption edges, where the spectral contributions of different components might be close to identical is an obviously wrong way to go. No one would argue it is smart to add these to the measurements just because they are in the known reference spectra, if they do not allow the two chemical components to be separated. It does not matter if they are later assigned randomly or not. They should not be measured for the goal of telling the separate chemical components apart.
While finding the optimal of way choosing which energies to measure and which ones not to measure and how to assign them to the to be measured projection angles might be beyond the scope of the paper, claiming that randomly assigning energies to angles is vital for the method to work is just wrong.
Just having half a sentence in the discussion that says that choosing smarter ways of assigning energies “could also help” with getting better results just does not cut it when multiple times throughout the manuscript the randomness is argued to be the key for the joint-spectral-tomography reconstruction to work well.
I believe random is simple, easy, but neither needed nor optimal.
In the following, I will try to adress the authors responses to my previous review comments in a brief manner:
(M1) Yes, I believe the authors that they demonstrated that it is feasible to perform chemical tomography they way they did. I agree that the point of the paper does not have to be about what is the optimal way of doing it. All I ask is that they discuss point (1) from above more clearly. No matter if one uses direct inversion or a minimizer: solving for N chemical components with only 1* N_Crowther angles in the measurement WILL have to come at the cost of a reduced spatial resolution in the obtained tomographic reconstructions for the N respective chemical components. This reduction, depending on the assignment of energies to angles, might be equally bad in all components, could be stronger in some and weaker in others.
Assigning energies to angles at random will in most cases results in some directions being sampled too sparse (and others too dense). What is too sparse (and too dense) will be different for the different chemical components. Anything sampled too sparse in angle will result in a reduced spatial resolution in those directions.
(M2) Yes, the authors do explain what they did. After all this allows me to discuss the manuscript with them here. But they do not clearly convey/discuss to the novice reader that solving for N chemical components with only 1* N_Crowther projections measured MUST come with a reduction in spatial resolution.
I yet again agree with the authors that one can do it like this. I agree that it is feasible.
But I also argue that solving for 2 chemical components with 1* N_Crowther projections takes as much time as measuring 2 full interlaced tomograms at 2* N_Crowther_half for half the resolution / twice the pixel size, and that the amount of obtained information is identical.
If the beamline does not allow for scans with half the resolution / twice the step or beam size, then again it is feasible to do it as the authors presented it. But it won’t achieve the same spatial resolution at least as many fully Crowther sampled tomograms as there chemical components.
(M2.6) I do not see proof that “randomizing the spectrum is essential”. I see that it is quick. I see that it can be better than doing something that is obviously bad. But I do not see it as essential at all. I argue that there is multiple deterministic approaches that can achieve better results with even angular spacing per energy. I agree with the authors that it does not have to be the point of this work to find the best or a better method. But as long as randomness is portrayed as essential, I will argue against the publication of the manuscript.
In summary:
I very much like the work the authors presented. I love that they published the code, have simulations as well as experimental results. I agree that having a way to perform a measurement in a reasonable time is better than not being able to perform a measurement at all. I agree this is a demonstration that shows it is feasible. I agree that this work does not have to present the best way to pick which energies to measure at.
I disagree with the way that an unavoidable loss of spatial resolution when measuring only N_Crowther angles but solving for N chemical components is not sufficiently discussed and how the manuscript reads to a novice as if the very same resolution can be obtained for any number of spectral components this way.
I want to see it discussed for a novice that doing it like this is possible, but comes at a cost.
I disagree with identifying randomness in the assignment of energies to measured projection angles is essential for the method to work. Randomness is not needed at all. It is a fast convenient way of approaching the problem. While the authors hint at that there are MIGHT be better ways, I am sure there are. I do not want the authors to explore them in detail.
But I want to see it discussed that random CAN NOT be the best.
Those two major points of disagreement between me and the authors are expressed in details as (1) and (2) at the beginning of this document.

I request that the authors extensively discuss these two points in their manuscript.
Otherwise the presented data and figures are sufficient. No further simulations or experiments need to be performed. No further figures need to be made.

(Remarks on code availability)

Version 3:

Reviewer comments:

Reviewer #3

(Remarks to the Author)
I still strongly disagree with the authors comments.
We keep arguing about the same points.

As for the lowered requirements in number of projection angles:
While I appreciate that the author add simulations results to their arguments tu underline their point, a ADMM reconstruction of a 128x128px sample from 32 projections can NOT be "perfect". The information content of the 32-projection ADMM reconstruction and the FBP reconstruction of the same 32 projection is identical. It is 32 lines through the center of 2D Fourier space. Nothing more. Nothing less. Due to its design, the FBP method ends up with the to the human eye strongly visible streaks when not enough angles are recorded, while the ADMM result is smoother.
But neither is better than the other. Neither is, information wise, closer to the result. Neither has more or less spatial information than the other.
One looks better to the human eye. One might give a lower error metric due to the inherent smoothness of the ground truth.
Neither ADMM nor any TV does add additional knowledge into the reconstruction that would allow for a reduced number of projection angles to be needed. It only creates images more appealing to the human eye, but have the same information content (in Fourier space).
"More advanced regularization approaches, such as learned deep neural networks" can not add information in Fourier space that has not been measured. I agree that the former might help with the sensitivity to noise. The later, depending on the exact implementation, can just act as another minimizer, or invent information that has not been measured.

As for the key advantages of randomness: The authors do explain themselves that projections to adjacent angles ideally are recorded at energies that offer a different mixture of the target states. My argument is that this is easy to enforce deliberately when knowing the spectral responses of the target states, as this method does. Randomly picking energies from reference spectra is blatantly sub-optimal, as there are many groups of energy points with very similar mixtures of responses for the target states.
The paragraph added by the authors explains that "the probability that adjacent projections have similar state contributions becomes very small". I do not agree with the "very" in "very small", but would rather argue that "smaller".
Sorting energies by random is convenient and simple. It is better than taking energies in order from too densely sampled reference spectra. But it comes at the danger of having different random directions in every measurement separate the states worse than other directions. It will not be obvious to the users in which directions (which slices in Fourier space) the separation of the states worked well and which ones are worse.

However, overall I am glad to see the added paragraphs, as they do add the key thoughts and discussion to my main concerns.

I suggest that the authors:

- Add the point that unknown random directions (slices / segments in Fourier space) could be better / worse separate the chemical states than other directions.

Optional:
- Only keep the acknowledgement of the reviewers (and special acknowledgement of reviewer 3) if they really care. It is not needed to appease me.

I suggest to accept the manuscript for publication with this one minor revision (and one optional change).

(Remarks on code availability)

Open Access This Peer Review File is licensed under a Creative Commons Attribution 4.0 International License, which permits use, sharing, adaptation, distribution and reproduction in any medium or format, as long as you give appropriate credit to the original author(s) and the source, provide a link to the Creative Commons license, and indicate if changes were made.
In cases where reviewers are anonymous, credit should be given to 'Anonymous Referee' and the source.
The images or other third party material in this Peer Review File are included in the article's Creative Commons license, unless indicated otherwise in a credit line to the material. If material is not included in the article's Creative Commons license and your intended use is not permitted by statutory regulation or exceeds the permitted use, you will need to obtain permission directly from the copyright holder.
To view a copy of this license, visit https://creativecommons.org/licenses/by/4.0/

We greatly appreciate the reviewers' efforts and their very valuable comments. We have carefully addressed all of them in the revised manuscript. Below, we provide our point-by-point responses (in blue) and the corresponding revisions (in red).

Reviewer #1

Nanoscale X-ray spectrotomography measurements using a scanning probe remain challenging and time consuming, due the relatively slow nature of scanning relative to full field imaging, and the need for many projections at different angles as well as energies. Put simply, the authors devised a data acquisition strategy and tomographic reconstruction algorithm that reduces the acquisition time necessary to collect hard X-ray spectrotomograms. They did this while maintaining the overall data quality in terms of high spatial and energy resolution. The authors demonstrated their data acquisition strategy and tomographic reconstruction algorithm with a computational simulation, a known test sample, and a battery material sample.

The work seems cutting edge and they are tackling a problem worthy of a high impact journal. Speeding up acquisition times will allow higher throughput. This will allow more researchers to study more systems, enabling more discoveries. The figures are clear and, in my view, they contain the right amount data to prove the point that the method works while being concise. The references include appropriate and recent works that I am familiar with. The paper is well written and just a few typos that would be caught in the next revision.

The spectrotomography results are impressive. The effect of stochastic energy sampling is fascinating (Fig 2a, c and d), and may stimulate new ideas in other fields of imaging. I am not an expert on the details of tomography reconstruction algorithms, so I am unable to comment on validity and novelty of that aspect of the manuscript, if that methodology is sound or how reproducible that is. Overall in my view the method seems to work with nice examples.

We greatly thank the reviewer's positive comments and encouragement.

Being a bit more critical, X-ray spectro-tomography is not a new technique. Its an existing technique with several published examples (the authors include several examples in the references). Since it exists, I disagree with the claim in the abstract that their innovation is "making such [spectrotomography] measurement practically feasible" (line 15). I also disagree with similar statements elsewhere in the manuscript, that "the scanning mode would take impractically long time" (line 41), or "This prohibitively long acquisition time makes spectro-tomography impractical in the scanning mode" (Line 81). The authors should reword claims that the method presented here enabled a jump from something prohibited to something now possible, especially when the examples

presented don’t capture dynamic processes. The manuscript still reports quite a long acquisition time… more on that point later.

Here we specifically refer to scanning-mode spectro-tomography with fluorescence-contrast, which needs to be acquired point-by-point and requires a relatively long dwell-time at each point. To clarify, we make the following changes:

Line 15 of page 1: we deleted “making such measurement practically feasible.”

Line 20 of page 2: “As a result, spectro-tomography experiments are mostly conducted in the full-field mode with absorption-contrast because the scanning mode with fluorescence-contrast would take an impractically long measurement time[2].”

Line 15 of page 3: “This prohibitively long acquisition time makes spectro-tomography in scanning mode very challenging, if not impractical.”

What the manuscript is reporting is a neat original method that makes an existing spectro-imaging technique faster. But there are other reported methods that make this technique faster, like references 14-16. There appear to be no claims of higher spatial or higher energy resolution, or a measurement that is more accurate relative to collecting all angular projections to meet the Crowther criterion, at all energies across an absorption edge. The work presented seeks to achieve an end result of equivalent quality, but faster, by requiring less data and therefore less acquisition time.

That to me is where the impact of the work lies: The reduction in acquisition time. The authors state that their method “reduces the data requirement by two orders of magnitude” (line 14). They also state that they are “reducing the amount of data and measurement time by two orders of magnitude” (line 47). Then, way way at the back of the manuscript (in fact it is the 3rd last sentence of the manuscript) we finally learn that the data acquisition took “about 10 hours” (line 346). In the way it is currently written, its not clear if about 10 hours was for the test object, or the battery material sample, or both combined (I assume it was for one of the two sample measurements). Why is this important detail buried at the very end of the manuscript?

As we stated in the manuscript, there is still a lot of room to improve the method and will require follow-up work. The reviewer is correct, at this stage our focus is on the reduction of data points (or equivalent the time or radiation dose). 10-hour spectro-tomography in scanning mode with fluorescence-contrast is considered very fast. To clarify, we added,

Line 27 of page 2: “We demonstrated that an experiment that would normally take three weeks can be completed in 10 hours.”

Line 1 of page 10: we added “In comparison, it would exceed 500 hours to acquire every sinogram at each energy for the conventional spectro-tomography.”

Now what about a direct comparison of the new results, with other methods mentioned in the second paragraph of the introduction? I found no hard numbers to determine if this method was faster than other methods for the examples presented. The authors should clearly say how long would the measurements have taken by other methods. All I can do is assume it was 2 orders of magnitude larger, so 1000 hours without stoichastic energy sampling? What about the other methods that also speed up the acquisition time? How does this new method compare to other methods needs to be addressed, and the authors must make that comparison easy for the reader. There should be a paragraph clarifying details of the acquisition times, the benefit here, and the benefit by other sparse methods. Without that comparison, I found it difficult to assess if the work belongs in a high impact journal. 10 hours is still quite long in my view. Reducing it down to 1 or 2 hours, that would be something amazing with a big impact. I also suggest stating the acquisition times within the captions of Figs 3 and 4. Also, Fig 3a and Fig 4a could use spatial scale bars.

We have added the reduction of acquisition time in numbers at the beginning and end of the paper for easy comparison. See response above. In the paper, we have indicated that other sparse sampling method would reduce the required number of data points by an order of magnitude, and our method further decreases it by an order of magnitude, reducing it down to a single sinogram. To make it clearer, we made the following changes,

Line 20-24 of page 2: "There have been efforts made to reduce the measurement time by using sparsity in projections, or sparsity in scanned points of individual images. Using a limited number of energy points based on feature selection is another strategy. These methods significantly reduce the amount of required data but still require an order of magnitude more data than a single sinogram."

There is this statement, line 55 - "From one measurement with an acquisition time no greater than that of conventional tomography, we obtained chemical states of Co and elemental distribution of Co and Mn in 3D." I find this statement too vague. What is meant by conventional tomography? What comes to my mind is the single energy full field projection version, where a full tomogram can be collected at a synchrotron beamline in well under one minute. Furthermore, I am skeptical that a single energy tomogram in scanning mode at this resolution level and this field of view would really take 10 hours?

To avoid confusion, we have replaced the usage of "conventional" or "regular" tomography with a single sinogram in the text. The paper focuses on scanning-mode spectro-tomography with fluorescence-contrast, which provides multimodality and high sensitivity, but the downside is the slow speed. We have put some simple math to calculate the required time in the text. If each projection consists of 100x100 pixels and

the dwell time at each point is 20 ms, then one projection requires 200 seconds. Assuming 180 projections for a tomography dataset and we need to repeat the measurement at 56 energy points, then the total acquisition time is 200x180x56 = 2e6 seconds = 560 hours. Our method reduces it to a single sinogram, which is 10-hour long (removing the last factor of 56 in the equation). Long acquisition time is a well-known challenge in scanning x-ray microscopy for spectro-tomography using fluorescence signals, and 10-hour is indeed very fast. Also, the proposed work is just a start, and there is still room for improvement. We do plan to reduce it further to 1-2 hour level to enable dynamics study in the future.

Lastly, I am very surprised the authors didn't mention even one sentence about the other benefit of reducing the acquisition times: Reducing radiation damage artefact by reducing the X-ray dose.

We can't agree more with the reviewer. We added the following sentences in the revision,

Abstract in page one: "…,thereby significantly decreasing acquisition time and radiation dose."

Line 27 in page 2: …,thereby reducing the amount of data, measurement time, and radiation dose by two orders of magnitude."

Reviewer #2

The authors present a study performing spectral CT which samples energy and angle simultaneously, which can be used to develop a spectral CT image with similar sampling time to conventional CT, lowering sampling requirements than current approaches.
The approach relies on,

- Stochastic energy-angle sampling to sample the sinogram
- Iterative tomography and TV regularisation to produce a spectral CT result
- Fitting the energy variation to a set of prior reference spectra as part of the reconstruction

The text is accessible and authors have demonstrated a significant speed up in spectral tomography. The result is novel and will be of interest to the wider imaging and materials science community but there are some concerns on the presentation of approach, particularly it's specific limitations.

Concerns –
Use of prior information
The use of prior information should be explicit. The abstract makes no mention of prior information being intrinsic to the approach. The conclusions suggest general

applicability such as for in-situ experiments, but given all states need to be known it's not clear how this would be achieved.
The author references other spectral sparse approaches, and while they are not as efficient, they are general, and this distinction isn't clear.

We thank the reviewer's positive comments and criticisms. In the discussion session, the required prior information is discussed explicitly. Either known reference spectra (analysis mode) or an additional 2D-XANES measurement (synthesis mode) is required. To clarify, we added in the abstract,

Abstract in page 1: "This is accomplished through sparse and stochastic energy sampling combined with a joint spectral-tomographic reconstruction algorithm that performs the spectrum fitting and 3D reconstruction in a single step."

Line 18 in page 7: "At the current development stage an independent 2D nano-XANES measurement is required as the prior knowledge."

Overfitting and Underfitting Risks
The approach specifically uses prior information, or reference spectra to guide the reconstruction towards the result, with the result having to fit to a mixture of k reference spectra or k species in the sample.
The practical challenge is in knowing the k species present exactly.
If I underestimate – e.g. if there are 3 species and I use 2 references.
If I overestimate – e.g. there might be 5 species and in fact there are only 2.
If a dictionary of references was used?
The weights will still fit and produce an outcome and depending on backgrounds variations and other factors you will get overfitting and spurious results.

Just like any spectrum fitting in XAS, human-in-the-loop with domain knowledge is needed to determine what are the possible states that should be used for fitting. This can be either determined beforehand knowing the reference spectra or by an additional nano-XANES measurement. The domain scientist needs to make the judgement (with combination of other knowledge) whether there is an overfitting or underfitting issue, just like any fitting method. At this development stage, we do not recommend determining number of states from the very sparse spectro-tomography dataset itself (see below discussions). We emphasized this point (see above response). Also, a study of overfitting is added (see below response).

The impact of noise study in supplementary information points to a higher signal to noise requirements, the samples studied are all concentrated, and Figure 3 briefly comments on discrepancies in fitting due to noise.
The propagation of uncertainty in spectro-tomography itself warrants a separate paper

for an in-depth discussion, particularly with high degree of sparsity. We acknowledge that the proposed technique is not near “perfect” or “fully optimized”, and there are many remaining questions to be addressed. Here, we present it as the first demonstration and showed its applications for real samples, opening opportunities that were not available before. We briefly touched on the impact of noise in the supplementary materials but will leave the rigorous error analysis in the future work.

The authors should present a systematic study of over and underfitting or be explicit on the limitation that this has only been shown to work for the exact case where the species in the sample are exactly known and above a signal to noise threshold. i.e. concentrated samples.

We addressed this comment in other responses. Because of the high sparsity, the under/overfitting may not be easily determined from spectro-tomography data itself. However, this can be checked and verified by an independent nano-XANES (the 2D image of the sample with each pixel containing the complete spectrum). Below we show how the algorithm behaves in an overfitting case, where three states are used to fit the spectro-tomography datasets while only two states exist,

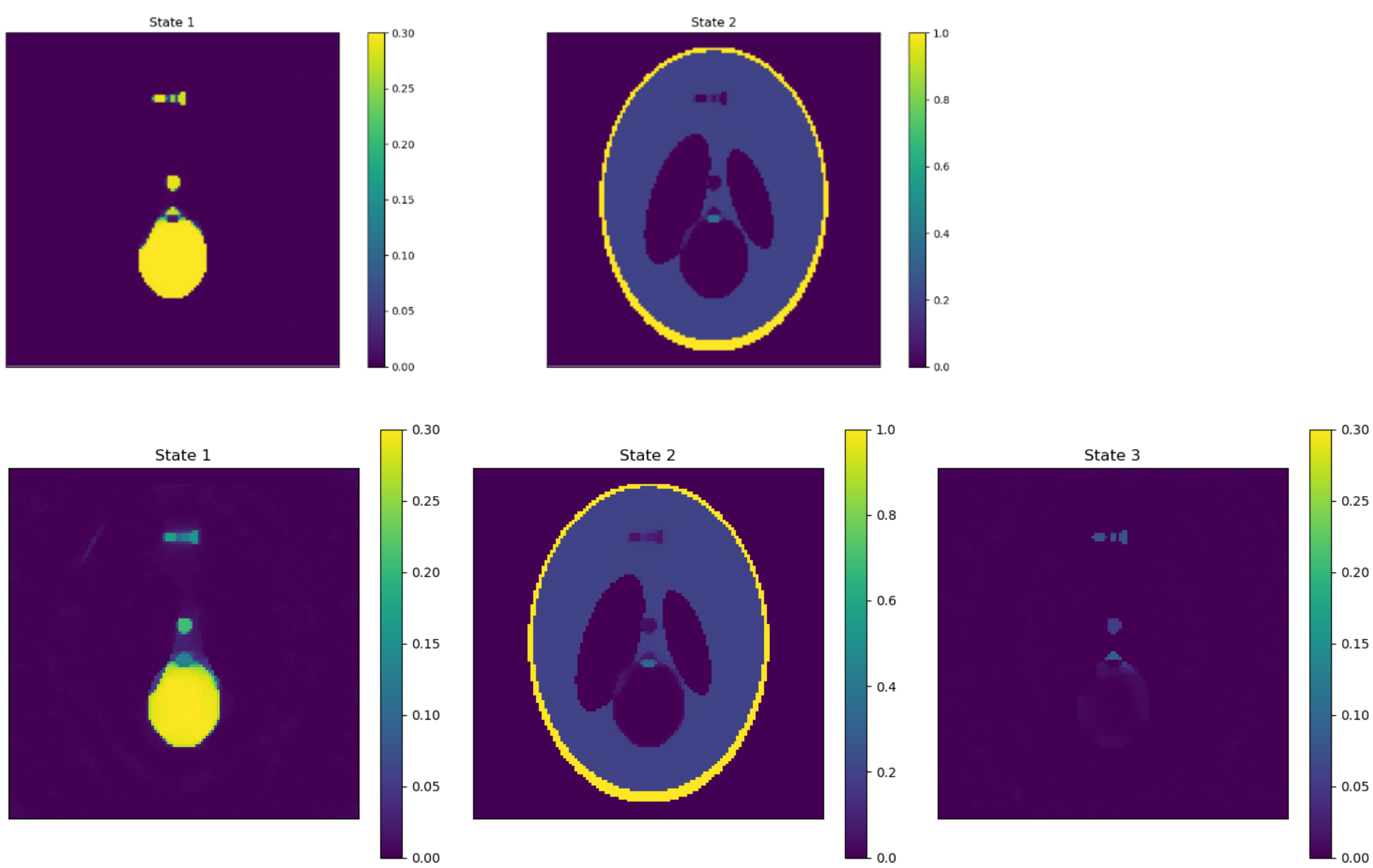

In general, the algorithm still works, but there are more errors in each state, leading to a higher mse. Even for perfect data, state 3, which doesn’t exist, is not completely zero. In theory, it is possible to determine number of states by minimizing the mse error (from 2e-4 with incorrect three states to 4e-5 with correct two states) from spectro-tomography reconstruction process. However, due to imperfections in real data (misalignment, noise, …), this is not so reliable at the current development stage. We

added this discussion to supplementary materials.

Figure 3- The Fe3+ image in particularly has quite different spatial features in the bottom half of the image likely due to overfitting which could be discussed in this context as incorrect conclusions could be drawn depending on the questions being asked of the data.

See the discussion above. For the test sample, the species are completely known. There is no guess on the possible states.

Fe3+ concentration is about 10% of that of Fe0, therefore its distribution has higher uncertainties. We added the explanation in the cap for Fig. 3,

"…, particularly for Fe^{3+} whose concentration is about 10% of that of Fe^{0}".

2D XANES as a Precursor

If k isn't known the authors state that a 2D XANES could be used to work this out, but this approach isn't tested in the work. If the 2D XANES only results in using PCA components and doesn't spectrally unmix the results, the tomography fit to a mixture of PCA components, rather than reference spectra is unlikely to produce clear results, particularly with noise.

The use of 2D XANES should either be validated for the examples where it is acquired or as it's a speculative comment, should be removed.

See our responses in other comments. At the current development stage, to ensure the correctness it is always recommended to do an independent 2D nano-XANES for cross-verification.

Using PCA (or other similar methods) components is not different from using reference spectra. Its use case is for real samples with unknown species, where reference spectra can't be found or there is a trace phase that is not easily seen in 2D nano-XANES (for example a small local cluster in the volume). We need to use PCA (or other similar methods) to determine the number of possible states in such cases.

Below we showed how to use non-negative decomposition to find two base components, perform spectro-tomography reconstruction using these two components, synthesize the spectrum at each pixel, and then fit the synthesized spectrum with two reference spectra for comparison. They are almost identical to analysis mode. We added this to the supplementary materials (Fig. S15), and also updated the code for anyone who wants to test it.

Learned non-negative components from 2D nano-XANES and spectro-tomography reconstruction with the learned components

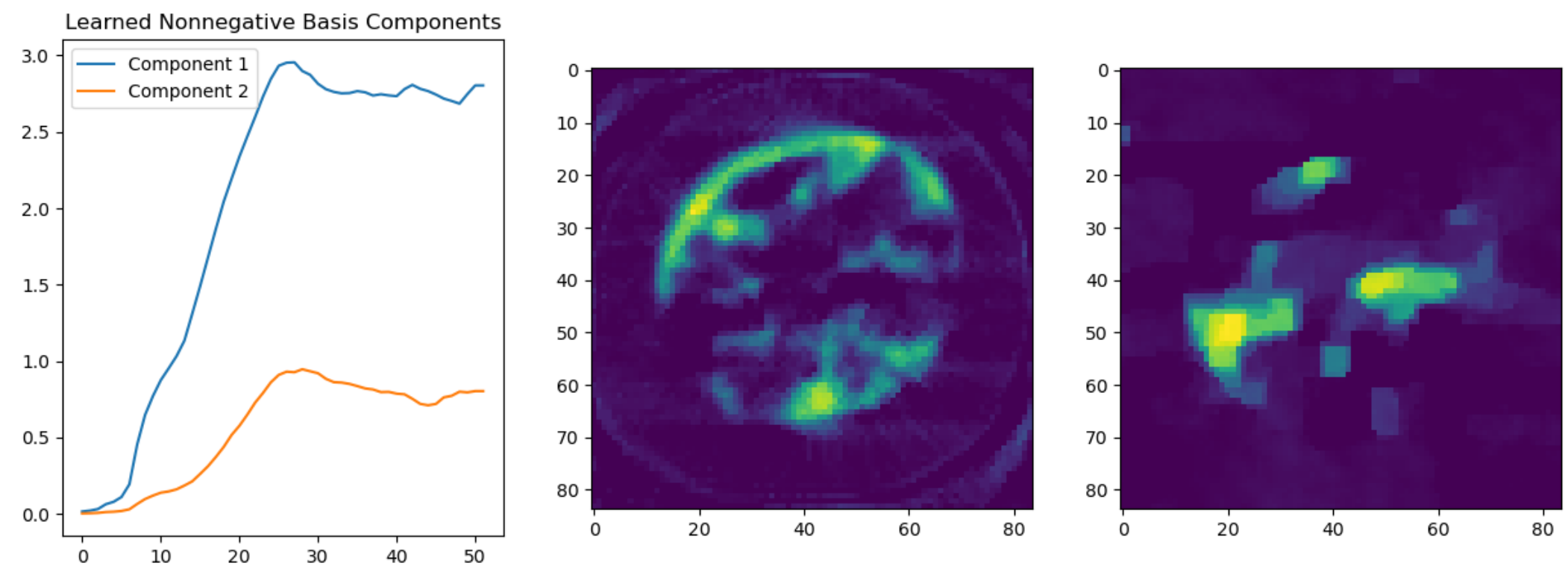

Synthesized spectrum at each pixel by the linear supposition of the base components and fitting results with two known reference spectra

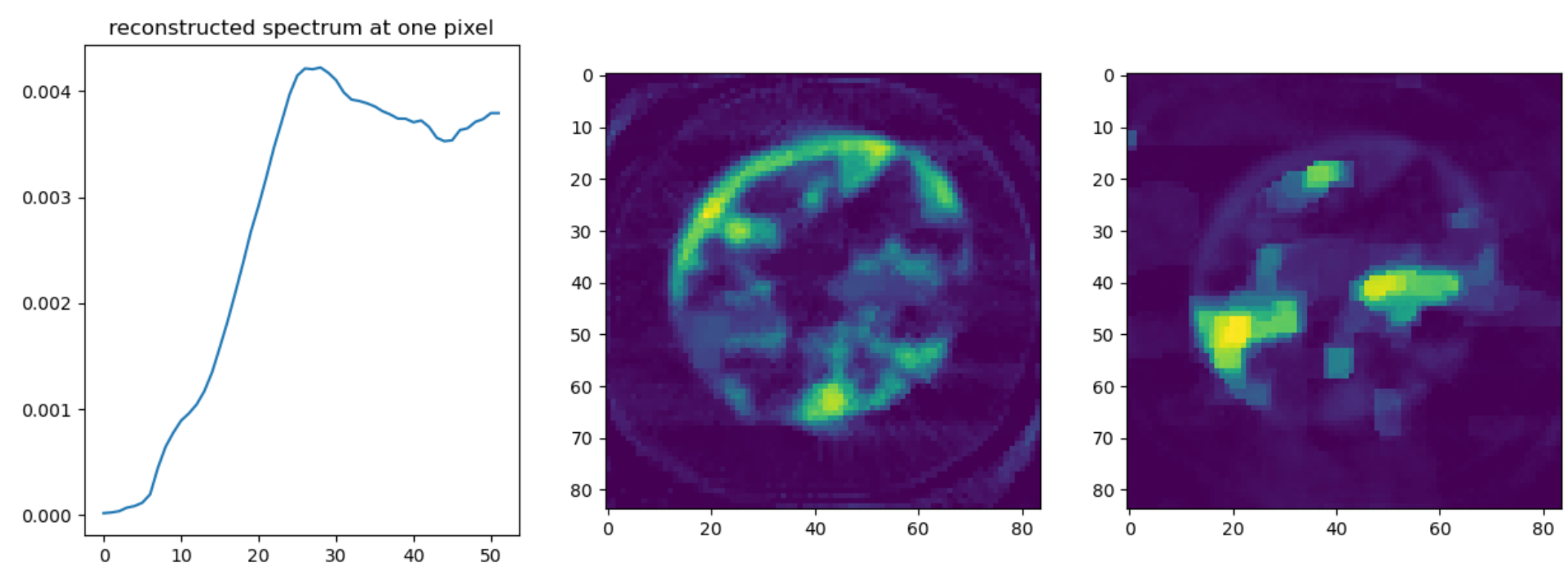

In comparison with direct spectro-tomography reconstruction with two known reference spectra

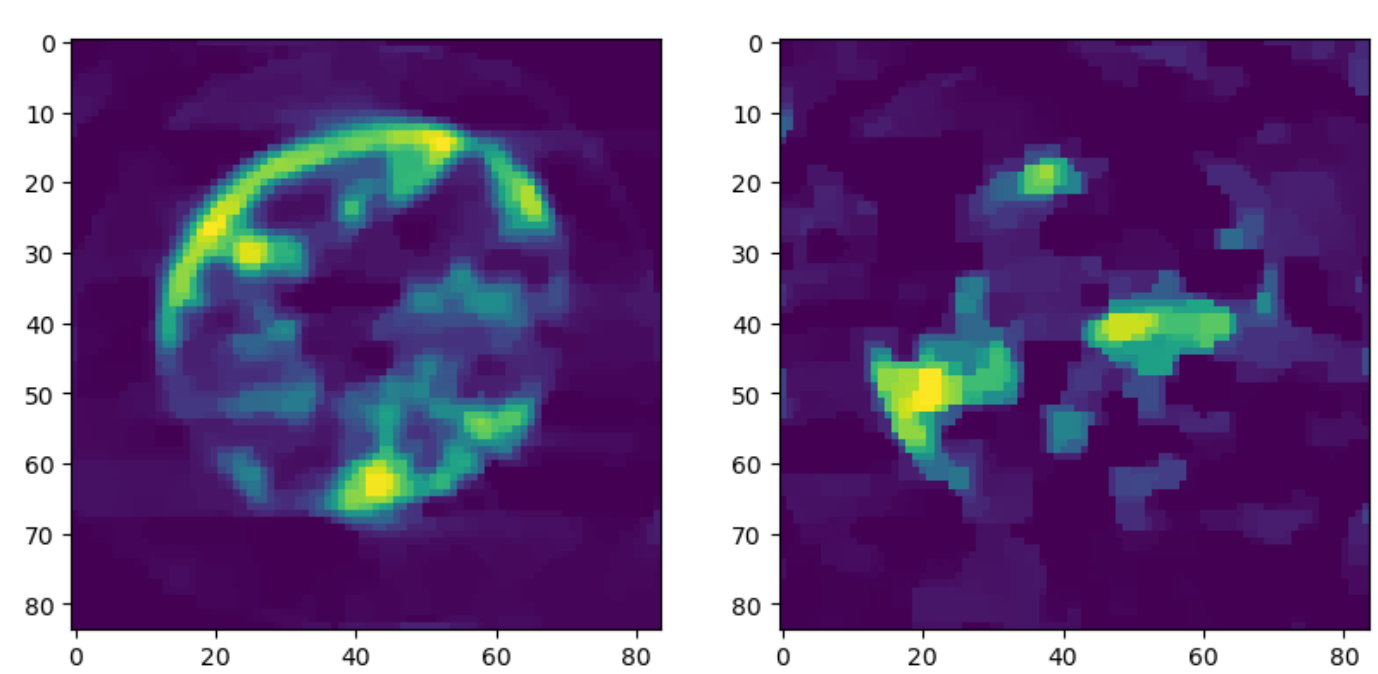

The conclusions mention the expansion to in-situ studies. If the states present haven't been measured previously the outcome would be questionable so it would require a mixture of 2D XANES and known references. This also highlights the need to examine the use of PCA components or to revise the text to be clearer on the limitations.

Thanks for pointing out this. One strategy is to take nano-XANES at the beginning and end of the process and determine all possible states to be used for reconstruction.

Currently, domain scientists need to determine what are possible states (or how many of them). As we show in the simulation it is possible to determine the number of states by minimizing the error (incorrect number of states leads to a larger mse error). However, in real case more work (for example adding more constraints) is needed to make the self-determination robust. We added in the last,

"Another challenge is determining the presented states directly from the data without prior knowledge."

Specific Comments

(Page2 Line 49) claims to directly reconstruct the chemical states where it's a weighted fit from reference data, it's not a direct reconstruction.

If the reviewer refers to this sentence "…, suggesting that we can reconstruct individual states directly from the acquired projection data.", it is a combinational process of fitting and tomography reconstruction. To clarify, we change it to "suggesting that we can reconstruct individual state's spatial distribution in 3D directly from the acquired projection data."

X-ray measurement

Please state how the reference spectra were obtained and processed. Alignment issues are very common in nano-tomography but no mention of pre-processing is made or any impact on this method – please comment

We added the explanation of alignment in the method and how the reference spectra are measured.

Lin3 in page 10: "Projection images were aligned using StagReg [38] and normalized to a fluorescence signal that remained constant across the energy range (Cr for the test sample and Mn for the NMC811 sample, respectively). Reference spectra were obtained from bulk measurements of the same species.

Figure 3

"These results reveal a strong spatial correlation between Cr and Fe0." - should be "demonstrate" or similar, as it's a stainless-steel sample, it's an expected result.

(c) The authors state excellent agreement but the spatial resolution is clearly different. The authors comment in the text on the Crowther criteria, but the use of TV regularization will smooth out results. The resolution is never estimated using FRC or a similar technique and some comment on the resolution or smoothing would be beneficial to understanding the approach.

We changed it to "demonstrate" and replaced "excellent" to "good".

TV does not smooth sharp edges but introduce a well-known painting effect that flattens weak contrast. The weight of TV needs to be tuned for the best result, so a good balance between denoising and feature preservation is achieved.

Spectro-tomography did show a slightly worse resolution than that of fluorescence because there were more unknowns. We added a figure in SI to show the power density spectrum of Fe and Fe0 reconstructions. Fe shows a better (50 nm, half-period) resolution than Fe0 (80 nm, half-period). In the text, we added,

Line 37 in page 5: "Because spectro-tomography involves solving a larger number of unknowns, the state map of Fe^0 exhibits a lower spatial resolution of 80 nm (half-period), compared with the 50 nm resolution achieved in the Fe reconstruction (see Fig. S12)."

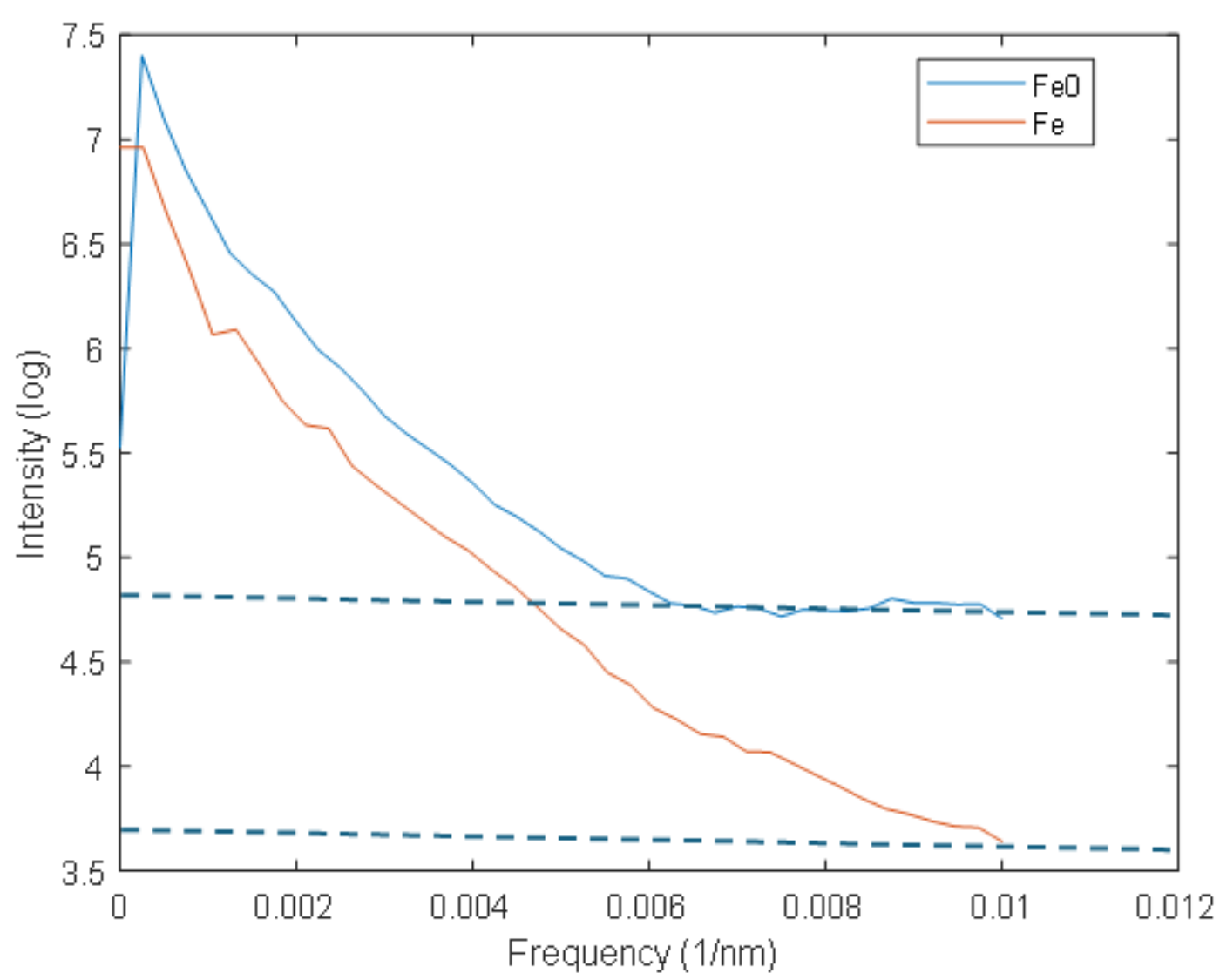

Reviewer #3

(M1) For me the biggest grievance with the manuscript in its current state is the notion that information can be created out of nowhere. To reconstruct volumes of **M** independent coefficients for **M** linear independent spectral response vectors (material components) for each vowel with a volume, at least **M * N**c projections will have to be recorded. The claim that any smart measurement, or algorithm that does not introduce further knowledge and assumptions can recover **M** independent coefficients per voxel from only **N**c projection without loosing resolution or inventing something within each pixels composition is just wrong. The number of measurements (knowns) needs to be equal or larger to the number of reconstructed

unknowns.
The authors claim “achieving perfect reconstruction” from an under-determined problem.
This just can not be.

Response: In our original manuscript, we stated that it was an underdetermined system, which means the number of unknows was larger than the number of knowns. For the original linear equations, the review is correct – there exist infinite number of solutions and you can’t make it work. However, in the proposed method it is turned into an optimization problem, which means we seek a solution that minimizes the cost function defined – this is very different from solving the original linear equations. Mathematically, instead of solving insolvable **Ax = b**, we seek a solution argmin_x ||**Ax -b**||^2 and its stationary point leads to the new linear equation, $\mathbf{A}^T\mathbf{A}x - \mathbf{A}^T\mathbf{b} = 0$, which is solvable because $\mathbf{A}^T\mathbf{A}$ is a positive semi-definite matrix. This is a very common practice in optimization problems. This optimization process always find a solution in the subspace with some implicit constraint, although it is not guaranteed to be the ground truth. By using stochastic sampling that changes the structure of matrix A (see Fig. S1) and adding total variation as the additional constraint (see Fig. S2), we showed that this solution could converge to the ground truth or its vicinity. We confirmed it with simulation and experimental data. That said, there are implicit constraints imposed by the solving algorithm itself (see Fig. S1 for the comparison of different linear solvers) and the regularization term, that enforce the convergence of the solution to a particular one. In the code we added support to other linear solvers one can choose to compare. We have some brief discussion in the supplementary materials, but a more in-depth and rigorous discussion on the mathematical ground requires a full-length article and is out of scope of this paper.

To avoid confusion, we removed the word “perfect”. In Fig. S1, we added the impact of different linear solvers.

(M2) When comparing their stochastic (“random-uniform”) energy sampling approach to the previously published “interlaced” approach, they, in my eyes, make multiple mistakes that happen to give their stochastic approach the edge over the others methods.
The “interlaced” approach was neither meant for not claiming to reconstruct M components from only NC projections. The authors of that study rightfully recorded more than M * NC unique projection angles in total. Here the authors hand the previously published energy sampling approach a problem it was not designed for.

Response: When we compare different sampling schemes including “interlaced”, we need to use the same number of projections for a fair comparison. It is true that the “interlaced” method may not be designed to work with a single sinogram, but that’s the selling point of our method: it uses significantly less number of projections that “interlaced” approach requires to achieve the same satisfactory result.

(M2.1) The problem is not just under-determined, but the problem size and chosen spectral responses are chosen such that (i) the “interlaced” energy sampling is disadvantaged, and (ii) the assumption needed for and thus the strength of the presented joint spectral-tomographic reconstruction is totally disregarded.

Response: We didn't choose intentionally a special spectrum in favor of one sampling scheme over others. The chosen selection of energy points is the one used on daily basis at the beamline for our nano-XANES technique (2D spectral imaging). It is more a convenience argument from an experiment point of view, rather than a theoretical consideration.
Because of the computational complexity of the joint spectral-tomography reconstruction, it is computation-heavy. At the current stage, our focus is to demonstrate the proof of concept, not the speed. There are many ways to speed up the computation, e. g., using gradient based methods such as accelerated proximal gradient and automatic differentiation where computation can be done on GPUs. We added a discussion on the computation cost and future improvement plan.

We added in the discussion,
Line 18 in page 7: "Because of the iterative nature of the method, the computation time can be up to a few minutes for a slice with a few hundred pixels. The main bottleneck is the linear solver used in ADMM. A significant speedup on GPUs can be achieved using gradient-based methods such as accelerated proximal gradient [29] or automatic differentiation [30]"

(M2.2) With that I mean that the presented algorithm hinges on knowing the spectral responses for all components within the sample, which are all known prior to the reconstruction of the data. The authors explain that these spectral responses could be obtained from a fully energy sampled set of projections, taken prior to the tomogram, using PCA (This only holds true if in the projection in question the components appear in different projected pixels with linear independent coefficients. Thus it could fail simply by an unlucky sample structure and choice of projection angle).

Response: When we take a fully energy-sampled nano-XANES at one projection angle, since x-ray sees through the object (the object size is limited to a few microns for nano-tomography) and we do a 2D imaging over the entire sample, by fitting the spectrum at the single-pixel level all possible states would be revealed. We don't see how a sample structure or choice of projection angle would matter.

(M2.3) The authors did not at all take into account that, knowing the spectral response of all material components within the sample, they could have selected a set of energy point where the multiple spectra show the largest differences to each other. Instead of measuring only energies that allow to separate the various components, the spectra contain many energy points were the spectral response from the materials is very similar or shows a smaller difference than at other energy points.

Response: It is very true, and the author completely agrees with the reviewer. That's why we stated at the end "Optimizing the selection of sampled energy points could also help reduce uncertainties" as the future direction for improvement. Also, in Fig. S5 the impact of orthogonality of the spectra has been demonstrated in a simple way. For practical applications, optimization strategy on energy selection needs to be developed, and that will be one of the remaining tasks. In the current work, as the first demonstration we want to show that the method yields a satisfactory result with minimum number of measurements.

(M2.4) One could have picked a small set of energies with the most orthogonal spectral response vectors. Instead many adjacent energy points have very similar spectral responses as their neighboring energy points. This becomes very clear when plotting the difference of the two spectra used in the simulation: All energy points where the difference of the spectra is close to zero will show little to no contrast between the two materials. There is no reason to measure those in the

first place. Looking at the remaining energy points it becomes clear that there are stretches of adjacent energy points where the contrast between the, in this case, two materials is similar. Measuring those groups adjacent energy points with similar responses at adjacent projection angles means there is no way to differentiate well between the two material components for those angles. That is why the interlaced energy sampling strategy is at a disadvantage here to the "uniform-random" strategy, where there is a higher chance to jump between points energy that are to each other linear independent in their spectral response, meaning it is more likely that the two material components can be separated for those adjacent projection angles (this arc of angles)

Response: Again, we choose the same energy points used for 2D nano-XANES due to experimental convenience, since everything has been set at the beamline, and it is easy to make a one-to-one comparison. As Fig. S5 demonstrates, least correlated spectra are preferred, and stochastic energy sampling gives a simple way to achieve that, which is one of key points of this paper. We never claim the method demonstrated is the best among all possible scenarios. In the paper, we acknowledged in the end that follow-up work on energy selection is needed for future improvement. We addressed in detail the reviewer's comments in M2.5.

(M2.5) I argue (and I tested) that just randomizing the order of the used 52 energy points, but keeping that same order within each interlaced arc does have the same positive effect. Additionally it does still come with a homogeneous angular sampling for each energy, avoiding that by chance nearby projection angles are measured by the same energy, resulting in an unavoidable under-sampling of those energies from different projection angles. The best results I achieved by combining a sampling strategy that gives constant angular sampling within one energy and only measuring energy points from the known spectra that allow the best possible separation of the two material components.
Below my results using the authors simulation and reconstruction code: From left to right I varied the number of energy points of the available spectra I ended up using. On the far left, the most extreme case of just selecting two energy points to differentiate the two material compoents.

We greatly appreciated the time and efforts the reviewer spent and brought this observation to our attention. We added Table I to quantify the mean square error under various conditions, including what the review proposed. Indeed, interlaced with randomized spectra shows a slightly better result from 20 trials with less outliers. We have made changes accordingly to reflect this observation and added a new Table I quantifying the error to the ground truth. Again, we want to emphasize that one important pilar of the proposed method is to randomize the spectra (stochastic energy sampling), a simple and efficient way to achieve a good spectro-tomography reconstruction when it is combined with the algorithm development. Improvement is seen in all cases when the order of energy points is randomized, as shown in Table 1.

Table I Comparison of the mean square error for different angle–energy combination strategies using both regular and randomized spectra. For the randomized spectra, 20 trials were conducted to compute the mean and standard deviation, capturing the uncertainty inherent in the random process.

	Segmented	Interlaced	Stochastic
Regular spectra	1.65E-2	7.23E-3	2.89E-5 (1.31E-5)
Randomized spectra	3.61E-3 (2.35E-4)	1.80E-5(8.34E-6)	

We added,

Line 35 in page 4: "Note that the key is stochastic energy sampling. When the randomized spectra were applied to other sampling strategies, performance improvements were also observed. Table I reports the mean square error (MSE) for the different cases to illustrate this effect. The interlaced sampling achieved an MSE comparable to – if not better than - that of the stochastic angle–energy combination, indicating that their difference diminishes in such a case."

(M2.6) In all cases I used the (to few) 181 projection angles (as the authors) and only used values within the circular disk (not the corners of the reconstructed array) to evaluate the quality of the construction.
No matter how many energies were used, mixing the initial order of the energy points and going through them again and again (green) does result in equal or lower errors than just rolling the dice for each angle (blue). Measuring fewer and fewer unnecessary energy points does reduce the error achieved by simple "interlaced" energy sampling (orange).
The results become even better (for all three strategies) if a sufficient number of projection angles (> 2*Nc in this case) is recorded.
While I do not believe that the authors chose the simulation parameters (128x128 pixel objects, 181 angles, 52 energy points) on purpose to have their method come out on top, this choice is the reason their algorithm worked better in this case. I want to stress that it was a sub-optimal choice to "run the experiment"

See response to M2.5. To address the reviewer's comment on "insufficient number of projections". The whole point of paper is to use a sparse dataset to achieve a satisfactory result, in which case others fail. Of course, adding more data always helps to improve the result, and the difference between methods would diminish, but that's not the goal of this work.

(m.1) All figures in the manuscript are from rather low number of pixel data. That is the nature of the simulated and recorded data. And that is fine. But having not having the default sub-pixel interpolation of matplotlib.pyploy switched off does introduce a smoothness in the data that is just not there and might trick some readers. The authors should use the optional argument *interpolation='None'* in all their figures to show square pixels of homogeneous value.

We turned off interpolation wherever possible and updated the figures.

(m.2) In and around Figure 2 the authors present a simulation of a two-compound test sample. In line
139 they claim achieving "perfect" reconstruction. While the word "perfect" in itself can not supported (see above), this could not possibly be seen from the figure as it is. I suggest that the authors plot the difference between the ground truth and their
reconstructions in a diverging colormap. Additionally they should find some error metric to report the non-zero difference between GT and reconstructions with some quantitative number. Running the authors unaltered code and plotting the difference between GT and reconstructions as suggested, it becomes obvious that there is a non-zero difference (examples shown within the next comment).

We removed "perfect" and added mse as the quantifying metric in Table I.

(m.3) When presenting a new method based around a random number generator it is good practice to study if the seed of for the random number generator can have any impact on the quality or even consistency of the obtained result when everything else remains unchanged.
Having access to the code of the shown simulations I just ran the simulation multiple times with different seeds for the random number generator and found the quality of the reconstruction to vary. See below a selection of very few of the different results I obtained for different seeds. Having tried many seeds, I can say that overall many of them seem to obtain results of similar

quality for the simulation at hand. But there is also a decent chance that a randomly picked seed fixes a energy to angle selection that results in multiple times larger errors.
As the selection of energies to each angle happens before the data taking, there is no way of reducing the impact of a potential bad selection purely by means of reconstructing the data differently once it is taken.
This behavior needs to be honestly discussed in detail

Thanks for pointing out this, and we added Table I to capture the fluctuation of the random process. We also added the following sentence at the end,

Line 30 in page 7: "Optimizing the selection of sampled energy points could also help reduce uncertainties 30, and the same holds for refining the randomization process. "

(m.4) The addition of TV into their minimizer to be able to deal with noisy data was well thought out and important. But TV is "just" enforcing a certain smoothness within the obtained reconstruction / penalizing strong gradients. I would like to have seen a discussion of other options, including some that take into account the nature of the noise in the data (Poisson noise).

Indeed, we had that discussion at the end, indicating that more advanced regularizer, such as AI-enabled one, can further improve the result. We cited our work that incorporates a CNN network as a smart regularizer into the iterative process to solve limited-angle and sparse projection tomography, another underdetermined problem. It is now published, and we updated the reference. Certainly, there is a lot of room for improvement.

For Poisson noise, the main difference will be the likelihood function (the fidelity term) used. Poisson likelihood function may yield better result for low counts but require more computation. At current stage, we limit ourself to Gaussian assuming white noise. We added,

Line 14 in page 8: "The first term corresponds to a Gaussian likelihood function, which assumes white noise in the data."

(m.5) The push towards joint reconstruction algorithms within the community is something I see as a very positive thing. Explaining problems as a whole and solving them as a whole as well, allows to use every bit of information wherever it might be. The authors should discuss the computational need / requirements and most importantly the scaling of their algorithm with the problem size. While playing with the authors code myself, I noticed that the minimizer does require a considerable amount of time to iterate through problems of very small size. How does the reconstruction time scale when the problem size doubles, quadruples, ect?

We thank the reviewer's encouragement. The computation is intensive, a price you pay for joint reconstruction. However, there are many ways to speed it up, utilizing the power offered by GPUs.

We added some discussion about the possible route for future improvement. This is very similar to how iterative tomography algorithm can be improved by GPUs and is very doable (see our other responses). We intend to make it a routine technique at the beamline and will continue to update the code, so it will become more practical in terms of computation time.

Dear Editor,

We thank the referee for the additional comments and have provided a point-by-point response to the best of our ability. Our responses (in red) and revisions (in green) are listed below.

Reviewer #2

My intent is not to unnecessarily extend the manuscript, but rather to ensure that the core assumptions and limitations are clearly visible to the reader, not only explained in the rebuttal.

Abstract (Page 1)

Revised text currently states:

"This is accomplished through sparse and stochastic energy sampling combined with a joint spectral-tomographic reconstruction algorithm that performs the spectrum fitting and 3D reconstruction in a single step."

Reviewer request:

The abstract should be more descriptive and explicit about the reliance on prior chemical knowledge. For example:

"…performs the fitting to known chemical signatures present in the sample and 3D reconstruction in a single step."

This small change would immediately clarify the scope of applicability at the highest level of the paper, otherwise explicit statements on exact states are only in the supplementary information.

As suggested by the referee, we have changed the sentence to,

"This is accomplished through sparse and stochastic energy sampling combined with a joint spectral-tomographic reconstruction algorithm that performs the spectrum fitting with prior knowledge of the known reference spectra and 3D reconstruction in a single step."

Overfitting and Underfitting

I thank the authors for adding the new figures in Supplementary Figure S3 however, the caption and accompanying text remain misleading.

Supplementary Figure S3 Caption

Current caption states:

“(b) Spectro-tomography reconstruction with three states, while only two states exist. It leads to a bigger error as compared with the correct two-state reconstruction.”

Reviewer comment:

The issue is not simply a larger error. The more important consequence is the potential misidentification of a non-existent chemical state and distortion of the spatial distribution of the true states. This is a qualitatively different and more serious limitation than a generic increase in reconstruction error.

I recommend revising the caption and/or supplementary text to explicitly reflect this, for example by noting that overfitting can introduce spurious chemical states that do not exist in the sample.

Supplementary description - Impact of hyperparameters and number of states

The revised manuscript currently states:

“Proper tuning and the correct selection of reference spectra is essential for achieving optimal results, especially when the data is noisy.”

Reviewer request:

Please make this more explicit. Suggested revision:

“Proper tuning and the exact selection of reference spectra that are known to be present in the sample under investigation is essential for achieving optimal results, especially when the data is noisy.”

This clarification is important, as the risk is not merely suboptimal performance, but incorrect chemical interpretation.

There may be some misunderstanding here. What we mean is that overfitting can lead to a larger MSE, which can therefore be used to rule out an incorrect third state. In principle, one could adopt a strategy that searches over all possible combinations of states and selects the one that yields the lowest MSE.

To clarify this point, we have added the following explanation to the Supplementary Material, as suggested by the referee:

“In principle, we can develop a strategy that tests all possible combinations of states and selects the one that yields the smallest error—a data-driven selection process. However, due to imperfections in the data, multiple combinations can yield similar mse and an incorrect overfitting may occur. Therefore, additional constraints—such as prior electrochemical knowledge to narrow the selection—are needed to determine the reference spectra. In Fig. S3, we present several examples illustrating the impact of these parameters. At current stage, proper tuning and correct selection of reference

spectra known to be present in the sample under investigation are essential for achieving optimal results, especially when the data are noisy."

In Fig. S3 caption, we added

"Therefore, in principle we can use minimized mse to guide the selection of states – a data driven process. However, imperfection in the data may lead to an overfitting problem if no prior knowledge is employed to constrain the selection."

On the topic of overfitting the authors responded -

Just like any spectrum fitting in XAS, human-in-the-loop with domain knowledge is needed to determine what are the possible states that should be used for fitting. This can be either determined beforehand knowing the reference spectra or by an additional nano-XANES measurement. The domain scientist needs to make the judgement (with combination of other knowledge) whether there is an overfitting or underfitting issue, just like any fitting method. At this development stage, we do not recommend determining number of states from the very sparse spectro-tomography dataset itself (see below discussions). We emphasized this point (see above response). Also, a study of overfitting is added (see below response).

And later comment

"In general, the algorithm still works, but there are more errors in each state, leading to a higher mse. Even for perfect data, state 3, which doesn't exist, is not completely zero. In theory, it is possible to determine number of states by minimizing the mse error (from 2e-4 with incorrect three states to 4e-5 with correct two states) from spectro-tomography reconstruction process. However, due to imperfections in real data (misalignment, noise, …), this is not so reliable at the current development stage."

But only the 1 line statement above was made and the potential pit falls still feel unclear in the text. Adding some of the rebuttal text would improve overall clarity.

To make this clearer (as suggested by the reviewer), we added in the main text,

"As current limitations, proper tuning and correct selection of reference spectra known to be present in the sample under investigation are essential for achieving optimal results, especially when the data are noisy."

Reviewer #2 (Remarks on code availability):

Code presentation is poor and would benefit from following a standard such as JOSS on presentation.

There are no comments in the code or notebooks and no doc strings.

There is no README, requirements.txt or environment.yml

This is very much a demonstrator code, no a re-usable resource.

We apology for the insufficient documentation and lack of REAME for the code, and our oversight not addressing them in the previous revision. We have updated the code at github accordingly.

Reviewr#3

Reviewer #3 (Remarks to the Author):

I thank the authors for adjusting their manuscript with regard to the other reviewers and my comments.

However, it feels that in regards to most concerns raised by the reviewers the authors looked for the smallest possible change that might appease us or simply not adapt anything.

We respectfully disagree. The revisions since Version 1 have been substantial, and all changes have been clearly highlighted. We have made every effort to address the concerns raised by the reviewers.

We sincerely appreciate the reviewer's time and effort. However, there are several points on which we do not agree. It is not fair to characterize our revisions as minimal simply because our conclusions differ from the reviewer's.

We will continue to address all comments to the best of our ability. Where we disagree, we provide clear explanations and justification.

I want to be very clear that I believe the presented work is worth publishing. But major changes to the manuscript will have to happen before that. From my side, further changes do not require any additional experiments, data taking or simulations. The already obtained results just need to be discussed more honestly.

My suggestion to the editor remains "major revisions".

I will further elaborate on my thoughts in detail, focusing on my previous comments and how they were addressed by the authors.

(M1) My concern remains: The authors do not discuss that their method creates information out of nothing. Yes, they solve a minimizing problem. But the whole manuscripts reads as if the same number of spatial resolution elements and spectral components per spatial resolution element can be recovered from way fewer measurements. In fact, sometimes not even enough measurements to properly reconstruct a single spectral component in the tomographic plane.

There is only one place place in the manuscript where this shimmers through: In the added text around line 197, showing that solving for multiple from spectral components within a tomogram reduces the resolution in the obtained tomographic reconstruction → lowering the number of spatial resolution elements for the sake of recovering multiple spectral components. Effectively this shows that the number of unknowns to be solved for from a fixed number of knowns can not magically increase; just shifted around across dimensions.

We have explained this point in our previous response; here we reiterate it with a simple and concrete example.

Once the original underdetermined linear system is reformulated as an optimization problem and a specific solver is chosen, an implicit, algorithm-dependent constraint is introduced. The algorithm effectively searches within a subset of the full solution space and returns a solution that satisfies this constraint. We illustrated this behavior in Fig. S1, showing that different solvers can lead to different results.

Incorporating total variation (TV) into the optimization imposes an additional constraint: the solution is encouraged to be spatially smooth. In practice, this favors solutions that are piecewise constant, with sparse gradients and possibly sharp edges. In doing so, a single solution is selected from an otherwise infinite set of feasible solutions.

To illustrate this more clearly, consider the following simple example:

$$Ax = b, \begin{bmatrix} 1 & 1 & 0 \\ 0 & 1 & 1 \end{bmatrix} \begin{bmatrix} x1 \\ x2 \\ x3 \end{bmatrix} = \begin{bmatrix} 1 \\ 1 \end{bmatrix}$$

The solution is

$$\begin{bmatrix} 1-t \\ t \\ 1-t \end{bmatrix}$$

Therefore, there are an infinite number of solutions since t can be any value.

When we turn it into an optimization problem,

$$\underset{x}{\operatorname{argmin}} \|Ax - b\|_2^2$$

In this simple case, we can use pseudo-inverse to solve it, $x = (A^T A)^{-1} A^T b$, and arrive at

$$x = \begin{bmatrix} x1 \\ x2 \\ x3 \end{bmatrix} = \begin{bmatrix} 1/3 \\ 2/3 \\ 1/3 \end{bmatrix}$$

It is easy to verify that this is the solution minimizing $\|x\|_2^2$. It selects a specific solution

If we add total variation as the regularizer with enough weight, then the unique solution is changed to

$$\begin{bmatrix} x1 \\ x2 \\ x3 \end{bmatrix} = \begin{bmatrix} 1/2 \\ 1/2 \\ 1/2 \end{bmatrix}$$

Therefore, depending on the properties we wish to impose on the solution, different solutions may be obtained. While this introduces some degree of subjectivity, it is no different from other optimization problems that involve hyperparameter choices. We have acknowledged this limitation in the main text by adding the following statement:

"As current limitations, proper tuning and correct selection of reference spectra known to be present in the sample under investigation are essential for achieving optimal results, especially when the data are noisy."

In a sense, our approach can be viewed as a physics-informed estimation of missing information using prior knowledge. It is true that the data are insufficient in the strict sense that the number of unknowns exceeds the number of measurements. However, a solution close to the ground truth can still be obtained by incorporating a forward model and appropriate constraints. We do not create information from nothing, nor do we make such a claim.

What we present here is not fundamentally different from established iterative tomography reconstruction methods such as MLEM, FISTA, and other forward-model-based approaches. When data are undersampled, iterative methods are well known to significantly outperform direct methods (such as FBP), because direct methods rely solely on inverting the measured data and do not incorporate prior knowledge. The Crowther criterion is derived for direct reconstruction methods; iterative methods are generally more tolerant of undersampling. This is widely recognized in the field.

As clearly stated in the manuscript, the key reason our method works is the use of prior knowledge to achieve dimensionality reduction. Specifically, we reduce the number of unknowns at each voxel from the number of energy points to the number of states. If the spectral variation were arbitrary, the method would not work, and sufficient data would need to be collected to resolve all unknowns. This limitation is explicitly acknowledged.

With the increased number of unknowns (multiple states versus a single state), the spatial resolution is slightly degraded compared to conventional single-state tomography. We have acknowledged this in the manuscript and provided a comparison. This limitation could be mitigated by incorporating additional prior spatial knowledge—for example, through an AI-enabled regularizer, as demonstrated in our recent work (Zhao et al., *npj Computational Materials* 11, 240, 2025). Further development along these lines will be the subject of future work.

Regarding the last comment, "Effectively this shows that the number of unknowns to be solved for from a fixed number of knowns cannot magically increase; just shifted around across dimensions.", The novelty of this work lies in demonstrating, for the first time, the ability to achieve 3D multistate chemical imaging from a single sinogram. It addresses the question of **whether this is feasible**, rather than **how far it can be optimized**. Compared with conventional single-state tomography, the resolution is indeed lower, as the number of unknowns increases while the amount of data remains the same. There is certainly room for improvement, and we have discussed several possible pathways.

To clarify this point, we have added the following statement to the Introduction:

"With the prior knowledge of chemical states presented in the sample, we reduce the number of unknown spectral variations with energy down to the number of states, thereby requiring much less data points. A full measurement will be still needed if this dimension reduction cannot be done."

(M2) The authors did not address that they misrepresent someone else work that they cite, by doing something that is similar but inherently wrong and specifically avoided by the original publication. The authors should highlight that they use and adapted version of the "interlaced" energy sampling approach (see citation) but with too few knowns / measurements contrary to the original work.

Again this feels like the authors try to dance around the apparent problem that the presented approach tries to work with a way under-determent problem.

We respectfully disagree and believe the reviewer still misunderstands the intended application scenario of this work. Our study concerns **scanning fluorescence X-ray microscopy**, for which the originally cited "interlaced" method would still require an acquisition time that is impractically long. We do not avoid adopting that method because it is inferior, but because it requires substantially more acquisition time. If sufficient data can be collected quickly enough, then simply satisfying the Crowther criterion and applying direct reconstruction would already be sufficient.

We also disagree with the suggestion that we are trying to evade the issue. If that were the case, we would not have included **Table I**, which benchmarks performance across different scenarios in the most direct and faithful way. We have clearly stated that the

randomized spectrum is the key factor. Improvement is observed in all scenarios when a randomized spectrum is used. Among the four simulated cases compared, the interlaced approach combined with a randomized spectrum does indeed produce the smallest MSE, although the difference relative to the stochastic angle-energy approach is negligible. We have acknowledged this point, but it does not alter any of our conclusions.

To clarify this point and make it clearer that interlaced-randomized spectrum indeed achieved the smallest MSE, we revised the text to:

"To ensure a fair comparison, the randomized spectrum was also applied to other sampling strategies. Table I reports the mean squared error (MSE) for the different cases, and improvements are observed across all of them. Notably, interlaced sampling, when combined with a randomized spectrum, achieves an average MSE that is slightly lower than that of the stochastic angle–energy combination. This indicates that stochastic energy sampling plays a key role in improving reconstruction quality. In this case, the latter two sampling strategies still perform noticeably better than the segmented, although the performance gap is reduced.
In the following discussion, we use the stochastic angle–energy combination for all simulations and experimental measurements. Note that the interlaced–randomized spectrum scheme could yield results that are equally good, if not slightly better."

(M2.1) If the number of spectral points and sub-Crowther-limit angles is a commonly used approach at the beamline, then this should be pointed out to the reader. It should be discussed in detail that these numbers are not sufficient for the tomographic problem and not optimal for the decoupling of spectral components (of that many spatial resolution elements).

Forward-model-based iterative algorithms can greatly reduce the artifacts that appear in reconstructions obtained by direct methods when the data are undersampled, that is, when the Crowther criterion is not satisfied. This is because the Crowther criterion considers only the sampling of the data itself. It does not account for prior knowledge, such as additional constraints on the solution, the physical model, or the imaging geometry. This is well understood in the tomography community.

We have shared everything, from the simulation code to the experimental data, so that every figure and numerical result in this work can be reproduced and verified by anyone.

At present, we always use iterative reconstruction methods—most often FISTA—to obtain the best reconstruction, although a direct-method reconstruction (such as Gridrec) is also generated for comparison. In all tomography papers published from the beamline, the experimental conditions and the reconstruction algorithm used have been clearly stated.

(M2.2) An unlucky choice of sample and projection angle(s) for the 2D XANES scans could, due projection, end up with the respective spectral components to contribute with the same relative fractions in each (projected) pixel. While it is unlikely, it is not impossible. In fact there are an infinite, be it artificial, samples that, when projected along the right angle, show the same relative mixing of spectral components in all pixels. One example would be a chess board like pattern projected along either of the sides of the board. A XANES scan of such an, in the real world unlikely, projection could not decouple the multiple spectral components, as the projected response can be explained with one single spectral base vector.

If we understand the reviewer's argument correctly, the concern is a theoretically possible but practically extremely uncommon case: although two spectral components are present, their projected sum could be identical at every pixel. First, this scenario is highly unlikely in practice. Second, even if it were to occur, it would not present a major problem, because the situation would then be no different from a bulk XANES measurement.

When only a single effective spectrum is observed, purely data-driven analysis methods such as PCA are not applicable. In that case, the scientist must rely on domain knowledge to determine the possible underlying states. Whatever procedure would be used for a bulk XANES measurement would apply equally here. This is analogous to the case of undersampled data: the data alone may be insufficient, but domain knowledge can still be incorporated. In the extreme case where neither informative data nor relevant prior knowledge is available, our method would not work, because no meaningful dimensionality reduction could be performed.

To make this point clearer, we added the following discussion:

"If neither the reference spectra nor the base components are available, the proposed method will not work, because the number of unknown spectral points can't be reduced to the number of states."

(M2.3 and M2.4) The word "orthogonal", or derivatives (like linear "independent"), does not appear anywhere in the article or the supplementary. Again it seems that the authors try to avoid the shortcomings of the presented approach.

In the Supplementary Material, we include a dedicated paragraph discussing the impact of spectral correlation, along with a comparison shown in Fig. S5. We intentionally avoided the term "orthogonal" and instead used "least correlated," as it is more accurate. Because the spectra are non-negative, they cannot be orthogonal to one another.

Our method does not require the spectra to be orthogonal; it only requires that they are not linearly dependent—that is, one spectrum cannot be expressed as a scaled version of another. Under this condition, the spectra can, in principle, be reconstructed. Our results show that the less correlated the spectra are, the easier it is to recover a solution close to the ground truth.

We have shared all materials necessary to reproduce every figure and numerical result in the paper. Therefore, it is not reasonable to suggest that we have attempted to obscure the limitations of the method; all relevant details are fully disclosed.

Like any method, ours has both strengths and limitations, as well as a specific range of applicability. It is designed for scenarios in which only a single sinogram can be acquired for multistate tomography. If sufficient sampling data are available to satisfy the Crowther criterion, iterative methods are not required, and direct approaches such as FBP or Fourier-based reconstruction perform well. In practice, however, experimental constraints often limit the number of measurements, resulting in significant undersampling. In such cases, direct methods fail to produce meaningful results.

If a method relies on some sort of prior knowledge (the spectral response base vectors of all components) it is well worth discussing that some photon energies do not give any (additional) information and thus should not be part of the energies used to measure. Choosing a subset of energies that the resulting spectra are as “linear independent as possible” is not something that can be brushed off for a future paper. It is known already know and might as well be reported as a best practice within this manuscript.

Our paper focuses on the question of **whether this can be done**, rather than on **how far it can be optimized**. As a first attempt to perform multistate tomography from a single sinogram, our primary goal is to establish feasibility. There is certainly room for further improvement, and we have already outlined several possible directions for future development, including optimization of the selected energy points.

We also explained in our previous response why we chose a spectrum that includes several pre-edge points. This choice was made largely for experimental convenience in a 2D XANES setup. In addition, it allows us to directly compare the reconstructed spectrum with the experimentally measured one.

To make this point clearer, we added the following statement:

“Pre-edge energy points with low intensity have less impact on the 3D reconstruction, and an optimization of energy-point selection can further improve the performance. We chose the same energy points with that of a nano-XANES measurement at the beamline for experimental convenience and a subsequent comparison of the reconstructed spectra. “

(M2.6) I can grasp that it is not the "goal" of this work to take sufficient data. But then it must be clearly stated and discussed. This concern of mine is a repeat of the previous points. The whole manuscript seems to explicitly try to hide that not enough information is recorded to have both the full number of spatial resolution elements and spectral components per spatial resolution element.

In the Introduction, we clearly state that, for fluorescence-based scanning X-ray microscopy, acquisition speed is the main bottleneck. As a result, it is impractical to collect more than a single sinogram for multistate tomography. In the Introduction, we wrote:

"However, for three-dimensional (3D) chemical imaging using spectro-tomography, combining energy scans with angular projections results in an excessive number of tomographic measurements. Conventionally, spectro-tomography requires a full tomography dataset at each energy point, i.e., one sinogram per energy. Considering 50 – 100 energy points for a spectrum, a tomographic measurement must be finished within a few minutes to limit the total acquisition time in a day for practical applications. As a result, spectro-tomography experiments are mostly conducted in the full-field mode with absorption-contrast [12,13] because the scanning mode with fluorescence-contrast would take an impractically long measurement time of weeks[2]."

We also have indicated the reason why the method works (dimension reduction),

"By this manner we significantly reduce the number of unknowns in the fourth dimension, from the number of energy points to the number of chemical states. From a mathematical point of view, this means much less data is needed to find a solution."

Our method is intended for the extreme case in which no more than a single sinogram can be acquired within a reasonable time frame (approximately one day). However, when additional data can be collected—for example, in full-field spectro-tomography—our method is not necessary. We have never claimed that this method should be used in every situation. Its target application is specifically X-ray scanning microscopy in fluorescence mode, and this has been clearly stated from the beginning.

The authors have to make this very clear throughout their manuscript. As it stands right now, it is not mentioned or carefully hidden in a very few selected words hidden through the manuscript. To a reader not deep into the problem, pretty much the whole manuscript seem to report that the same kind of result can be achieved 100 times faster. Which is not the case.

To make our point clearer, we added in the introduction,

"With the prior knowledge of chemical states presented in the sample, we reduce the number of unknown spectral variations with energy down to the number of states,

thereby requiring much less data points. A full measurement will be still needed if this dimension reduction cannot be made."

What the authors phrase as "Of course, adding more data always helps to improve the result, and the difference between methods would diminish, but that's not the goal of this work.", I would phrase as: adding more data always to improve the result, and the difference between any of the methods and the GT diminishes as finally sufficient data is recorded.

We agree.

In summary: unless the authors are verbose in the discussions of the key differences and shortcomings of the presented method throughout the whole manuscript, I can not advise publication of the manuscript.

As long as the reader needs to be proficient enough in the field and the math to read between the lines to know that …

We hope we have made our points clearer in the text.

• compared to previous methods, this one requires prior knowledge of the spectra components to solve for

We have emphasized this point at multiple places including in the introduction.

• that real pure randomness in the angle-to-energy selection is worse than even interlaced with a randomized, but repeating sequence.

We appreciate the reviewer's observation that the interlaced–randomized spectrum can yield a slightly lower MSE. This is precisely why we included Table I to benchmark performance across different scenarios. However, this does not change our main conclusion: **randomizing the spectrum is essential**.

With a randomized spectrum, all sampling schemes show improved performance. In particular, the interlaced–randomized approach achieves the lowest MSE, although the difference compared with the stochastic angle–energy strategy is negligible. This result further supports our central claim that the key factor is the use of a randomized spectrum.

We evised the text to make this point even clearer. See our previous responses.

• that making a smart sub-selection of energies is possible (because the spectra are known), not hard (orthogonality of the resulting sub-spectra), and always advisable.

Again, this paper addresses the question of **whether it can be done**, rather than **how much it can be improved**. We have already emphasized that energy selection is an

important factor for further improving the method, and this topic will be addressed in a follow-up report.

• the problem is way under-determined (effectively just one sinogram and in many examples not even enough angles according to Crowther limits for a single spectral component

We explained why – dimension reduction and implicit/explicit constraints implied by the physical model, solver, and regularization.

• that taking more / closer to / exactly N_spectral times N_crowther measurements improves the quality in all cases and (thus) also makes differences between the methods disappear

Again, in fluorescence-based scanning tomography, the main limitation is the available acquisition time—and, correspondingly, the radiation dose—which constrains the amount of data that can be collected. We agree that if sufficient data could be acquired, there would be no need for advanced reconstruction algorithms, and a simple method such as FBP would be adequate. However, in practice, we do not have that luxury.

• That doing it this way is maybe the only option in case of limited time. But that it is sub-optimal and the obtained results CAN NOT be equivalent in quality to “doing it right”.

We strongly disagree. There are many scenarios in which measurements must be performed rapidly, for example to achieve temporal resolution in in situ/operando studies or to stay within radiation dose limits. In such cases, the key question is whether the measurement is feasible at all, rather than whether the reconstruction quality is optimal. This is precisely why advanced algorithms and methods are needed—to improve results under non-ideal conditions.

… I can not advise publication of the manuscript.

Once these things are very openly and very very clearly discussed in the article, I could advise a publishing of the results.

We hope that we have clarified the points and addressed all of the reviewer’s concerns.

We greatly appreciate the time and effort that Reviewer 3 devoted to carefully checking the details of our work and examining it from different perspectives. The reviewer's comments have helped us strengthen the paper. Below, we provide our response to each comment.

(1) Comment 1

The reviewer's statement is correct if information extraction is limited to the measured data alone: in principle, at least N known quantities are needed to solve for N unknowns. This is the case for direct tomographic reconstruction methods, such as filtered back projection (FBP), which directly invert projection data into object space. These methods rely solely on the measured data, are deterministic, and are objective. If enough high-quality projections satisfying the Crowther criterion can be acquired, then this is generally the preferred approach.

However, real-world measurements often involve constraints: in some cases, it is not possible to acquire enough projections, or the signal-to-noise ratio may be limited. In such situations, prior knowledge becomes important because it can help mitigate the effects of missing information or noisy data. By formulating the reconstruction as a constrained minimization problem, either with explicit or implicit constraints, we restrict the solution space to a smaller subdomain and reduce the effective degrees of freedom. For this reason, iterative methods are known to be less strictly limited by the Crowther criterion. This idea is well established; the development of algebraic tomographic reconstruction methods using fewer projections dates back to at least 1970 [https://doi.org/10.1016/0022-5193(70)90109-8].

As a simple example, we provide below comparisons between reconstructions obtained using FBP and ADMM when the number of projections is significantly smaller than that required by the Crowther criterion. In the first case, ADMM can reconstruct the phantom nearly perfectly with only 32 projections while preserving fine details. Thus, the Crowther criterion does not need to be strictly satisfied in order to achieve an accurate reconstruction in this case. This is because the phantom consists of separated domains with sharp boundaries, and total variation (TV) regularization effectively constrains the solution toward such structures. A lot of scientific samples fall into this category.

In the second case, where the object contains more complex variations, ADMM still produces a better reconstruction with substantially fewer artifacts; however, the improvement in resolution is more limited. TV regularization is less effective for this type of object, and a more advanced regularizer (such as deep neural networks) or fusion of other modalities would likely be needed to further improve the resolution. We have uploaded the code to the github (Sparsity_test) so that the reviewer can also verify these results.

[Figure Redacted]

The main drawbacks of iterative methods, aside from their computational cost, are that they can be somewhat subjective. Hyperparameter tuning is often required, such as choosing the weight of the TV term, in order to obtain the best reconstruction. In this tuning process, human judgment about what constitutes a good or poor reconstruction can be viewed as an implicit prior as well. Such prior knowledge provides information in addition to the measured data and is used to guide the solution. **We do not create information from nothing; rather, the additional information comes from prior knowledge, including the solver with its forward model, the regularization term, and hyperparameter tuning. For an underdetermined problem, these elements help select one solution from many possible ones and guide the reconstruction toward the ground truth.** Compressed sensing is the area the reviewer can refer to for theoretical background.

The iterative method presented in this work helps relax this requirement, as demonstrated by the simulation examples above, although the improvement has limitations, particularly in the presence of noise. In the Supplementary Materials of the original submission, we reported the method's higher sensitivity to noise and the associated degradation in resolution. In the experimental data presented in the paper, we observed a blurring effect in spectro-tomography compared with fluorescence tomography.

To make this point clearer to readers without relevant background knowledge, we have added the following statement:

“Although the iterative method with TV regularization can relax the sampling requirements imposed by the Crowther criterion (Fig. 1e), an increasing ratio of unknowns to knowns adversely affects spatial resolution in the real experiment due to various factors. For example, noise has a greater impact on the resolution of multi-state reconstructions than on that of single-state reconstructions (Fig. S4). More advanced regularization approaches, such as learned deep neural networks, or the integration of complementary high-resolution imaging modalities will be needed to mitigate these limitations.”

We added a comparison with single state tomography in Fig. S4 to illustrate multi-state tomography is more sensitive to noise.

(2) Comment 2

First, we would like to clarify that we never claimed that “randomness” is optimal. In fact, as we noted in the original version, further optimization of energy selection is one possible direction for future improvement. Rather, stochastic energy sampling provides a convenient and effective strategy that enabled the substantial improvement observed in this work, particularly the successful decoupling of different states with minimal crosstalk. It is the key to the observed improvement, although non-optimal.

We have explained why stochastic energy sampling is helpful: it reduces the probability that the reconstruction becomes trapped in local minima associated with strong cross-talk between states. For an underdetermined problem, infinitely many possible solutions may exist, and the mathematical algorithm selects one of them by imposing additional constraints. However, there is no guarantee that the selected solution is the true one. Stochastic energy sampling helps guide the solution toward the ground truth by minimizing cross-contamination between states.

This can be understood from the perspective of reciprocal space. The Fourier transform of each projection corresponds to a radial sampling line in the reciprocal space of the sample. If adjacent projection angles are assigned closely spaced energy points, the ratios between different states will be similar; in other words, the corresponding measurements are highly correlated. As a result, an averaged reciprocal-space map, formed from contributions of different states, can produce a similar error with respect to the measured data and may therefore also appear to be a valid solution. In this situation, it becomes much more difficult for the algorithm to correctly decouple the individual states by minimizing the squared error, leading to cross-contamination in the reconstructed solution. In the extreme case where all projections are acquired at the same energy, the ratio between states remains the same over the entire angular range; therefore, no decoupling of the states can be achieved.

Stochastic energy sampling is an effective way to reduce this possibility without requiring additional processing. We included a thought experiment in Fig. S5 of the Supplementary Materials to illustrate this point in the original version. In that example, two hypothetical spectra with minimal correlation, generated randomly, outperform the case using real spectra. The thought experiment proposed by the reviewer represents a special case of the same principle. If the two spectra are perfectly orthogonal, for example [1, 0] and [0, 1], their correlation is already minimized, and there is no need for stochastic selection. **Thus, we believe there may have been a misunderstanding: stochastic energy sampling is used to achieve less correlation between adjacent projections; it is not a fundamental requirement of the algorithm.**

To make this point clearer, we have added the following text:

"This behavior can be understood in the reciprocal space of the reconstructed object. The Fourier transform of each projection corresponds to a radial sampling line in reciprocal space that contains contributions from the individual states. When consecutive energy points are assigned to adjacent projections, the relative contributions of the states remain similar, making the corresponding sampling lines nearly linearly dependent. As a result, an average reciprocal-space map over this region can still produce a small error with respect to the measured data, leading to cross-contamination between states in the reconstructed solution. In the limiting case where the same energy is used for all projections, the states cannot be decoupled at all.

Stochastic energy sampling provides a simple yet effective way to mitigate this problem. By randomly selecting energy points from the spectrum, the probability that adjacent projections have similar state contributions becomes very small, thereby reducing cross-contamination. Developing an optimal energy-sampling strategy that minimizes inter-projection correlation, including the selection of energy points, remains a subject for future investigation."

It is an over-simplified picture but explains what happens.

(M1) The reviewer's arguments are based solely on the measured data. Under these circumstances, we agree that the Crowther criterion must be satisfied to achieve the desired spatial resolution. However, as we clearly stated in our initial response, incorporating prior knowledge can relax this requirement, and we have provided simulation code to demonstrate this point. The limitation is that such an approach is problem-dependent, requiring the design of a regularizer tailored to a specific class of problems. A well-known and theoretically rigorous example is compressed sensing, where an underdetermined system can have a unique, exactly recoverable solution under certain circumstances. This has been mathematically proven, extensively demonstrated, and is widely accepted by the scientific community. Therefore, it is indeed possible to recover a unique and correct solution from an underdetermined

problem through an appropriate choice of reconstruction algorithm and constraints. A rigorous theoretical discussion is beyond the scope of this paper; however, we have provided simulation results to illustrate this point.

(M2) Please see our response to M1. Recovering a solution with full-resolution using undersampled data is possible, as we demonstrated above. A 128x128 phantom is reconstructed perfectly using 32 projects only. However, in real-world applications it is a lot more challenging due to imperfections. A redundancy of data is always helpful for better results. A more advanced regularizer than total variation (TV) will be needed. Alternatively, integrating complementary high-resolution information from other imaging modalities could also help overcome this limitation.

(M2.6) Please see the additional discussion that has been added to the manuscript. We now clearly state that stochastic energy sampling is a simple yet effective strategy for reducing correlations between adjacent projections, and an optimal strategy is yet to be developed.

Finally, to express our appreciation for the reviewers' efforts—particularly those of Reviewer #3—we have added the following statement to the Acknowledgements:

“We thank all the anonymous reviewers for their valuable comments and suggestions, particularly Reviewer #3.”

We addressed the final comments of reviewer #3 below,

1. I suggest that the authors: Add the point that unknown random directions (slices / segments in Fourier space) could be better / worse separate the chemical states than other directions.

We modified the sentence accordingly to " the probability that adjacent projections have similar state contributions becomes smaller, thereby reducing cross-contamination. However, this strategy does not necessarily minimize the linear dependence between projections; its effectiveness in decoupling chemical states could be either better or worse than that of other sampling directions."

2. - Only keep the acknowledgement of the reviewers (and special acknowledgement of reviewer 3) if they really care. It is not needed to appease me.

We sincerely appreciate the time and effort invested by all the reviewers, especially this reviewer. Although reviewers are not typically acknowledged in the manuscript, we would like to express our genuine gratitude here.

The following review is for the manuscript titled ‘Sparse X-ray Spectro-Tomography for High-Sensitivity Three-Dimensional Chemical Imaging at the Nanoscale’ by Hanfei Yan and collaborators.

In this manuscript the authors tackle the need to record large datasets for spectro-tomography. In the most general approach specto-tomography requires a fully sampled tomogram for each energy point of a spectrum of interest to reliably recover the samples energy specific response in each resolved volume element. The need to record projections in 2D, a sufficient number of projections $\mathbf{N_C}$ within a tomogram (Crowther limit) and a full tomogram for each energy position is what imposes long scan times and a wide application of the method.

In previous works by other authors (cited within the manuscript) the need for a fully Crowther sampled tomogram at each energy point was alleviated by the assumption that the sample under investigation most likely only was made up of a small number $\mathbf{N_S}$ (<< $\mathbf{N_E}$ of energy points) of distinct compounds with distinct spectral responses, without assuming what these compounds and their spectral responses are. The gain of this approach comes from the argument that each voxels spectral response in the final volume, whatever its mixture of compounds might be, can be expressed though of a base of $\mathbf{N_S}$ (or more) base vectors. The authors of that study proceeded to take the equivalent of (~ $\mathbf{N_S}$+1) fully Crowther sampled tomograms in terms of number of recorded projections. As the spectral response of the compounds / the base vectors was not fixed / unknown, the ~ ($\mathbf{N_S}$+1)* $\mathbf{N_C}$ projections were spread over all $\mathbf{N_E}$ (>>($\mathbf{N_S}$+1)) energy points, effectively lowering the angular sampling at each energy point below the Crowther limit.

The authors of the present manuscript extend on this previous result. They added one further assumption: That the number of components and their spectral responses is exactly known.

Where the previous approach had to iterate between reconstructing $\mathbf{N_E}$ angularly under-sampled tomograms, running a principle component analysis of the voxels spectral responses, limiting the number of components, enforcing the limited set of base vectors and refining the tomographic reconstruction, the presented method can take a more direct approach: directly minimizing the set of linear equations describing both the tomographic projection (Radon transform) and the energy specific response of the sample.

The authors report the main results to be: their joint spectral-tomographic reconstruction algorithm, their supposedly better performing stochastic energy sampling approach and the demonstration of both of real data.

Within the manuscript the authors provided links to both the data and the code used to obtain the presented results. As a reviewer I very highly appreciated this, as it allowed me to see what really happens within the code and to base my review responses not just on assumptions, but rather try it myself with the authors code and data.

I believe that the presented work is unique, sound in the development of the joint reconstruction algorithm and worthy of being published. I do however argue that the results are over-sold and that the manuscript requires major revisions in regards to the potential gains of the presented approach to spectral tomography before it can be published.

In the following I will list my concerns, the authors need to address in their revision of the manuscript. I will start with the major ones:

(M1) For me the biggest grievance with the manuscript in its current state is the notion that information can be created out of nowhere. To reconstruct volumes of **M** independent coefficients for **M** linear independent spectral response vectors (material components) for each vowel with a volume, at least **M * N_C** projections will have to be recorded. The claim that any smart measurement, or algorithm that does not introduce further knowledge and assumptions can recover **M** independent coefficients per voxel from only **N_C** projection without loosing resolution or inventing something within each pixels composition is just wrong. The number of measurements (knowns) needs to be equal or larger to the number of reconstructed unknowns.

The authors claim “achieving perfect reconstruction” from an under-determined problem. This just can not be.

(M2) When comparing their stochastic (“random-uniform”) energy sampling approach to the previously published “interlaced” approach, they, in my eyes, make multiple mistakes that happen to give their stochastic approach the edge over the others methods.

The “interlaced” approach was neither meant for not claiming to reconstruct **M** components from only **N_C** projections. The authors of that study rightfully recorded more than **M * N_C** unique projection angles in total. Here the authors hand the previously published energy sampling approach a problem it was not designed for.

(M2.1) The problem is not just under-determined, but the problem size and chosen spectral responses are chosen such that (i) the “interlaced” energy sampling is disadvantaged, and (ii) the assumption needed for and thus the strength of the presented joint spectral-tomographic reconstruction is totally disregarded.

(M2.2) With that I mean that the presented algorithm hinges on knowing the spectral responses for all components within the sample, which are all known prior to the reconstruction of the data. The authors explain that these spectral responses could be obtained from a fully energy sampled set of projections, taken prior to the tomogram, using PCA (This only holds true if in the projection in question the components appear in different projected pixels with linear independent coefficients. Thus it could fail simply by an unlucky sample structure and choice of projection angle).

(M2.3) The authors did not at all take into account that, knowing the spectral response of all material components within the sample, they could have selected a set of energy point where the multiple spectra show the largest differences to each other. Instead of measuring only energies that allow to separate the various components, the spectra contain many energy points were the spectral response from the materials is very similar or shows a smaller difference than at other energy points.

(M2.4) One could have picked a small set of energies with the most orthogonal spectral response vectors. Instead many adjacent energy points have very similar spectral responses as their neighboring energy points. This becomes very clear when plotting the difference of the two spectra used in the simulation:

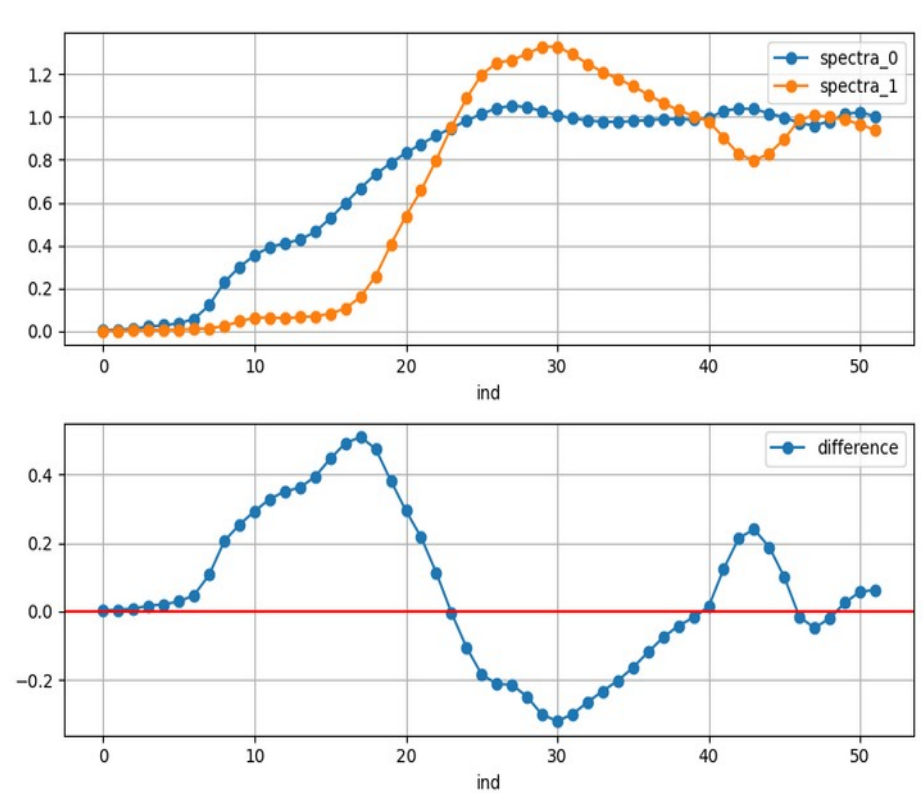

All energy points where the difference of the spectra is close to zero will show little to no contrast between the two materials. There is no reason to measure those in the first place.

Looking at the remaining energy points it becomes clear that there are stretches of adjacent energy points where the contrast between the, in this case, two materials is similar.

Measuring those groups adjacent energy points with similar responses at adjacent projection angles means there is no way to differentiate well between the two material components for those angles. That is why the interlaced energy sampling strategy is at a disadvantage here to the “uniform-random” strategy, where there is a higher chance to jump between points energy that are to each other linear independent in their spectral response, meaning it is more likely that the two material components can be separated for those adjacent projection angles (this arc of angles).

(M2.5) I argue (and I tested) that just randomizing the order of the used 52 energy points, but keeping that same order within each interlaced arc does have the same positive effect. Additionally it does still come with a homogeneous angular sampling for each energy, avoiding that by chance nearby projection angles are measured by the same energy, resulting in an unavoidable under-sampling of those energies from different projection angles.

The best results I achieved by combining a sampling strategy that gives constant angular sampling within one energy and only measuring energy points from the known spectra that allow the best possible separation of the two material components.

Below my results using the authors simulation and reconstruction code:
From left to right I varied the number of energy points of the available spectra I ended up using. On the far left, the most extreme case of just selecting two energy points to differentiate the two material compoents.

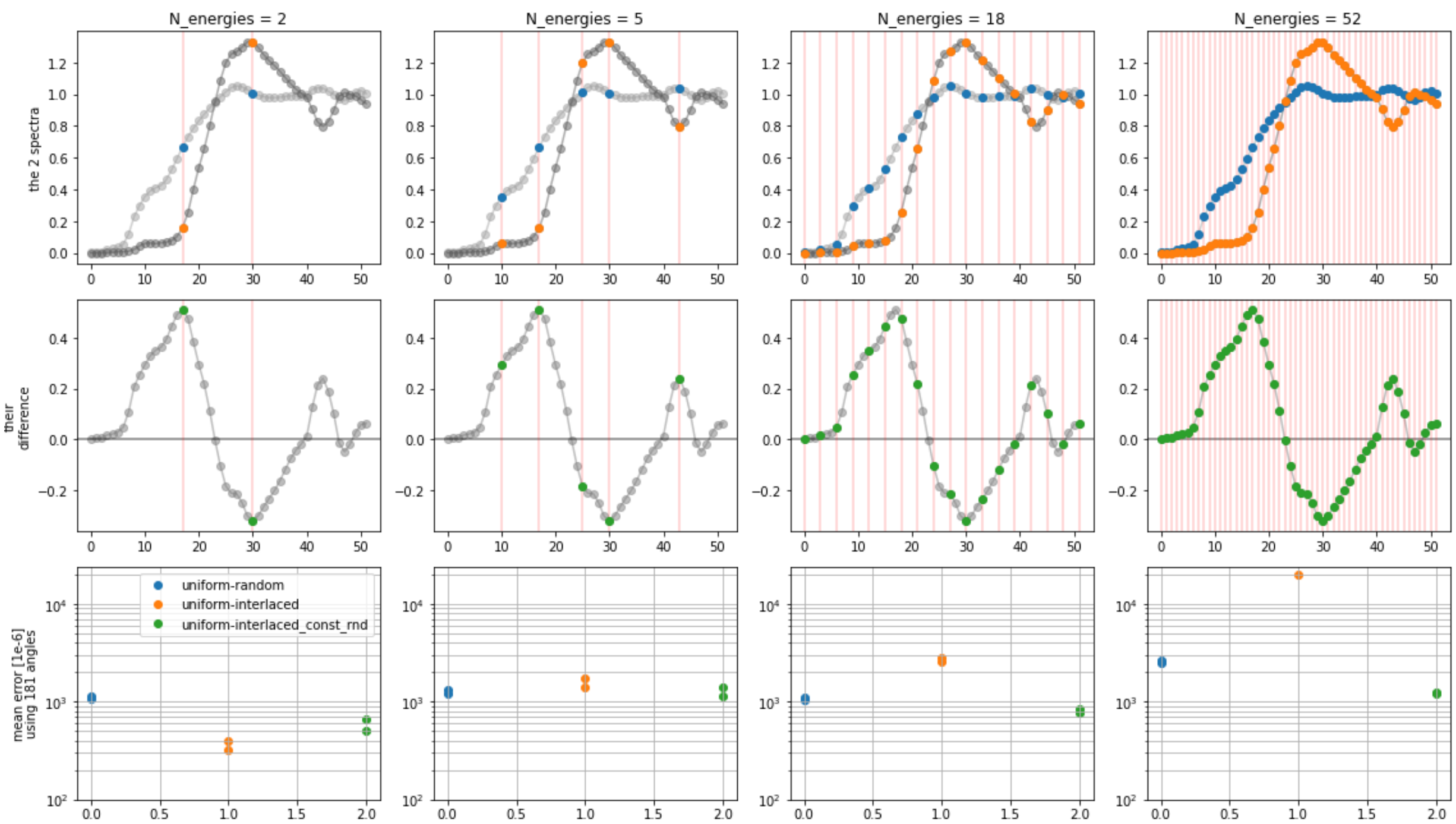

In the bottom row, I plotted the mean absolute difference in pixel value between ground truth and the reconstructed objects. The color of the dots indicated the energy sampling method used:

blue – the authors stochastic method
orange – interlaced
green – interlaced, but at a once randomized order

(M2.6) In all cases I used the (to few) 181 projection angles (as the authors) and only used values within the circular disk (not the corners of the reconstructed array) to evaluate the quality of the construction.

No matter how many energies were used, mixing the initial order of the energy points and going through them again and again (green) does result in equal or lower errors than just rolling the dice for each angle (blue). Measuring fewer and fewer unnecessary energy points does reduce the error achieved by simple “interlaced” energy sampling (orange).

The results become even better (for all three strategies) if a sufficient number of projection angles (> **2*N_c** in this case) is recorded.

While I do not believe that the authors chose the simulation parameters (128x128 pixel objects, 181 angles, 52 energy points) on purpose to have their method come out on top, this choice is the reason their algorithm worked better in this case. I want to stress that it was a sub-optimal choice to “run the experiment”.

I want to follow by some less sever, but still important concerns that require addressing:

(m.1) All figures in the manuscript are from rather low number of pixel data. That is the nature of the simulated and recorded data. And that is fine. But having not having the default sub-pixel interpolation of matplotlib.pyploy switched off does introduce a smoothness in the data that is just not there and might trick some readers. The authors should use the optional argument *interpolation='None'* in all their figures to show square pixels of homogeneous value.

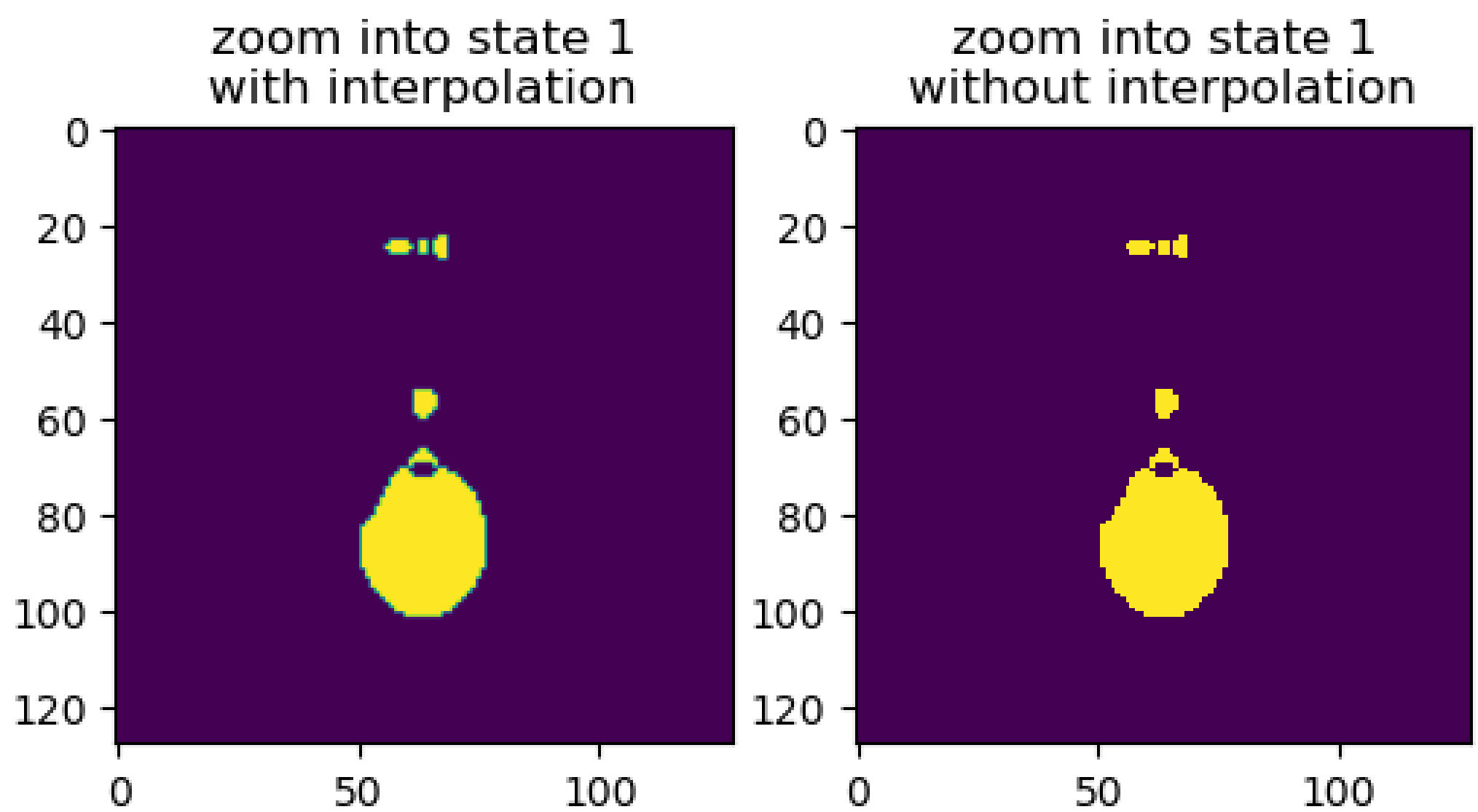

(m.2) In and around Figure 2 the authors present a simulation of a two-compound test sample. In line 139 they claim achieving “perfect” reconstruction. While the word “perfect” in itself can not supported (see above), this could not possibly be seen from the figure as it is.
I suggest that the authors plot the difference between the ground truth and their reconstructions in a diverging colormap. Additionally they should find some error metric to report the non-zero difference between GT and reconstructions with some quantitative number. Running the authors unaltered code and plotting the difference between GT and reconstructions as suggested, it becomes obvious that there is a non-zero difference (examples shown within the next comment).

(m.3) When presenting a new method based around a random number generator it is good practice to study if the seed of for the random number generator can have any impact on the quality or even consistency of the obtained result when everything else remains unchanged.
Having access to the code of the shown simulations I just ran the simulation multiple times with different seeds for the random number generator and found the quality of the reconstruction to vary. See below a selection of very few of the different results I obtained for different seeds.

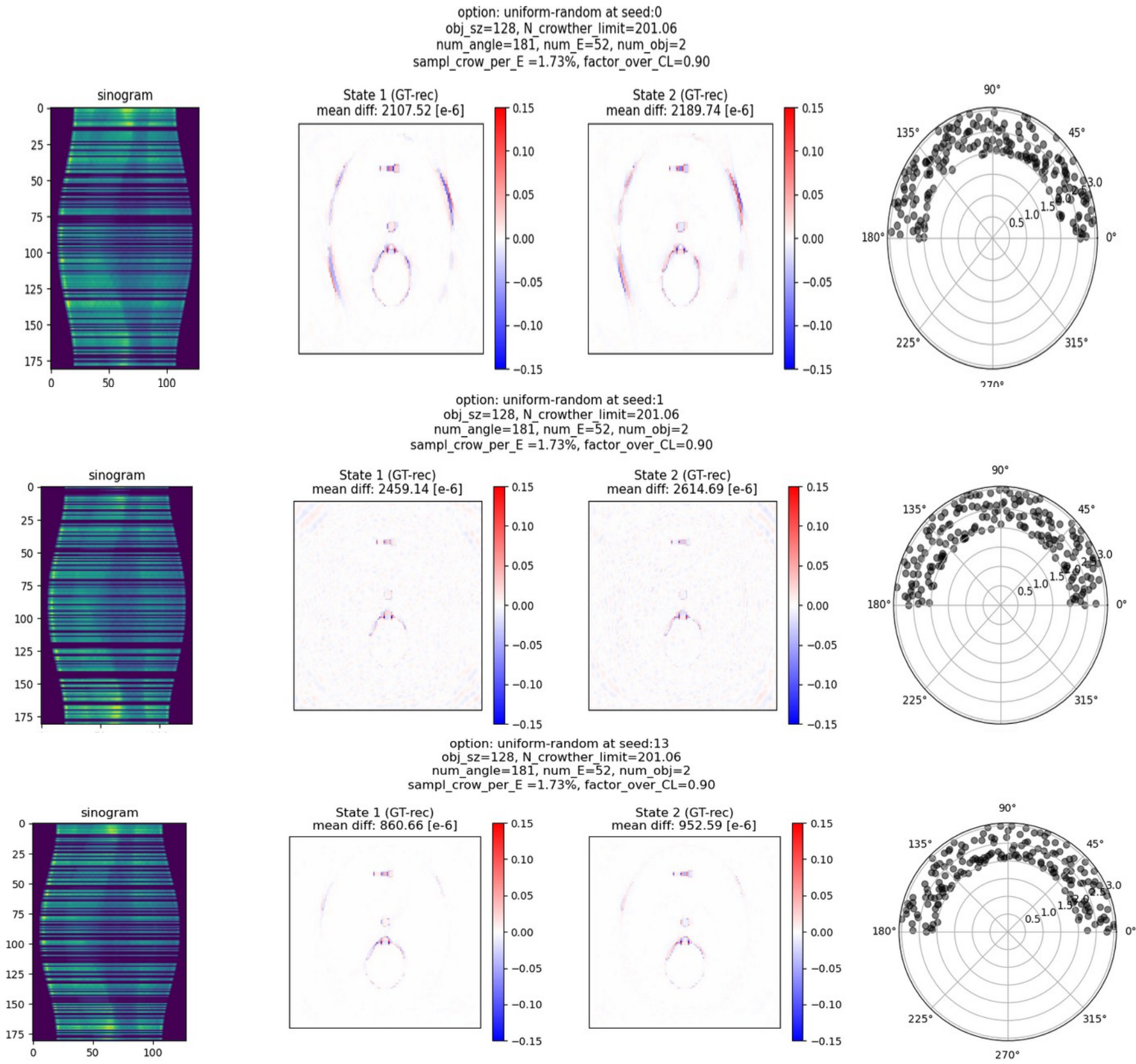

Having tried many seeds, I can say that overall many of them seem to obtain results of similar quality for the simulation at hand. But there is also a decent chance that a randomly picked seed fixes a energy to angle selection that results in multiple times larger errors.
As the selection of energies to each angle happens before the data taking, there is no way of reducing the impact of a potential bad selection purely by means of reconstructing the data differently once it is taken.
This behavior needs to be honestly discussed in detail.

(m.4) The addition of TV into their minimizer to be able to deal with noisy data was well thought out and important. But TV is “just” enforcing a certain smoothness within the obtained reconstruction / penalizing strong gradients. I would like to have seen a discussion of other options, including some that take into account the nature of the noise in the data (Poisson noise).

(m.5) The push towards joint reconstruction algorithms within the community is something I see as a very positive thing. Explaining problems as a whole and solving them as a whole as well, allows to use every bit of information wherever it might be. The authors should discuss the computational need / requirements and most importantly the scaling of their algorithm with the problem size. While playing with the authors code myself, I noticed that the minimizer does require a considerable amount of time to iterate through problems of very small size. How does the reconstruction time scale when the problem size doubles, quadruples, ect?

I want to thank the authors for the work they put into this manuscript.
I very much appreciated the way their data and their code was made available for this review.
Even if this is part of the reason my review has ended up being what it is.

I am looking forward to reading the reworked version of the manuscript to this work.
