# Supplementary material for: Sparse X-ray spectro-tomography for high-sensitivity three-dimensional chemical imaging at the nanoscale
Source: Nat Commun. 2026 Sep 16;17:10867. doi: 10.1038/s41467-026-77821-5 (PMC13672795; doi:10.1038/s41467-026-77821-5)
Supplement: Supplementary file 4 [file 41467_2026_77821_MOESM6_ESM.zip › description_of_source_data.docx]

Figure 1b

Source data for Fe0 XANES spectrum shown in figure 1b can be found in fe0_fe3_ref.txt

Figure 2b

Source data for Fe0 and Fe3+ XANES spectra shown in figure 2b can be found in fe0_fe3_ref.txt

All TIFF images are stored as 32-bit floating-point data and should be opened with a viewer that supports this format, such as ImageJ (https://imagej.net/ij/), or read in Python using the tifffile or scikit-image (skimage) package.

Figure 3a and 3b

3D reconstructions of

Fe: Fe_recon.tif

Cr: Cr_recon.tif

Fe0: Fe0_recon.tif

Fe3+: Fe3+_recon.tif

Figure 3c

Projected state maps from 3D reconstruction at different angles

Fe0: state_0_proj_series.tif

Fe3+: state_1_proj_series.tif

2D nano-XANES: nanoXANES_Fe_0_3+_40nm.tif

Figure 4a and 4c

3D reconstructions of

Co: Co_recon.tif

Co2+3+: Co2+3+_recon.tif

Co3+: Co3+_recon.tif

Mn: Mn_recon.tif

Figure 4b

Co reference spectra: Co-used-spect.txt. The two used reference spectra are labeled Co3O4 (Co 2+, 3+) and LiCoO2 (Co 3+)

Figure 4c

2D nano-XANES: chemical_map_Co3O4_LiCoO2.tif

Projected state maps from 3D reconstructions

Co_2+3+: Co_t3_0_proj.tif

Co_3+: Co_t3_1_proj.tif

Figure 5a

They are the same datasets used in Figure 4.

Supplementary Materials

Figure S12

3D reconstructions of sample 2

Fe3+: Samp_2_Fe_3_recon.tif

Fe0: Samp_2_Fe_0_recon.tif

Cr: Samp_2_Cr_recon.tif

Figure S13

2D nano-XANES of pristine particle: Pristine_XRF_Co_K.tif

Figure S14

3D reconstructions (measured at 10 keV) of

Mn: Mn_fista_norm_10keV.tif

Co: Co_fista_norm_10keV.tif

Ni: Ni_fista_norm_10keV.tif
